# Special Prey, Special Glue: NMR Spectroscopy on Aggregate Glue Components of Moth-Specialist Spiders, Cyrtarachninae

**DOI:** 10.3390/biomimetics9050256

**Published:** 2024-04-23

**Authors:** Max W. VanDyck, John H. Long, Richard H. Baker, Cheryl Y. Hayashi, Candido Diaz

**Affiliations:** 1 Department of Biology, Vassar College, Poughkeepsie, NY 12604, USA; vandyckmax@gmail.com (M.W.V.);; 2 Department of Biological Chemistry and Molecular Pharmacology, Harvard Medical School, Boston, MA 02115, USA; 3 Department of Cognitive Science, Vassar College, Poughkeepsie, NY 12604, USA; 4 Division of Invertebrate Zoology and Institute for Comparative Genomics, American Museum of Natural History, New York, NY 10024, USA; rbaker@amnh.org (R.H.B.); chayashi@amnh.org (C.Y.H.); 5 Department of Biological Sciences, Charles E. Schmidt College of Science, Florida Atlantic University, Boca Raton, FL 33431, USA

**Keywords:** spider silk, arachnology, Cyrtarachninae, bolas, NMR, aggregate glue

## Abstract

Orb-weaver spiders produce upwards of seven different types of silk, each with unique material properties. We focus on the adhesive within orb-weaving spider webs, aggregate glue silk. These droplets are composed of three main components: water, glycoproteins, and a wide range of low molecular mass compounds (LMMCs). These LMMCs are known to play a crucial role in maintaining the material properties of the glycoproteins, aid in water absorption from the environment, and increase surface adhesion. Orb-weavers within the Cyrtarachninae subfamily are moth specialists and have evolved glue droplets with novel material properties. This study investigated the biochemical composition and diversity of the LMMCs present in the aggregate glue of eight moth-specialist species and compared them with five generalist orb-weavers using nuclear magnetic resonance (NMR) spectroscopy. We hypothesized that the novel drying ability of moth-specialist glue was accompanied by novel LMMCs and lower overall percentages by silk weight of LMMCs. We measured no difference in LMMC weight by the type of prey specialization, but observed novel compositions in the glue of all eight moth-catching species. Further, we quantified the presence of a previously reported but unidentified compound that appears in the glue of all moth specialists. These silks can provide insight into the functions of bioadhesives and inform our own synthetic adhesives.

## 1. Introduction

Despite the key role that viscid glue plays in capturing aerial prey—and the potential for translation to commercial applications—the viscid glues of only a few spider species have been analyzed [1,2,3]. Given that thousands of spider species produce viscid glue in a multitude of ecological contexts, we know little about the full biological diversity of the mechanical and chemical properties of wet spider-glue droplets [2]. From a few species, we know that fluidic and mechanical properties are key to the glue’s dynamic behavior during prey capture, and that behavior is underwritten by a droplet’s chemical properties [2,4,5,6]. The basic chemical design of a glue droplet includes two components: (1) the large adhesive glycoproteins and (2) the aqueous portion that contains small soluble components, the low molecular mass compounds (LMMCs) [2,4,5,6]. The LMMCs are thought to control a droplet’s fluidic properties—most importantly, its hygroscopy, which controls its wettability when in contact with prey and its viscosity when flowing over the body of prey [7,8]. Thus, LMMCs are key to understanding how only a few species have evolved the ability to capture moths, a dynamic process that involves large droplets, high wettability, and low viscosity [4,8]. Taking a comparative approach, we measured the LMMCs for the first time in 10 species, out of total 13 species in this study, a selection that included 8 moth-catching spiders. In doing so, our goal was to identify whether the presence and composition of LMMCs in moth-catching spiders differed from those of other spiders. 

Spiders are renowned for being generalist predators with diverse niches, including active ground hunters like jumping spiders and sit-and-wait aerial predators like orb-weavers [1,2,3]. Orb-weaving spiders can produce upwards of seven different types of silk and use five of these in the construction of their webs [1,2,3]. Each silk is used for a particular job and has material properties distinct from the others [1,2,3]. Ecribellate spiders rely on viscid capture threads composed of two silks to ensnare their prey; an axial thread of flagelliform silk is coated in aqueous aggregate glue droplets [2,3,4,5,6,7]. When an aerial prey impacts a web, the thread stretches, absorbing kinetic energy, and the glue droplet provides adhesive strength [2,4,5,6]. Retention is made difficult by the insect’s flight momentum and the additional forces placed on the web as the insect fights to escape [8,9,10,11,12]. The spider must then race to its prey before it is able to drop out [8,9,10,11,12]. This places selection pressure on these glue droplets to retain prey for as long as possible to maximize capture success. One type of prey in particular, moths, has consistently presented spiders with an even greater challenge: moths are covered in a sacrificial layer of scales that flakes off upon contact with the spider’s bioadhesive. The moths thrash, peel off, and leave behind superhydrophobic scales that had worked by inhibiting the glue droplets from penetrating the surface spreading to the underlying cuticle. In this manner, these sacrificial scales limit the web’s total adhesive strength to that of the connection of the scales to the moth instead of that between the glue and the moth’s cuticle [2,3,8,10,11,12,13]. One subfamily of orb-weavers in Araneidae, Cyrtarachninae, has altered their web structure and evolved large glue droplets with novel material properties that are especially effective at keeping moths in their webs [3,8,9,14,15,16,17,18,19]. 

Differences in web structures and glue droplets between moth-specialist and generalist species lend evolutionary insight into the possible selective pressures facing moth specialists. In contrast to generalist species, moth-specialist species within the subfamily Cyrtarachninae have simplified web structures and glue droplets that lose their adhesive strength over time [4,8,13,17]. Furthermore, some webs of moth-specialists last only an evening, like those in the genus *Pasilobus*, which have triangular webs of just three radii, or those in the genera *Cyrtarachne* and *Paraplectana,* which create completely fully formed orb webs that are horizontal rather than vertical [15,18]. These genera only make webs when the relative humidity is high, thus limiting their prey capture to a particular microenvironment [8,16,19]. Under the right environmental conditions, the glue of moth-specialist *Cyrtarachne akirai* changes phase as it spreads, shifting state from a low-viscosity liquid to a high-toughness adhesive [8,17]. The simplest and shortest-lived webs are spun by the bolas spiders *Mastophora* and *Cladomelea*, which make only a single axial thread that is coated with a few large glue droplets, creating a “bolas” that is flung at nearby moths [14,17]. The glue droplets of a bolas apparently trade longevity for ease of catching moths, as they last only fifteen to thirty minutes in the field. The presence of reduced webs and specialized glue droplets associated with the novel behavior of capturing moths suggests that natural selection has targeted the molecular properties of silk and glue. But the chemical composition of the glues that enable the catching of moths remains poorly understood. Thus, investigating these glues in a comparative framework that includes generalist species can provide insight into the evolution and function of bioadhesives, informing, in turn, the design of synthetic adhesives.

Glue droplets, also called aggregate silk, are made up of water, glycoproteins, and hygroscopic LMMCs [2,4,5,6]. The aggregate glue glycoproteins, aggregate spidroin 1 and 2 in orb-weaver spiders, are thought to provide the aggregate glue with most of its adhesive strength [8,20,21,22,23,24]. However, the spider’s glycoprotein adhesiveness is highly dependent on the surrounding aqueous layer of water and LMMCs, known to be essential for solvating and maintaining the glycoprotein‘s material properties [25,26]. LMMCs, composed of inorganic salts, lipids, and small peptides [27], have been hypothesized to increase the overall surface adhesion of the aggregate glue [25,26,27]. LMMCs facilitate the expansion of aggregate proteins as water is absorbed from the surrounding environment [28,29,30]. This absorption is essential for lowering glue viscosity and allows the glue to quickly spread after contact with a substrate/prey [7,27,31]. LMMCs solvate the glycoproteins present in the aggregate glue; when they are removed, the aggregate glue’s adhesiveness is lost [25]. 

What remains unclear is what differences in biochemical composition—glycoproteins, LMMCs, or both—distinguish moth specialists from generalists. Intriguingly, across a wide range of species spidroin genes have a similar structure, suggesting that glycoproteins may not account for the differences among spiders in catching moths [22,32,33]. In contrast, generalist spiders “fine-tune” their LMMCs for maximal adhesion in their native environmental humidity [31]. Thus, an examination of the chemical diversity in the aqueous layer of the glue droplets is likely to lend valuable insight into the ability of moth specialists to catch moths. We investigated the weight proportion of LMMCs compared with the total weight of the capture threads. We hypothesized that the LMMC proportion, by silk weight, in moth specialists would be lower than in generalists, based on the observations that moth-specialist glue droplets shrink over time and a lower proportion of LMMCs might not be able to maintain their initial enlarged size. 

Several studies have shown a range of organic LMMCs across generalist orb-weaver spiders using nuclear magnetic resonance (NMR) spectroscopic analyses [25,28,29,30,34]. To date, only one study has shown the chemical distribution of organic LMMCs in a single moth-specialist species and it found novel unidentified peaks within its spectra [17]. Here, we used NMR spectroscopy to characterize and compare the biochemical diversity of LMMCs across eight moth-specialist spiders and five generalist spider species. We reconstructed the evolution of the individual LMMCs to examine if they correlated with the unique adaptive moth-catching behavior of some spiders. Thus, we tested the hypothesis that all moth-specialist spiders utilize a distinct novel chemical LMMC distribution along with the presence of at least one novel and previously unidentified LMMC when compared with generalist orb-weavers. 

## 2. Materials and Methods

### 2.1. Collection of Silk Samples

Silk samples from all spider species were collected on weighed and sterilized glass pipettes that were wrapped in aluminum foil when not in use. Our species selection included five generalists, six horizontal web-weaving moth specialists, and two bolas-spinning spiders. Four *Argiope trifasciata* spiders were collected from the Vassar Ecological Preserve and housed in the Vassar greenhouse (41.6867° N, 73.8937° W [35]). They were kept in custom-built wire and plastic cages measuring 30.5 cm × 30.5 cm × 11.5 cm. These spiders were fed moths/crickets three times per week on Monday, Wednesday, and Friday. Samples were collected three times per week on Tuesday, Thursday, and Saturday; they were taken on non-feeding days to avoid any possible contamination from crickets that were exposed to the web of the spider during feeding. 

Samples of other species were collected from various field sites by C. Diaz. Throughout September 2021, 20 *Larinioides cornutus* webs were collected in Highland Mills, New York, USA (41.3533° N, 74.1447° W [36]). These spiders had fifteen webs collected from them over the course of September. Eight *Mastophora hutchinsoni* bolas were collected from a field site at Maine Chance Farm, University of Kentucky, Lexington, Kentucky, USA (38.1211° N, 84.4873° W [37]). For *Paraplectana walleri*, approximately five webs were collected from four individuals over three nights at the Cumberland Nature Preserve in Wartburg, South Africa (29.5134° S, 30.5052° E, permit OP 2233/2022 [17]). In March 2023, ten *Cladomelea akermani* bolas and one *Cyrtarachne ixoides* web were collected at the Cumberland Nature Preserve (29.5134° S, 30.5052° E, permit OP 2233/2022 [38,39]). In June 2023, ten *Trichonephila clavipes*, *Gasteracantha cancriformis*, and *Argiope argentata* webs were collected from the Florida Atlantic University nature preserve in Boca Raton, Florida, USA (26.3770° N, 80.1055° W [40,41,42]). In August 2023, five webs of *Cyrtarachne akirai*, *Cyrtarachne bufo*, and *Cyrtarachne yunoharuensis* and three of *Cyrtarachne nagasakiensis* were collected from rice paddy fields in the Chiba Prefecture, Japan (35.6254° N, 140.4200° E [43,44,45]). 

The spider species selected were either generalists (*A. trifasciata*, *L. cornutus*, *T. clavipes*, *A. argentata*, and *G. cancriformis*) or moth specialists (*C. akermani*, *M. hutchinsoni*, *C. ixoides*, *P. walleri*, *C. akirai*, *C. bufo*, *C. nagasakiensis*, and *C. yunoharuensis*). *P. walleri*, *C. ixoides*, *C. akirai*, *C. bufo*, *C. nagasakiensis,* and *C. yunoharuensis* produce horizontal-line webs [14], while *C. akermani* and *M. hutchinsoni* are bolas spiders.

### 2.2. NMR of Spider Silk

#### 2.2.1. NMR Sample Preparation and Silk Weighting

To prepare for NMR, the glass pipettes were covered in the webs of the respective spiders (*L. cornutus* ~20 individuals, *A. trifasciata* ~22, *M. hutchinsoni* ~8, *P. walleri* ~5, *C. ixoides* ~1, *C. akermani* ~10, *T. clavipes* ~ 10, *G. cancriformis* ~10, *A. argentata* ~10, *C. akirai* ~5, *C. bufo* ~5, *C. yunoharuensis* ~5, and *C. nagasakiensis* ~3). The covered pipettes were weighed using a Mettler Toledo XS105 DualRange balance before and after silk was collected to determine the amount of capture thread obtained. For *A. trifasciata*, *L. cornutus*, and *M. hutchinsoni*, the pipette was then soaked in 50 mL nanopore water in a 250 mL beaker for 5 min to ensure the water-soluble components of the web had dissolved into the solution and then the glass pipettes were re-weighed to measure the water-soluble mass. For all other samples, pipettes were soaked in 3 mL nanopore water in 5 mL microcentrifuge tubes. The pipette substrates were then re-weighed after they had been washed and dried to determine the number of water-soluble components removed and present in our NMR solution samples. Weighting took several minutes to allow each sample to stabilize as the freeze-dried samples slowly gained weight over time based on the ambient humidity; this was in line with the intended behavior of LMMCs to absorb water from the air [8]. The resulting wash was then lyophilized using a SP Scientific VirTis Benchtop Pro (BTP-8ZL00W). Following lyophilization, the LMMCs were washed with 800 μL of 99.9% D_2_O with 0.03% TMSP (Cambridge Isotope Laboratories). After this wash, 600 μL of the D_2_O solution was transferred to a 5 mm NMR tube (DeuteroTubes) for spectroscopic testing. 

#### 2.2.2. NMR/Correlated Spectroscopy (COSY) Parameters and Statistical Analysis

The water-soluble LMMCs from the glue droplets were analyzed via ^1^H solution-state NMR spectroscopy [25,28,29,30,34]. All of the NMR experiments were performed using a Bruker Avance III 400 MHz spectrometer at 300 K. For the ^1^H experiments, the following parameters were used: ~512 scans, ~30 s delay. For the COSY experiments, the following parameters were used: ~128 scans, 3 s delay. All spectral peaks were analyzed and integrated using TopSpin (RRID: SCR_014227; Bruker GmbH) and MestreNova NMR software [46] to determine the relative percentage of each LMMC For each species, NMR chemical peaks were identified using previous literature observations and by checking peak numbers and the COSY compared to known NMR spectra in the BMRB database and Human Metabolome Database [26,47,48]. Due to the complexity of our samples, COSY was chosen to be run to distinguish the many overlapping chemical peaks from each other. In the moth specialists, the presence of a large doublet in the range of ~0.8 ppm led us to probe for potential ^1^H–^1^H COSY coupling patterns that could reveal the presence of an isopropyl group. We then quantified the percentage of each LMMC present, indexed by the subscript *i*, in each sample using the relative concentration determination (RC) as follows:

$${RC}_{i}=\frac{\frac{I_{i}w_{i}}{p_{i}}}{\sum_{i=1}^{n}\left(\frac{I_{i}w_{i}}{p_{i}}\right)}$$

where *I* is the integration intensity, *p* is the number of protons corresponding with the integrated peak, and *w* is the molecular weight of the LMMC.

Two one-way ANOVAs were run to determine if the prey capture type was a significant factor in the percentage of LMMCs present in each sample. For one test, all moth specialists were grouped and compared with generalist species and for the other, the spiders were split into three groups, generalists, bolas spiders, and horizontal-web-weavers. 

### 2.3. Ancestral-State Reconstruction of Chemical Components of Spider Glue

To identify the evolutionary changes in the aggregate glue chemical composition associated with moth-specialist predation, we analyzed the variations in the NMR signals among species within a phylogenetic framework. A phylogenetic tree was generated for all study species except *C. ixoides* (for which no gene fragments or genomic data are available) primarily based on loci used in previous phylogenetic studies [43,49,50,51]. For three species—*C. akermani*, *C. akirai*, and *P. walleri*, which lack some or all of these markers—we extracted the homologous genetic regions from genome assemblies that we had previously generated. All three genomes were generated from a single adult female using dissected silk gland tissue. *C. akirai* was sequenced using Oxford Nanopore technology and assembled using Flye v2.8 [52], while *C. akermani* and *P. walleri* were sequenced using PacBio HiFi technology and assembled using Hifiasm v.0.13-r307 [53]. The sequence data for four loci—COI, 16 S, 18 S, and 28 S (Table S1)—were aligned for all species using MAFFT [54] and concatenated into a single matrix. COI nucleotide sequences were aligned accounting for their protein translation and an outgroup species (*Tetragnatha versicolor*) was also included in the alignment (File S1). A maximum likelihood (ML) tree was generated with IQ-TREE [55] using Model Finder [56] to generate partition models for each gene [57] and 1000 bootstraps [58]. 

We reconstructed the ancestral character states of the chemical components of spider glue using Mesquite (version 3.81, build 955; [59]). We used the topology of the molecular tree (Figure S1), which was built, as explained above, from a separate molecular character set. As NMR produces ratios of a given sample, the proportion of a single constituent of a sample cannot be used as a quantitative character. Instead, we coded each of the 14 chemical components as a discrete character with the state of either present or absent. Using simple parsimony, each ancestral state on the tree was reconstructed for all characters. 

## 3. Results

### 3.1. Observations of Spider-Silk Residue and Percentage of LMMCs Lost from Web Samples

**Figure 1:**
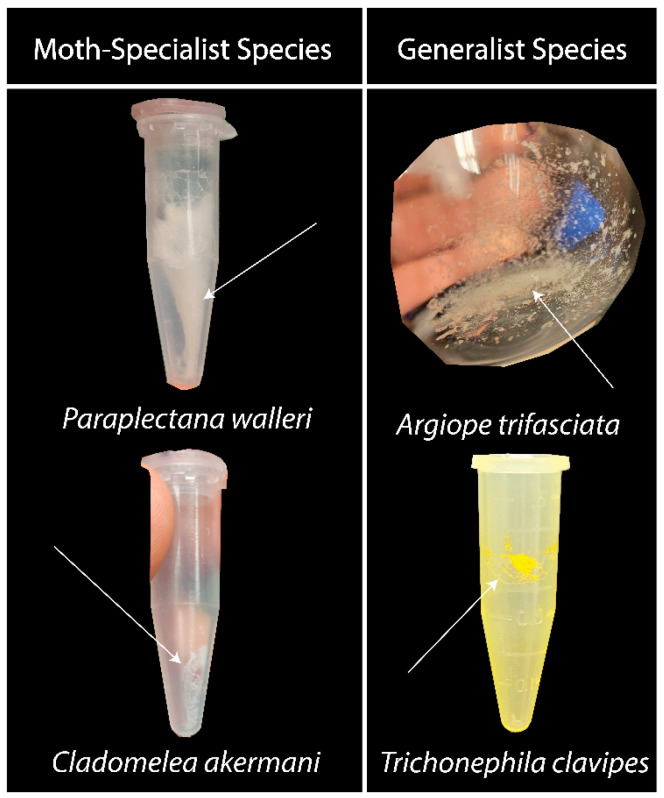
Aggregate glue and water-soluble component residue. To produce these samples, capture threads were washed and the resulting solution was freeze-dried. In these photographs, the residue from moth-specialist species appears to be different from that of generalist species. The residue of moth specialists was fluffier and more loosely packed (left column). The generalist species had a more densely packed and crystalline residue (right column; see also [26]).

The water-soluble residue of the silk of moth-specialist species had a different consistency than that of the generalists (Figure 1). Their residue was fluffier and filled the container more, layering as it dried. The generalist glue mostly settled where it was and appeared to be more crystalline. The exception was *T. clavipes*, which also contained some areas of thread-like structures.

When the species were grouped by hunting type, we found no statistical difference in the percentage weight of the water-soluble components—which were the LMMCs—washed from the web samples based on hunting types (*p = 0.4021*; Table 1). Likewise, there was no difference in this loss when compared between generalists and specialists (*p = 0.2286*). Two species, *C. ixoides* and *C. yunoharuensis*, could not have their percentages calculated because of human error; in both cases, the collection substrate was accidentally broken, resulting in the loss of some mass.

### 3.2. Solution-State ^1^H NMR of Aggregate Glue LMMCs

**Figure 2:**
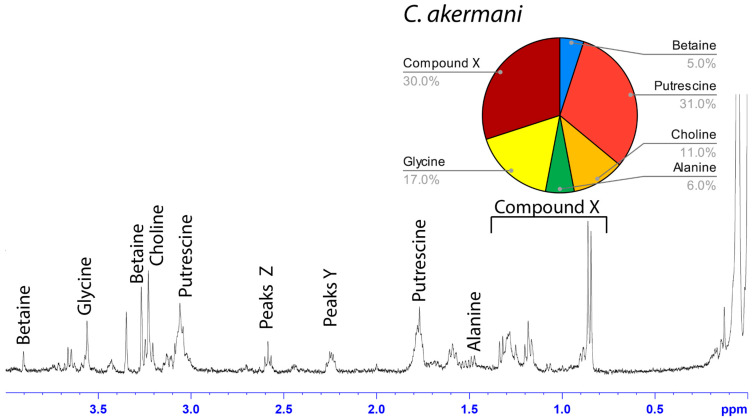
^1^H solution-state 400 MHz NMR spectrum of water-soluble LMMCs dissolved in D_2_O from the aggregate glue of *C. akermani.* The pie chart displays the relative abundance of each LMMC extracted from the aggregate glue. *C. akermani* had less chemical diversity than any other moth specialist.

**Figure 3:**
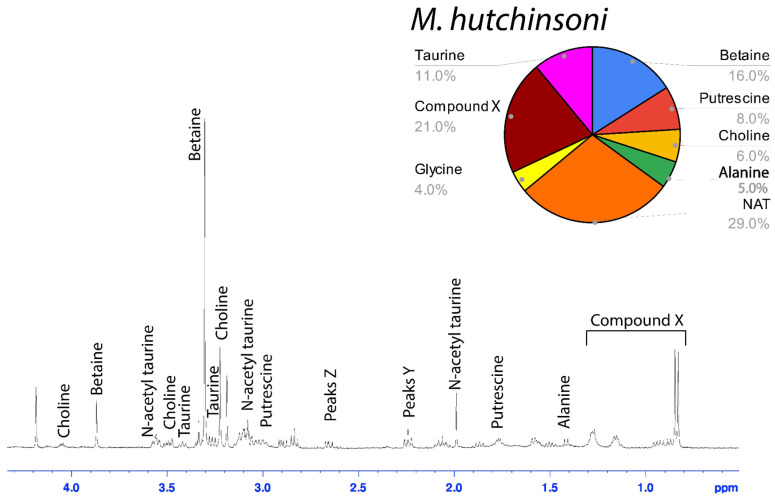
^1^H solution-state 400 MHz NMR spectrum of water-soluble LMMCs dissolved in D_2_O from the aggregate glue of *M. hutchinsoni.* The pie chart displays the relative abundance of each LMMC extracted from the aggregate glue. *M. hutchinsoni* contained taurine, which was absent in the other bolas spider, *C. akermani*.

**Figure 4:**
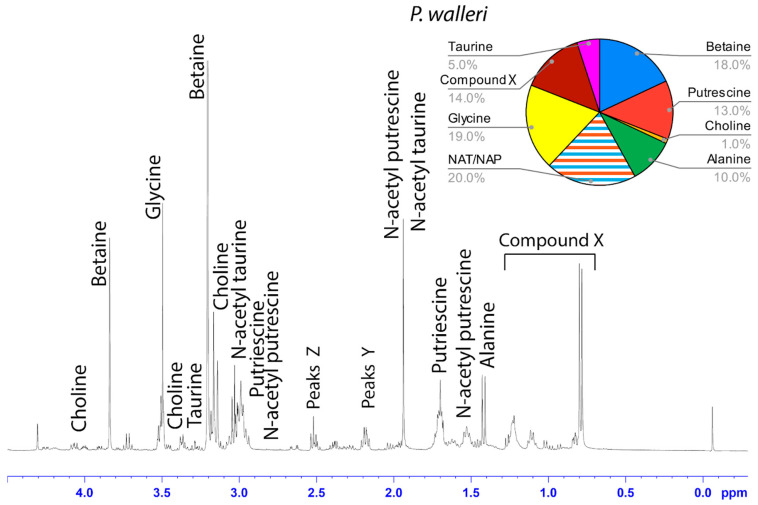
^1^H solution-state 400 MHz NMR spectrum of water-soluble LMMCs dissolved in D_2_O from the aggregate glue of *P. walleri*. The pie chart displays the relative abundance of each LMMC extracted from the aggregate glue. Due to peak overlap, we were not able to separately estimate NAT and NAP.

**Figure 5:**
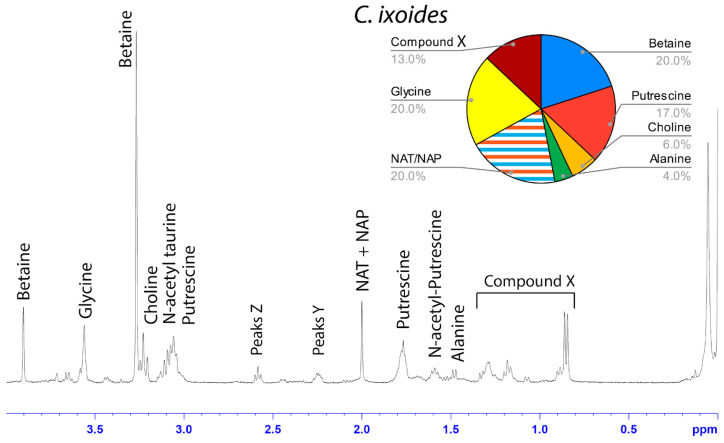
^1^H solution-state 400 MHz NMR spectrum of water-soluble LMMCs dissolved in D_2_O from the aggregate glue of *C. ixoides.* The pie chart displays the relative abundance of each LMMC extracted from the aggregate glue. Due to peak overlap, we were not able to separately estimate NAT and NAP.

**Figure 6:**
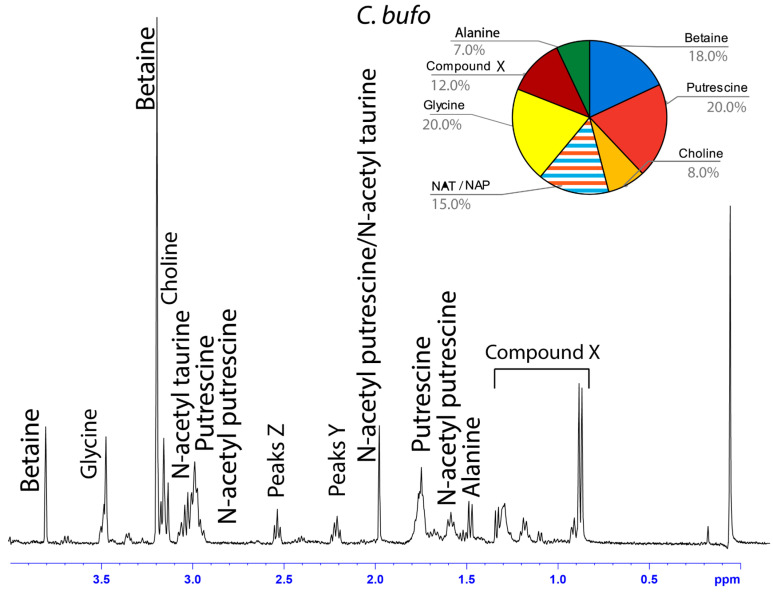
^1^H solution-state 400 MHz NMR spectrum of water-soluble LMMCs dissolved in D_2_O from the aggregate glue of *C. bufo.* The pie chart displays the relative abundance of each LMMC extracted from the aggregate glue. Due to peak overlap, we were not able to separately estimate NAT and NAP.

**Figure 7:**
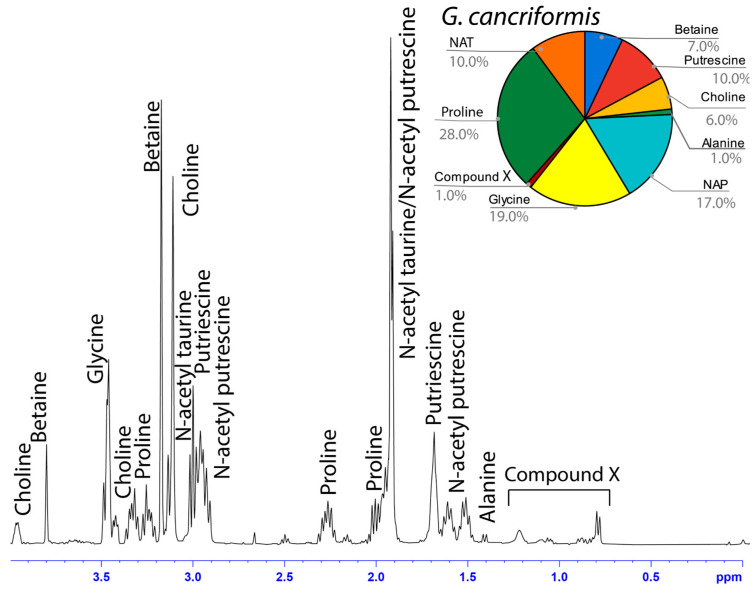
^1^H solution-state 400 MHz NMR spectrum of water-soluble LMMCs dissolved in D_2_O from the aggregate glue of *G. cancriformis.* The chart displays the relative abundance of each LMMC extracted from the aggregate glue. *G. cancriformis* was the only species to contain proline and the only generalist to contain traces of Compound X.

**Figure 8:**
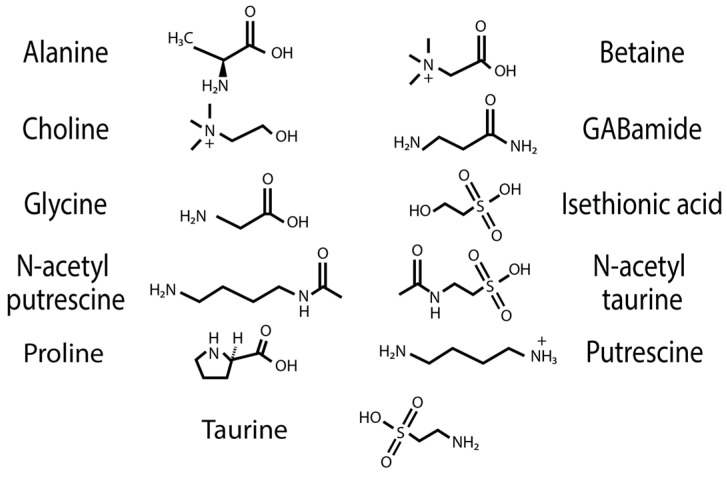
Chemical structure of water-soluble compounds identified in spider-silk samples.

The water-soluble LMMCs from the capture silk threads were analyzed using ^1^H solution-state NMR spectroscopy (Figure 2, Figure 3, Figure 4, Figure 5, Figure 6 and Figure 7). The following species were chosen to show differences between phylogenetic groups: American vs. South African bolas species *C. akermani* (Figure 2) and *M. hutchinsoni* (Figure 3); and South Africa horizontal orb-weavers *P. walleri* (Figure 4) and *C. ixoides* (Figure 5) vs. Japanese horizontal orb-weavers *C. bufo* (Figure 6) and generalist *G. cancriformis* (Figure 7). Species that had previously had an NMR analysis of their glue droplets were analyzed again and are shown in the Supplementary Figures (*A. trifasciata* (Figure S2), *L. cornutus* (Figure S3), and *C. akirai* (Figure S4)). The remaining NMR spectra are also available as Supplementary Figures (Figures S5–S8). Peaks were identified using comparisons with previous literature observations and the chemical structure of all identified peaks is shown in Figure 8 [26].

**Figure 9:**
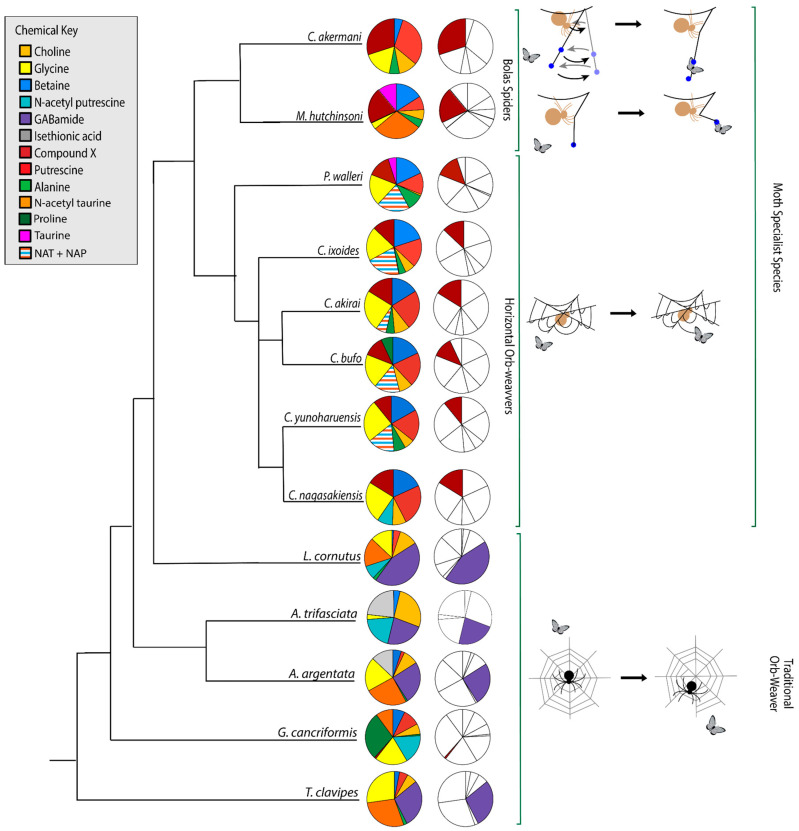
Aggregate glue LMMCs vary in proportion and presence. Moth specialists and *G. cancriformis* were the only species in which we detected Compound X (CMX; dark red). Bolas spiders *C. akermani* and *M. hutchinsoni* had larger proportions of CMX than other moth-catching taxa. Moth-specialist taxa were also characterized by a lack of GABamide (purple). The relative proportions of CMX and GABamide are highlighted in the column on the right. Overall, the glue of moth specialists was chemically distinct from that of generalist species. The phylogenetic topology is based on an ML tree created from an independent set of molecular characters (Figure S1). Some moth-specialist species had peak interference which did not allow us to separate the amount of NAT from NAP, so were estimated together (shown as a striped pattern).

The phylogenetic relationships that we determined (Figure S1) agreed with those from a previous paper [50]. When placed in a phylogenetic context, it is clear that LMMCs vary among taxa in their relative proportions, with moth-specialist taxa showing a distinct set of compounds (Figure 9). Overall, we observed differences across the spectra of generalists and moth specialists that supported the following result: moth specialists use distinct and novel LMMC distributions and compounds in their aggregate glue.

Among the moth specialists, we observed a high percentage of betaine (BET), *N*-acetyltaurine (NAT), glycine (GLY), putrescine (PUT), one unidentified compound we named Compound X (CMX), and two sets of peaks we referred to as Y and Z (PKY and PKZ). CMX occurred in only one generalist species, *G. cancriformis*, and at a much lower percentage than found in the specialists. PKY or PKZ occurred only in the specialists (Figure 7). CMX had a clear isopropyl group; through our COSY analysis, we observed the doublet at ~0.8 ppm coupled to a septet in the range of ~1.5 ppm (Figure S9). Further, the integration of the doublet and the septet were 1.00 to 0.13, respectively, allowing us to conclude that the doublet peak originated from an isopropyl group; thus, we could assign six protons to the doublet peak in our calculation of the relative percentage of CMX. Further, we estimated the molecular weight of CMX to be 100 g/mol in our relative abundance calculations by taking the average molecular weight of the known compounds in our spectra. PKY and PKZ did not have any distinguishing features in the NMR; we were unable to estimate their percentages in our samples and, thus, they are not visually represented (Figure 2, Figure 3, Figure 4, Figure 5, Figure 6 and Figure 7).

Across the generalists, we observed a high percentage of choline (CHO) and GABamide (GAB). GAB was notably absent from all eight moth-specialists’ NMR spectra. In addition, only spiders in the *Argiope* genus had isethionic acid (ISE; ~23.0%), a compound that was absent from any of the other spectra. *T. clavipes* had the highest proportion of glycine (GLY) and *N*-acetyltaurine (NAT) of any species (Figure S7). Other identified LMMCs were alanine (ALA), *N*-acetylputrescine (NAP), proline (PRO), and taurine (TAU). 

### 3.3. Ancestral-State Reconstruction from Spider-Glue Components

**Figure 10:**
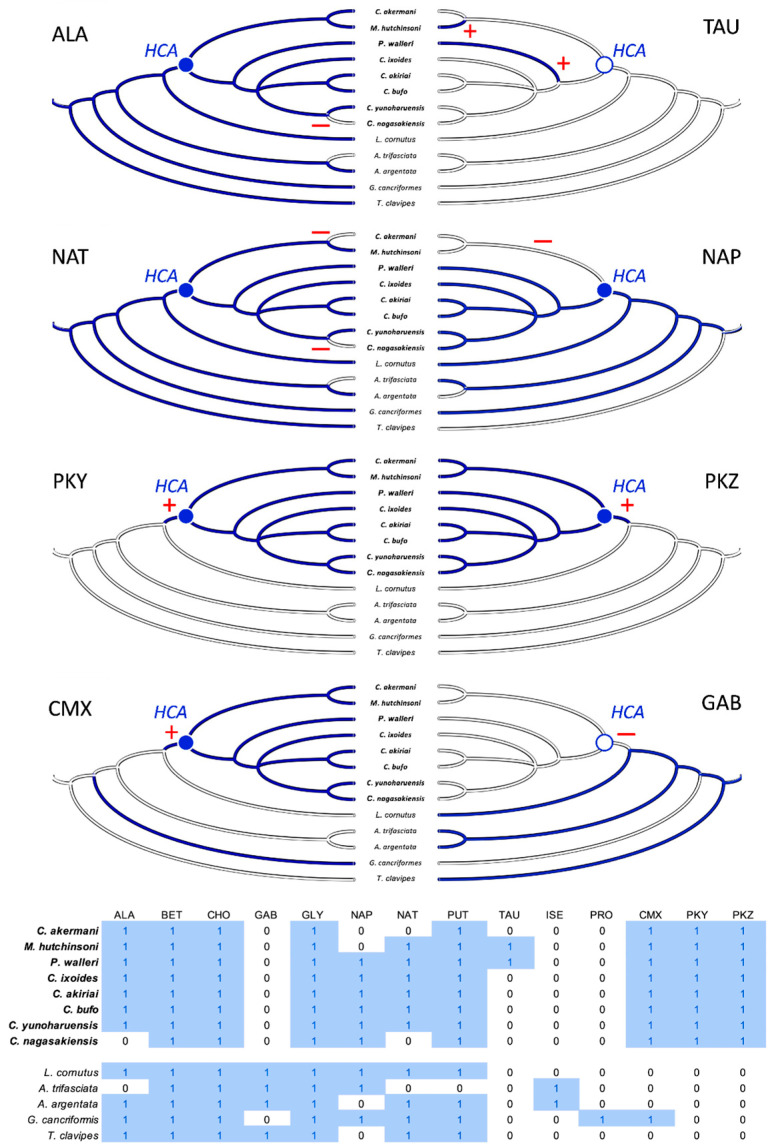
Character evolution of the LMMCs. Moth-catching specialists (taxa names in bold) form a monophyletic taxon, sharing a hypothetical common ancestor (HCA), with its variable character states reconstructed as either present (filled blue circle) or absent (empty blue circle). At or after that HCA, the evolution of a particular character state in the specialists occurs in one of three patterns: (1) loss (ALA, NAT, NAP, and GAB; red minus signs), (2) gain (PKY, PKZ, and CMX; red plus signs), or (3) convergence (TAU). Ancestral character states are reconstructed using simple parsimony, with absence indicated as a solid white line and presence indicated as a solid blue line; the one equivocal state at the root of the NAP tree is a combination of blue and white lines. The character × taxon matrix shows the coding of the characters as absent (0) or present (1) and includes all 14 LMMCs.

Each of the 14 LMMCs were coded as present or absent in a character-by-taxon matrix (Figure 10). Three characters were invariant and present in all thirteen taxa: BET, CHO, and GLY. Of the eleven variable characters, the eight that underwent a character-state change for the moth specialists were selected for the visualization of the reconstructed ancestral states (Figure 9). These were ALA, TAU, NAT, NAP, PKY, PKZ, CMX, and GAB. As shown in the character-by-taxon matrix, the remaining three constituents not figured—PUT, ISE, and PRO—had either a single loss (PUT) or a single gain (ISE and PRO). The gain of PRO in *G. cancriformis* was correlated with the independent gain of CMX, a compound that is a synapomorphy for moth-catching taxa.

## 4. Discussion

The ability of a few spider species to catch moths with their silk requires a glue with unusual capabilities not found in the adhesives of most orb-weaving spiders. Upon contact, the glue must quickly permeate a moth’s microscale surface, flow by capillary action through the meshwork between the scales and integument, and change state to a tough and elastic adhesive that glues the scale to the integument and the web to both [8,17]. While the behavior of the spider, the moth, and the glue have been described [8,17,60,61], we are only beginning to understand the chemical underpinnings and evolution of the moth-specialists’ glue. In this study, we investigated the low molecular mass constituents (LMMC) of the glue in the comparative context of moth-catching specialists and orb-weaving generalists.

Our results were consistent with the hypothesis—based on research into the spreading and setting of the glue of moth-specialist *C. akirai* [8]—that all moth-specialist species use a novel suite of LMMCs in their aggregate glue. Compared with that of five generalist taxa, the aqueous portion of the specialists’ glue had a unique combination of LMMCs, including one novel compound and NMR peaks yet to be fully characterized (Compound X and Peaks Y and Z) (Figure 2, Figure 3, Figure 4, Figure 5, Figure 6 and Figure 7). Moreover, these moth-catching species lacked GABamide, which was present in four of the five outgroup taxa (Figure 9). 

Particularly intriguing were the unidentified constituents found in the moth specialists. Compound X, also present to a very small degree in the generalist *G. cancriformis*, was particularly interesting (Figure 7 and Figure 9). Prior to this study, the glue of only one species of moth specialist, *C. akirai*, had been studied using NMR and Compound X had not been distinguished from other unidentified constituents [17]. Here, we found the same peaks in all of the specialist taxa (Figure 2, Figure 3, Figure 4, Figure 5 and Figure 6). Using a correlated spectroscopy (COSY) analysis, we distinguished one set of peaks (0.7–1.3 ppm) as Compound X, a name that reflected the fact that its precise chemical nature remains to be described. 

Based on its spectra, Compound X had an isopropyl group and was structurally similar to, but distinct from, known structures of isoleucine (Figure S8). Notably, the two bolas spiders *C. akermani* and *M. hutchinsoni* had the highest percentage of Compound X (Figure 2 and Figure 3). This correlated with the observation that bolas spiders’ glue droplets have the fastest turnover rate observed in the wild. After just 30 min on the bolas, the droplet is recycled by the spider, whereas the droplets are in place for up to 12 h for *Cyrtarachne* species. In generalist orb-weavers, the droplets may last for many days [4,8,13].

We know less about Peaks Y and Z, which were found exclusively in the specialist taxa (Figure 10) and were centered at 2.2 ppm and 2.5 ppm, respectively (Figure 2, Figure 3, Figure 4, Figure 5 and Figure 6). The COSY analysis showed they were not linked to one another, but technical limitations prohibited us from determining if these were individual compounds or peaks associated with modifications to other compounds in the sample (Figure S9B). Whichever they turn out to be, Peaks Y and Z were features of the droplet that are, for the monophyletic specialist taxon, an evolutionary novelty (Figure 10). 

Using parsimony to reconstruct the character states of the hypothetical ancestor of the moth-specialist species created a clear adaptive hypothesis (Figure 10): the combination of evolutionary gain—Compound X and Peaks Y and Z—and evolutionary loss—GABamide—facilitated the changes in glue chemistry necessary for catching moths. It is important to keep in mind that this hypothesis has yet to be tested. We simply do not know enough about the complete chemistry of the aqueous droplet and its dynamic interaction with the moth to understand the specific functional roles that these unique constituents might play. However, the striking evolutionary correlation leads us to predict that they may account, at least in part, for the droplet’s dynamic behavior (its rapid microscale permeation, fast wicking through the microscale–integument mesh, state change, and elasticity as well as tough adhesion). If this unique droplet chemistry proves to be the cause, then we will be in a position to create new substrate-specific and self-curing synthetic adhesives that can be deployed as temporary droplet traps at joints where flexibility and toughness are required and in applications with dirty and irregular surfaces.

Another feature of the droplets that is likely critical for the determination of the functional differences among species is their volume. Moth-catching specialists have droplets that are two to four orders of magnitude larger, by volume, than those made by generalists [16,17,60,62]. This means that for a given mass of LMMCs, the concentration of larger droplets is much less than the concentration of smaller droplets. This appeared to be the case in our study. We measured the mass of LMMCs from different species as a proportion of the samples’ dry weight and found no statistical differences between the specialists and generalists (Table 1). Thus, considering their aqueous state, the droplets from the specialist species were much more dilute than the generalists in terms of the concentration of LMMCs. 

Whilst our results for the three species previously investigated were largely in agreement [26], it is important to acknowledge that one species differed in terms of the presence of one compound. The generalist *A. trifasciata* contained GABamide, which had not been previously detected [26]. We also detected GABamide in *A. argentata*, which has not been previously studied. It is possible that the difference in *A. trifasciata* between studies was due to regional differences in the diet or genetics across the wide distribution of this species; we collected specimens in New York and the previous study collected specimens in Ohio [26]. Intriguingly, we identified a loss of GABamide as one of the evolutionary changes associated with the HCA of the moth-specialist taxa and as a convergent loss in *G. cancriformis* (Figure 10). It may well be that in the *Argiope* complex across its range, we are seeing variations in GABamide that is evidence of ongoing selection for the loss of this compound.

In summary, an NMR analysis of the soluble components of the adhesive droplets of spiders showed that species who specialize in catching moths have specialized glue. The moth specialists formed a monophyletic taxon with a hypothetical common ancestor (Figure 10) who appeared to have evolved one novel compound (Compound X) and lost another compound (GABamide). These are low molecular mass compounds (LMMCs), whose functional contributions to the dynamic behavior of spider glue remain unknown. Understanding the functional contribution of these LMMCs will, we predict, shed light on the natural design of silk’s adhesive properties. A water-uptake analysis, for example, would characterize the hygroscopic efficiency of these novel LMMCs, revealing how they facilitate the water-driven expansion of the glue droplet. Additionally, studying the viscoelastic properties of solutions with different compositions of LMMCs will shed light on their contribution to the properties of the glue that permit the catching and retaining of moths. Further exploration of the less polar and non-polar fractions of aggregate glue could shed light on the contribution of non-water-soluble compounds to the glue’s adhesive properties. In addition, a genomic analysis of the expression of spidroins, proteins that make up the large glycoprotein components of the glue, would undoubtedly yield information about the elasticity and toughness of the droplets. This integrated understanding will, in turn, provide insights for biomimetic engineers into bioadhesives and inform our design of synthetic adhesives [63].

## Figures and Tables

**Table 1:** **LMMCs in dry silk** (proportion by weight, right-hand column). Species are grouped by hunting strategy: generalists, bolas spiders, and horizontal moth specialists. No statistically significant difference was found between the hunting groups.

Species	Hunting Type	Dry Pipette (mg)	Pipette and Silk (mg)	Dry Pipette and Silk after Wash (mg)	LMMCs Removed (mg)	LMMC Weight Lost (%)
*T. clavipes*	Generalist	463.09	469.51	466.51	3.00	**46.7**
*L. cornutus*	Generalist	306.65	321.15	315.77	5.38	**37.1**
*G. cancriformis*	Generalist	473.1	475.88	474.02	1.86	**66.9**
*A. trifasciata*	Generalist	368.37	412.28	399.51	12.77	**49.3**
*A. argentata*	Generalist	692.78	693.76	692.98	0.78	**79.6**
*M. hutchinsoni*	Bolas	983.51	984.31	983.85	0.17	**37.0**
*C. akermani*	Bolas	664.95	665.33	665.17	0.16	**42.1**
*P. walleri*	Horizontal	1170.6	1176.89	1172.78	4.11	**65.3**
*C. ixoides*	Horizontal	397.84	398.23	398.3	−0.07	**N/A**
*C. akirai*	Horizontal	284.8	288.16	287.64	0.52	**15.5**
*C. bufo*	Horizontal	266.87	268.5	268.16	1.63	**20.9**
*C. nagasakiensis*	Horizontal	400.23	400.66	0.03	0.43	**7.0**
*C. yunoharuensis*	Horizontal	548.42	547.79	547.65	−0.06	**N/A**

## Data Availability

Raw NMR Data and data sheets used to create LMMC Table 1 and NMR pie charts are available as Supplementary Material for download (Supplementary File S1).

## Supplementary Materials

The following supporting information can be downloaded at: https://www.mdpi.com/article/10.3390/biomimetics9050256/s1. The molecular tree used to create Figure 9 is available as Supplementary Figure S1. The alignment file used to generate that tree is also available for download. Species that had NMR conducted on their glue droplets prior are *A. trifasciata* (Figure S2), *L. cornutus* (Figure S3), and *C. akirai* (Figure S4). The remaining NMR spectra are available as Figures S5–S8. Justification of our isopropyl group assumption is available in Figure S9. Raw NMR data and data sheets used to create LMMC Table 1 and NMR pie charts are available as Supplementary File S1. A file containing images of all NMR survey spectra, expanded spectra, and 2D COSY is available as Supplementary File S2.
