# Supplementary material for: Special Prey, Special Glue: NMR Spectroscopy on Aggregate Glue Components of Moth-Specialist Spiders, Cyrtarachninae
Source: Biomimetics (Basel). 2024 Apr 23;9(5):256. doi: 10.3390/biomimetics9050256 (PMC11117802; doi:10.3390/biomimetics9050256)
Supplement: Supplementary file 1 [file biomimetics-09-00256-s001.zip › NMR_Supplementary Figures.pdf]

Supplemental Table S1. Genbank IDs for all loci used for molecular tree generation. We were unable to obtain genetic information from *C. ixoides*.

	**16S**	**COI**	**18S**	**28S**
A. argentata	FJ607446.1	FJ607554.1	FJ607480.1	FJ607519.1
A. trifasciata	MK420199.1	FJ525316.1	FJ525386.1	MK425972.1
C. akermani	PP377646	PP359446	PP377642	PP377644
C. akirai	PP355199	AB820892.1	AB910450.1	AB910481.1
C. bufo	MK420214.1	AB820871.1	AB910451.1	AB910482.1
C. nagasakiensis	MK420216.1	MK420096.1	AB910457.1	AB910488.1
C. yunoharuensis	MK420217.1	MK420097.1	AB910459.1	AB910490.1
G. cancriformis	FJ525354.1	FJ525321.1	FJ525391.1	FJ525373.1
L. cornutus	MK420237.1	FJ525322.1	EU003349.1	JN816940.1
M. hutchinsoni	MK420246.1	MK420131.1	MK426129.1	MK426009.1
P. walleri	PP377647	PP359447	PP377643	PP377645
T. clavipes	MK420256.1	MK420140.1	MK426138.1	MK426047.1
T. versicolor	FJ525350.1	FJ525317.1	FJ525387.1	FJ525369.1

Supplementary Figures:

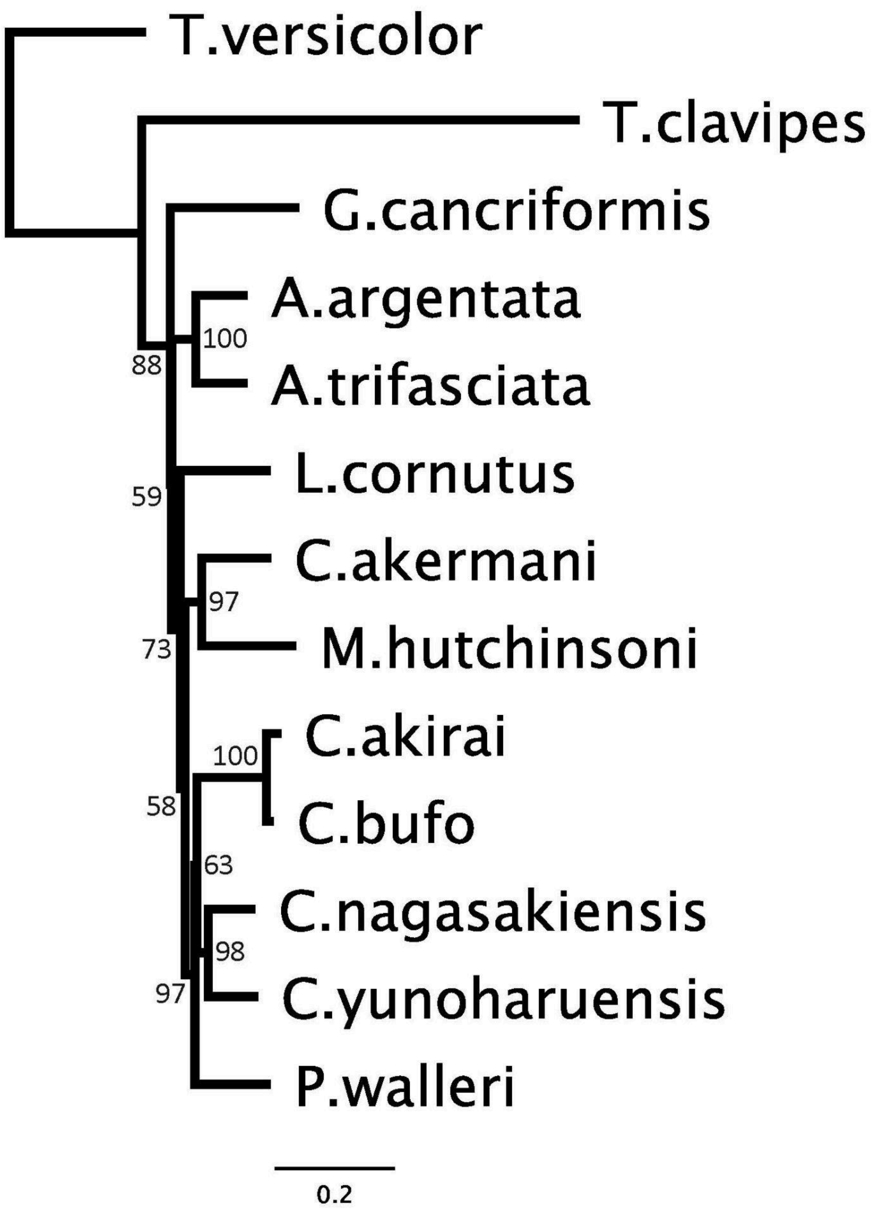

Figure S1. Phylogenetic tree. Relationships were generated from 16s, COI, 18s, and 28s loci (see Table S1). Genetic material from the moth-specialist species were collected as part of this study, while generalist species were taken from previous studies [43, 49, 50, 51]. We were unable to obtain genetic information from *C. ixoides*. *T. versicolor* was used as an outgroup in this tree; since its aggregate glue was not studied, it was not included in the main set of figures.

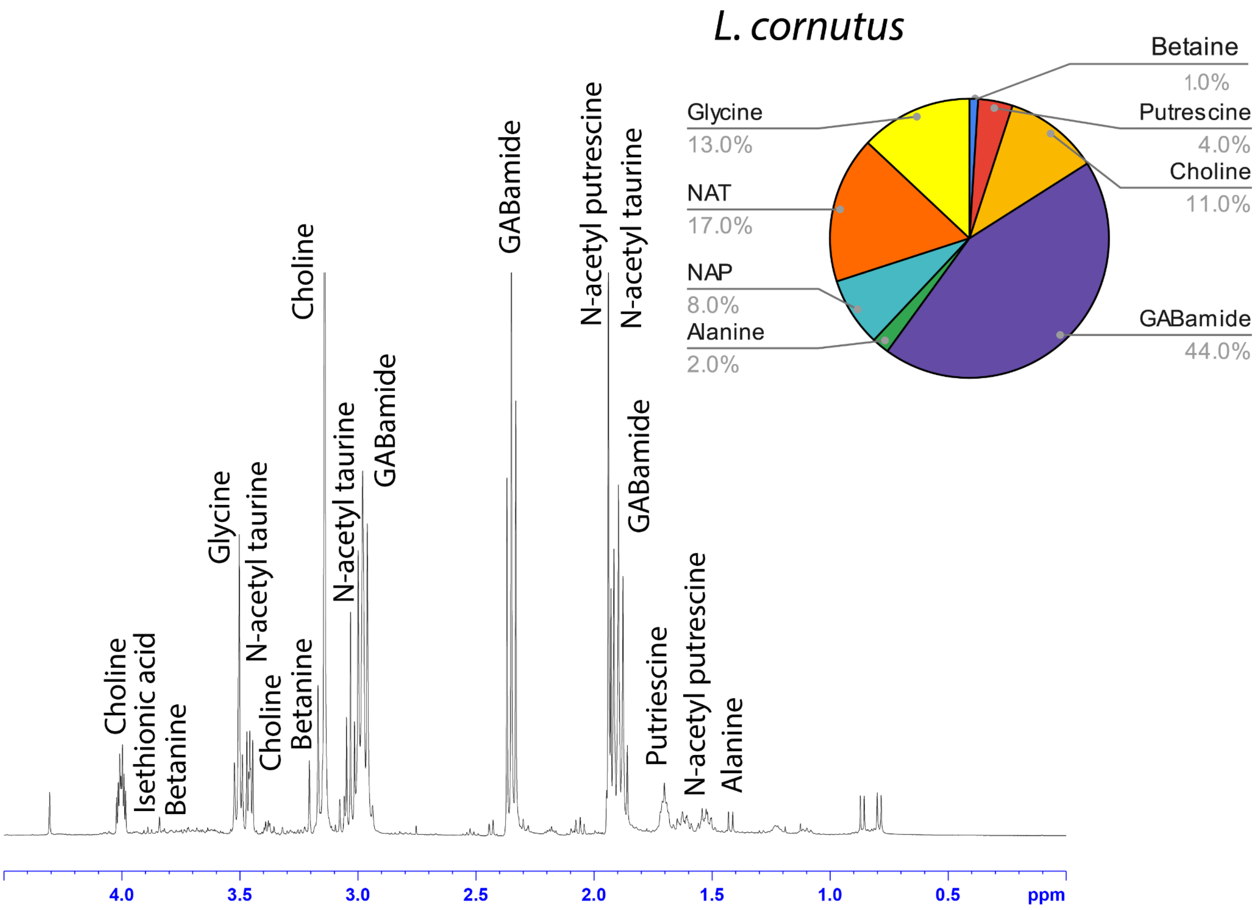

Figure S2. ^{1}H solution state 400-MHz NMR spectrum of water soluble LMMCs dissolved in D_2O from the aggregate glue of *L. cornutus*. Labeled peaks have been identified by comparison to Jain et al. (2018). The color-coded pie chart displays the relative abundance of each LMMC extracted from the aggregate glue. The abbreviation NAP stands for NAP, *N*-acetylputrescine and NAT stands for *N*-acetyltaurine.

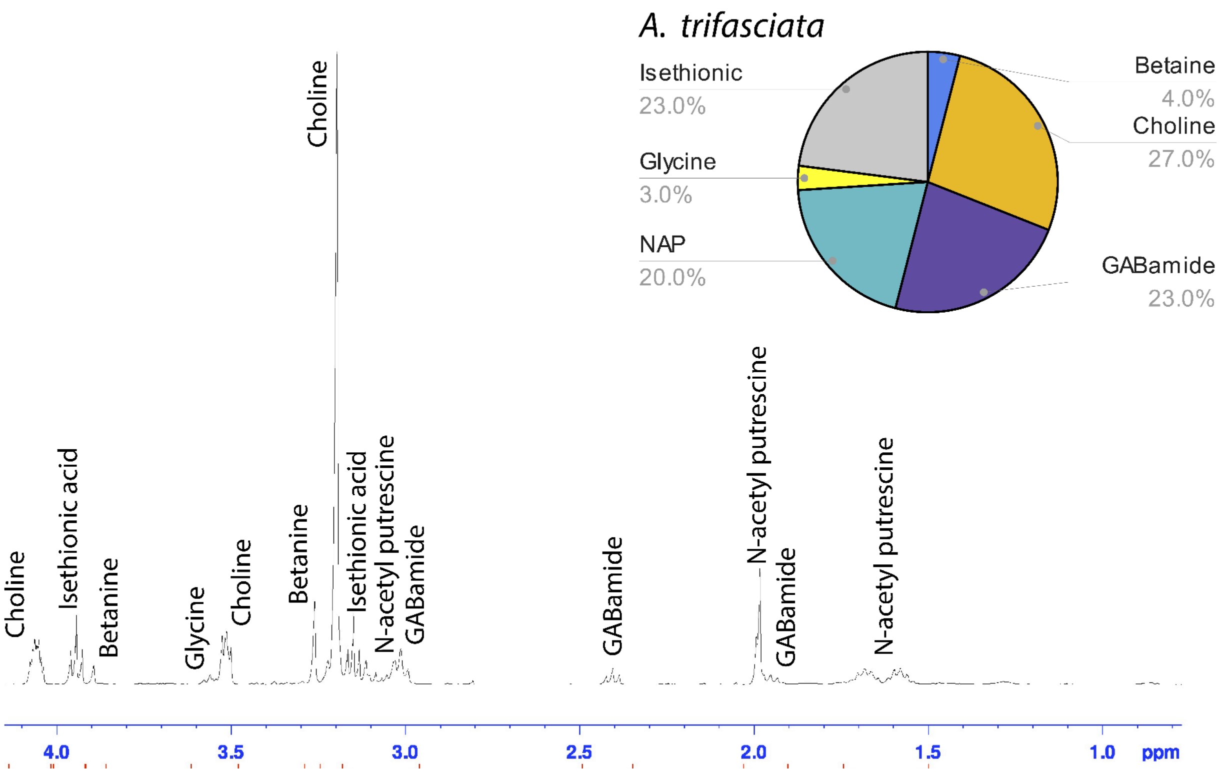

Figure S3. ^{1}H solution state 400-MHz NMR spectrum of water soluble LMMCs dissolved in D_2O from the aggregate glue of *A. trifasciata.* Labeled peaks have been identified by comparison to Jain et al. (2018). The color-coded pie chart displays the relative abundance of each LMMC extracted from the aggregate glue. The abbreviation NAP stands for NAP, *N*-acetylputrescine.

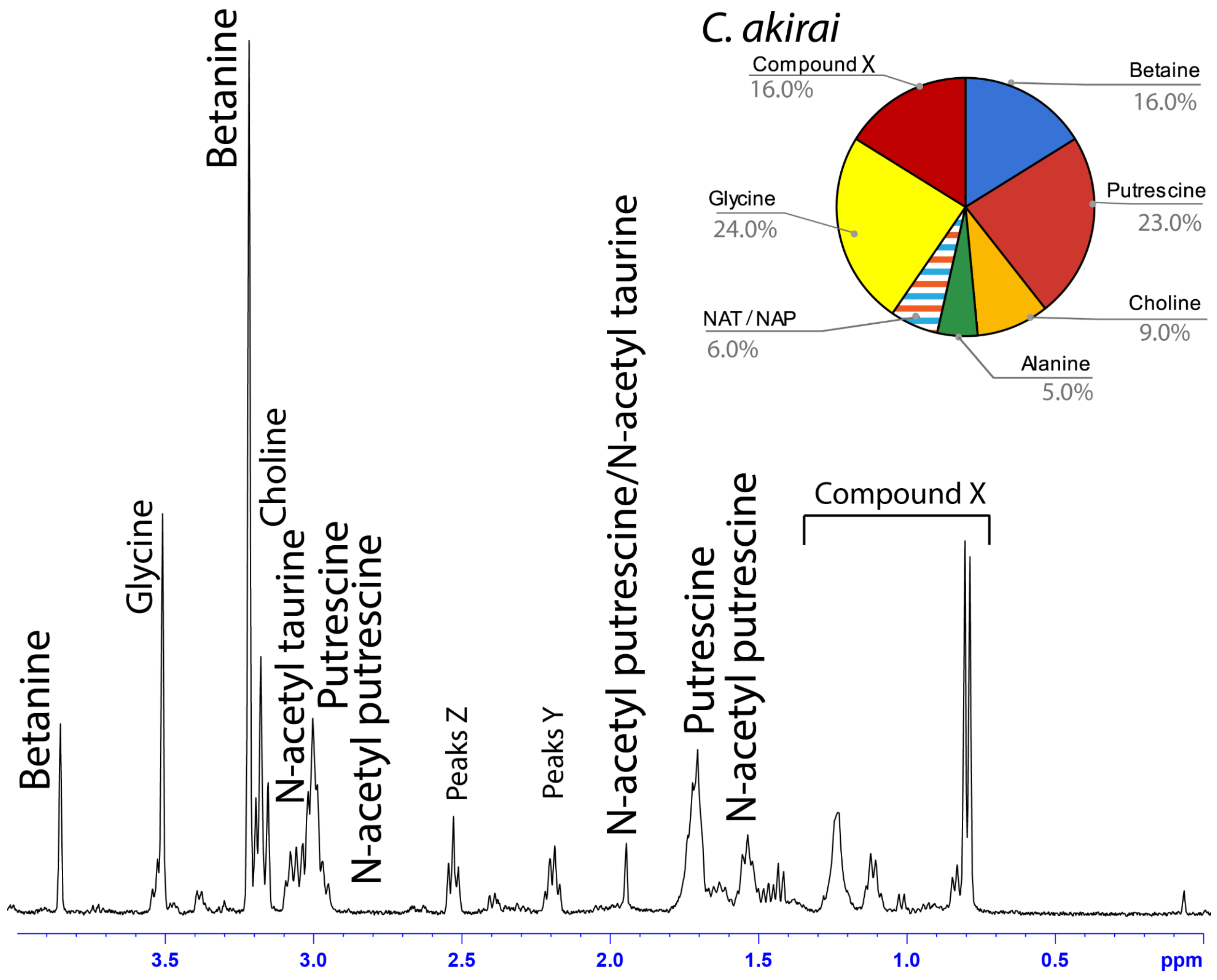

Figure S4. ¹H solution state 400-MHz NMR spectrum of water soluble LMMCs dissolved in D_2O from the aggregate glue of *C. akirai*. Labeled peaks have been identified by comparison to Jain et al. (2018). The color-coded pie chart displays the relative abundance of each LMMC extracted from the aggregate glue. We were unable to measure the percentages of peaks Y and Z. The abbreviation NAP stands for NAP, *N*-acetylputrescine and NAT stands for *N*-acetyltaurine. Due to peak overlap, we were not able to estimate NAT and NAP separately.

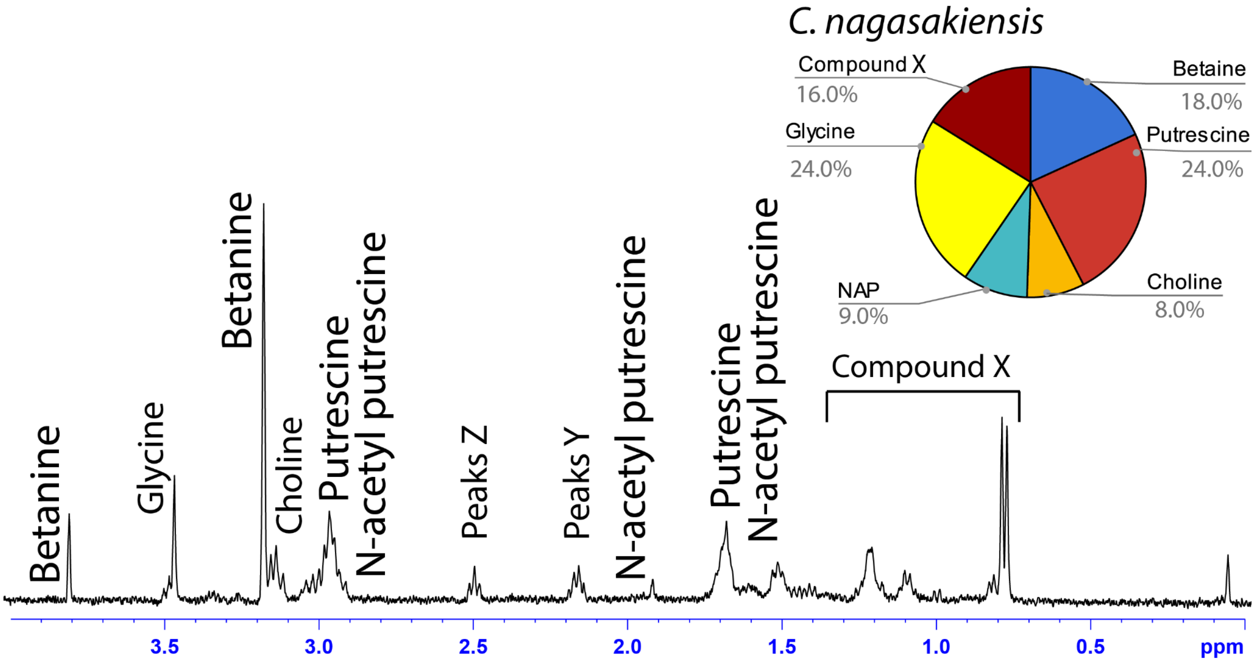

Figure S5. ^{1}H solution state 400-MHz NMR spectrum of water soluble LMMCs dissolved in D_2O from the aggregate glue of *C. nagasakiensis.* Labeled peaks have been identified by comparison to Jain et al. (2018). The color-coded pie chart displays the relative abundance of each LMMC extracted from the aggregate glue. We were unable to measure the percentages of peaks Y and Z. The abbreviation NAP stands for NAP *N*-acetylputrescine.

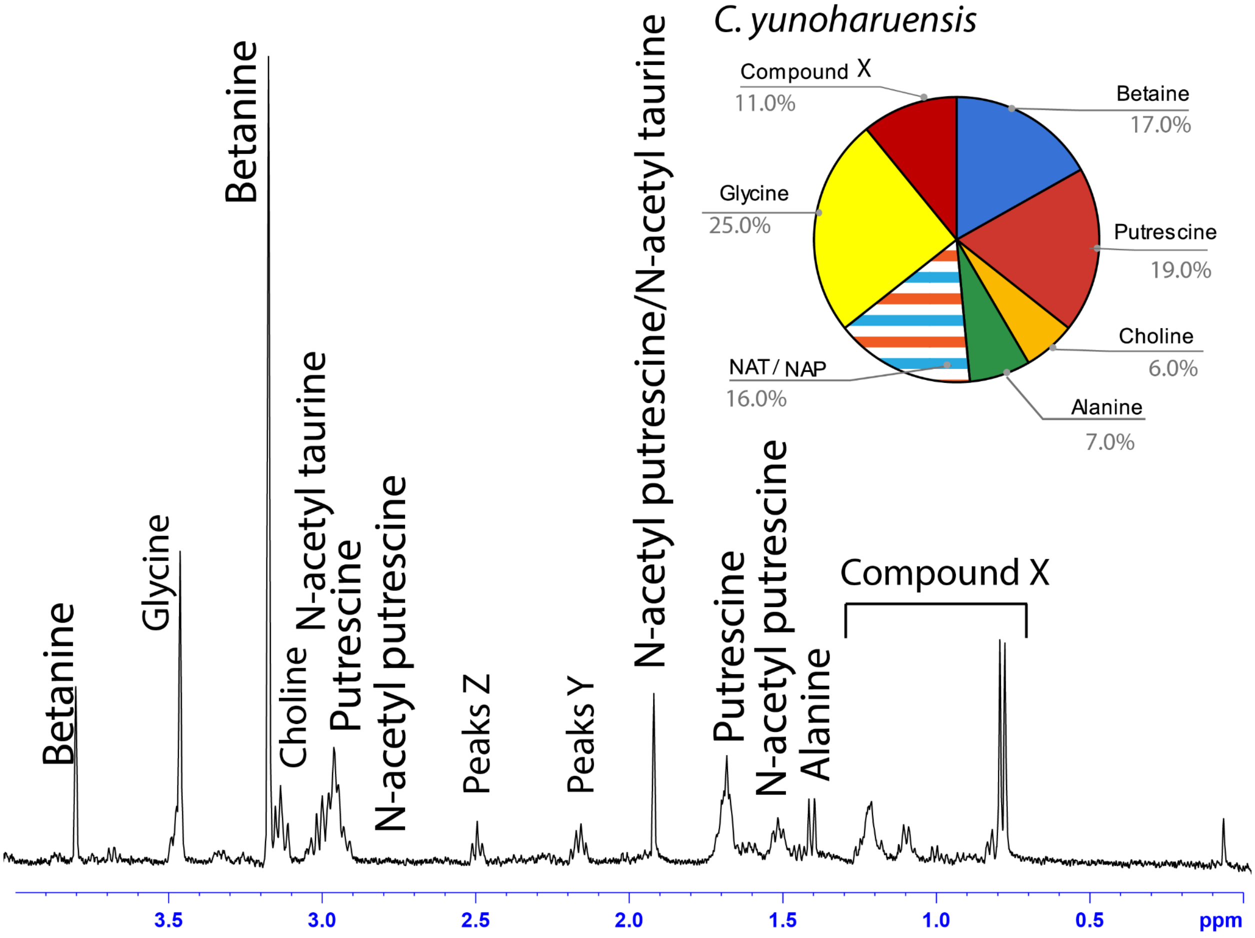

Figure S6. ^{1}H solution state 400-MHz NMR spectrum of water soluble LMMCs dissolved in D_2O from the aggregate glue of *C. yunoharuensis.* Labeled peaks have been identified by comparison to Jain et al. (2018). The color-coded pie chart displays the relative abundance of each LMMC extracted from the aggregate glue. We were unable to measure the percentages of peaks Y and Z. The abbreviation NAP stands for NAP, *N*-acetylputrescine and NAT stands for *N*-acetyltaurine. Due to peak overlap, we were not able to estimate NAT and NAP separately.

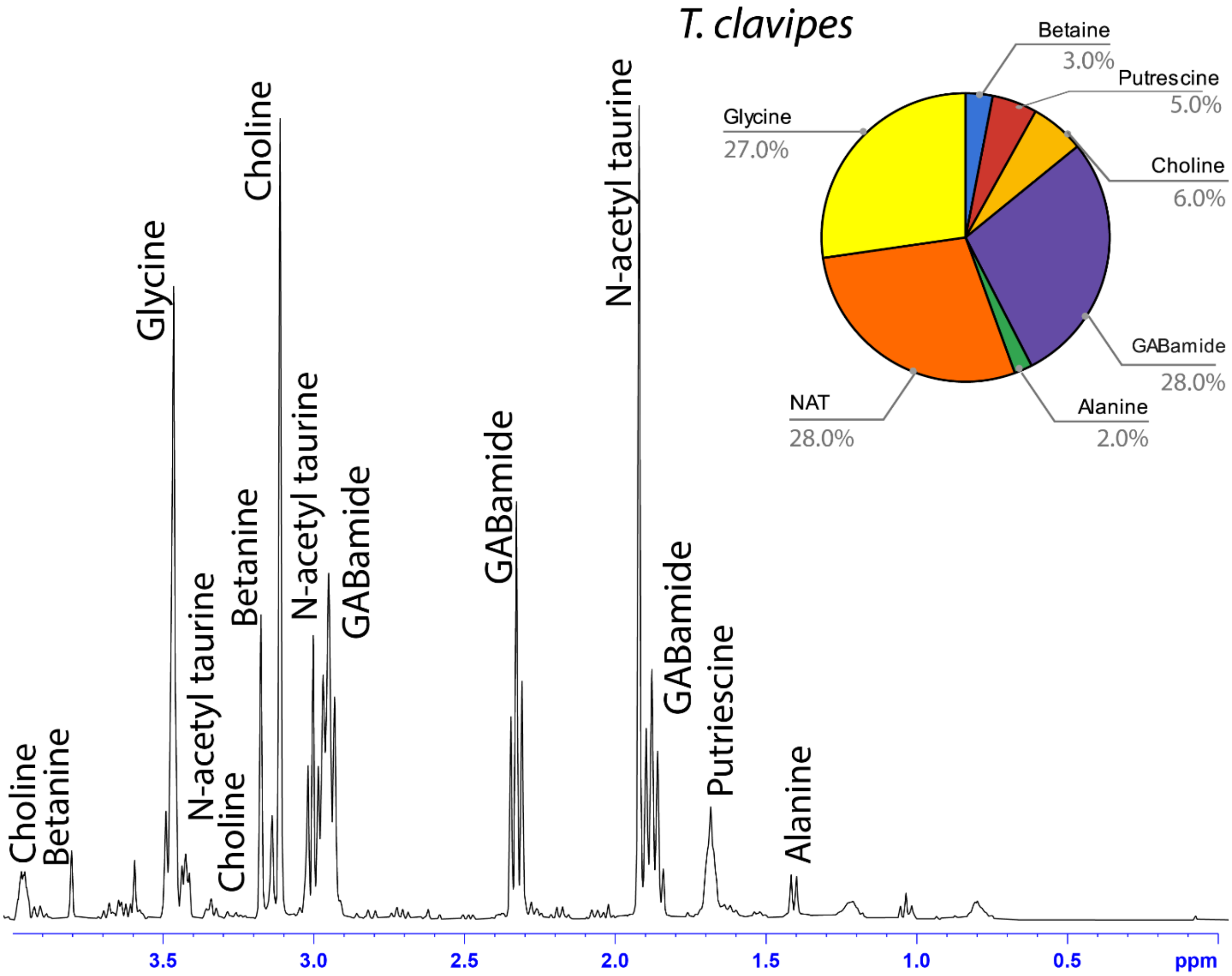

Figure S7. ^{1}H solution state 400-MHz NMR spectrum of water soluble LMMCs dissolved in D_2O from the aggregate glue of *T. clavipes.* Labeled peaks have been identified by comparison to Jain et al. (2018). The color-coded pie chart displays the relative abundance of each LMMC extracted from the aggregate glue. We were unable to measure the percentages of peaks Y and Z. The abbreviation NAT stands for *N*-acetyltaurine.

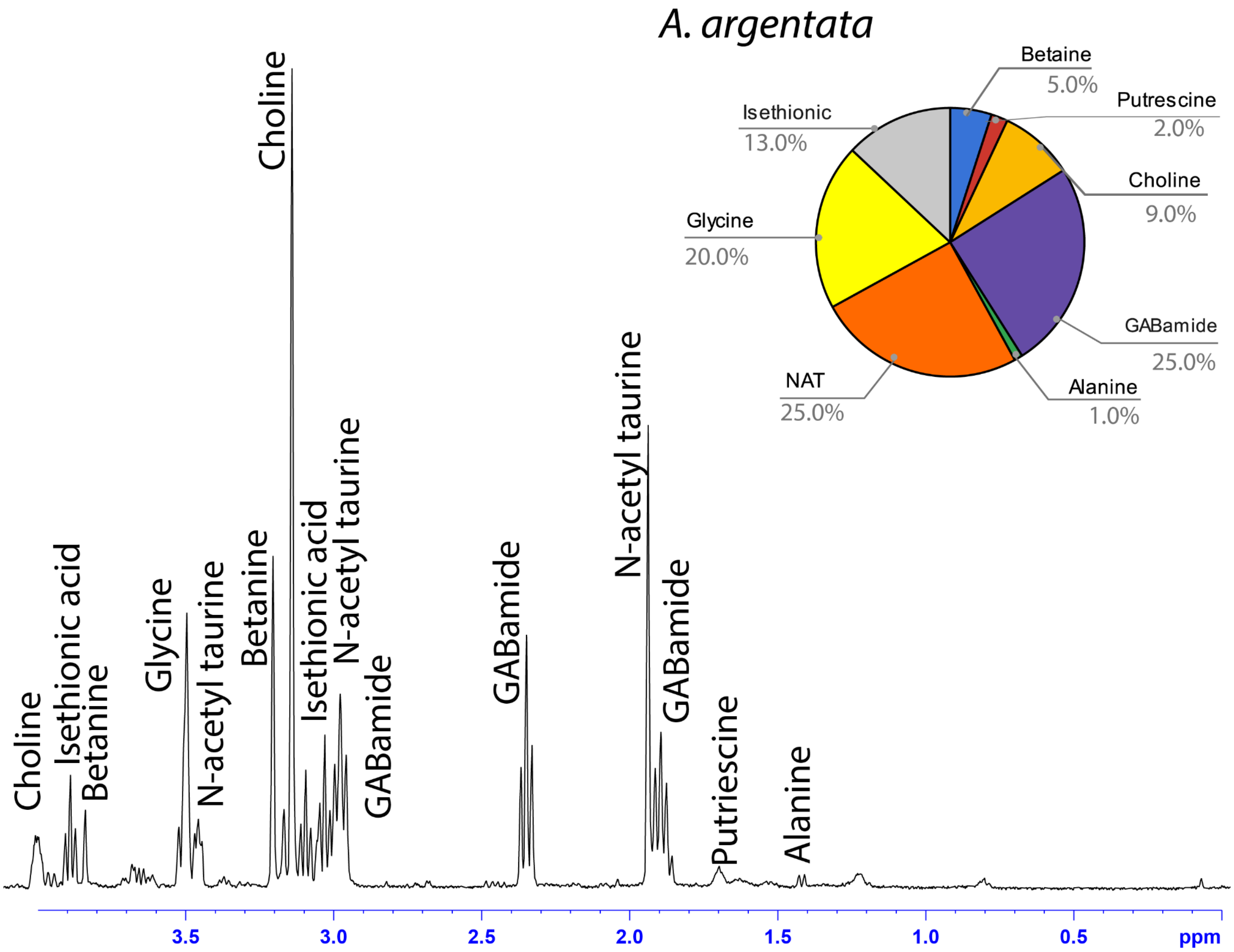

Figure S8. ^{1}H solution state 400-MHz NMR spectrum of water soluble LMMCs dissolved in D_2O from the aggregate glue of *A. argentata*. Peaks that have been identified by comparison to previously studied species are labeled. The spectrum is accompanied by a color coded pie chart that displays the relative abundance of each LMMC extracted from the aggregate glue. NAT, *N*-acetyltaurine.

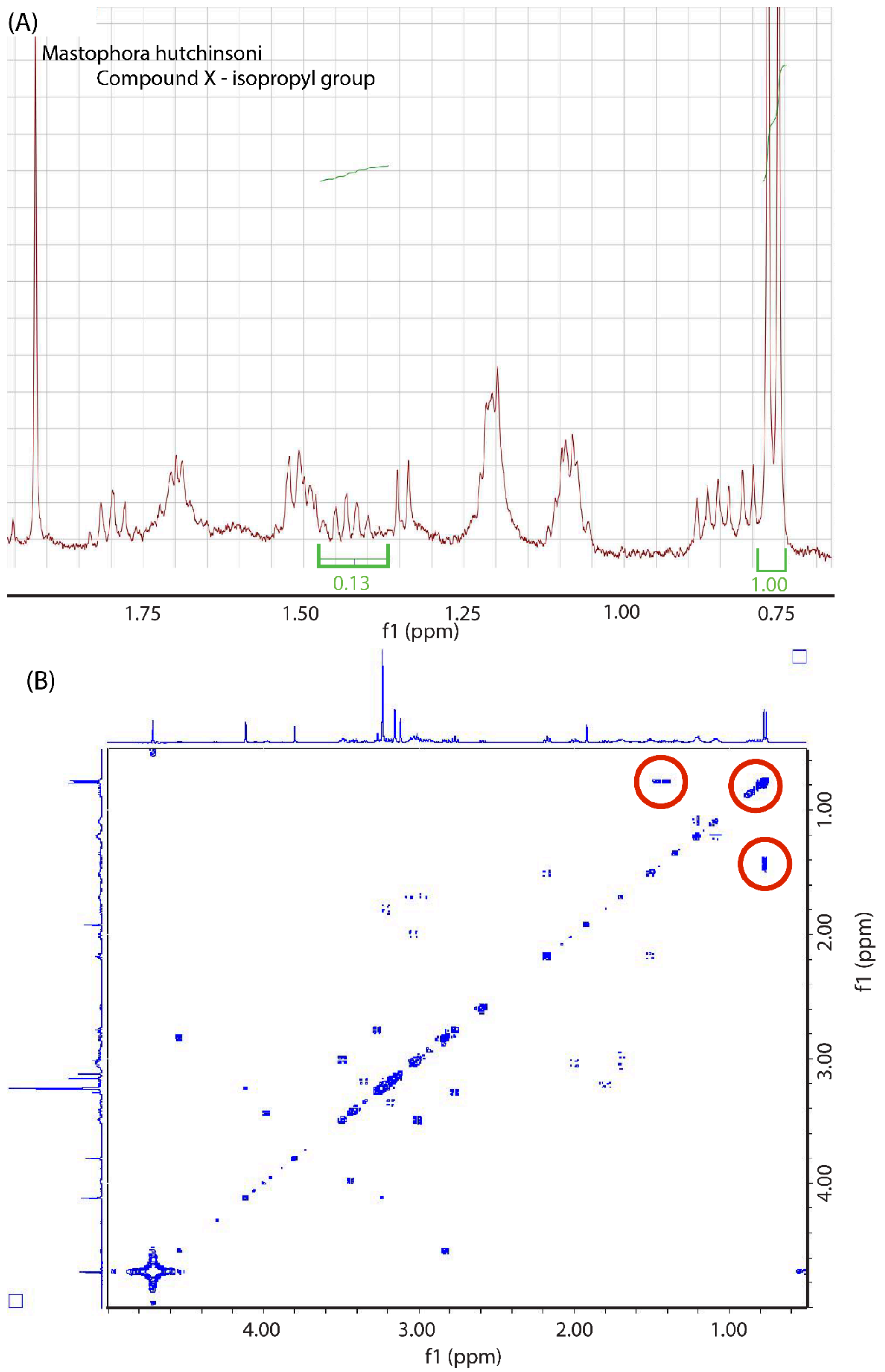

Figure S9. Identification of an isopropyl splitting/coupling pattern observed in the aggregate glue of *M. hutchinsoni*. (A) ^{1}H Solution State 400 MHz NMR spectrum of water soluble LMMCs dissolved in D_2O from the aggregate glue of *M. hutchinsoni* displaying the relative integrations of the coupled doublet and septet peaks located at 0.8 ppm and 1.5 ppm, respectively. (B) ^{1}H–^{1}H NMR Correlation Spectroscopy 2D NMR spectrum of water soluble LMMCs dissolved in D_2O from the aggregate glue of *M. hutchinsoni.*
