# Supplementary figures and images for: Special Prey, Special Glue: NMR Spectroscopy on Aggregate Glue Components of Moth-Specialist Spiders, Cyrtarachninae
Source: Biomimetics (Basel). 2024 Apr 23;9(5):256. doi: 10.3390/biomimetics9050256 (PMC11117802; doi:10.3390/biomimetics9050256)

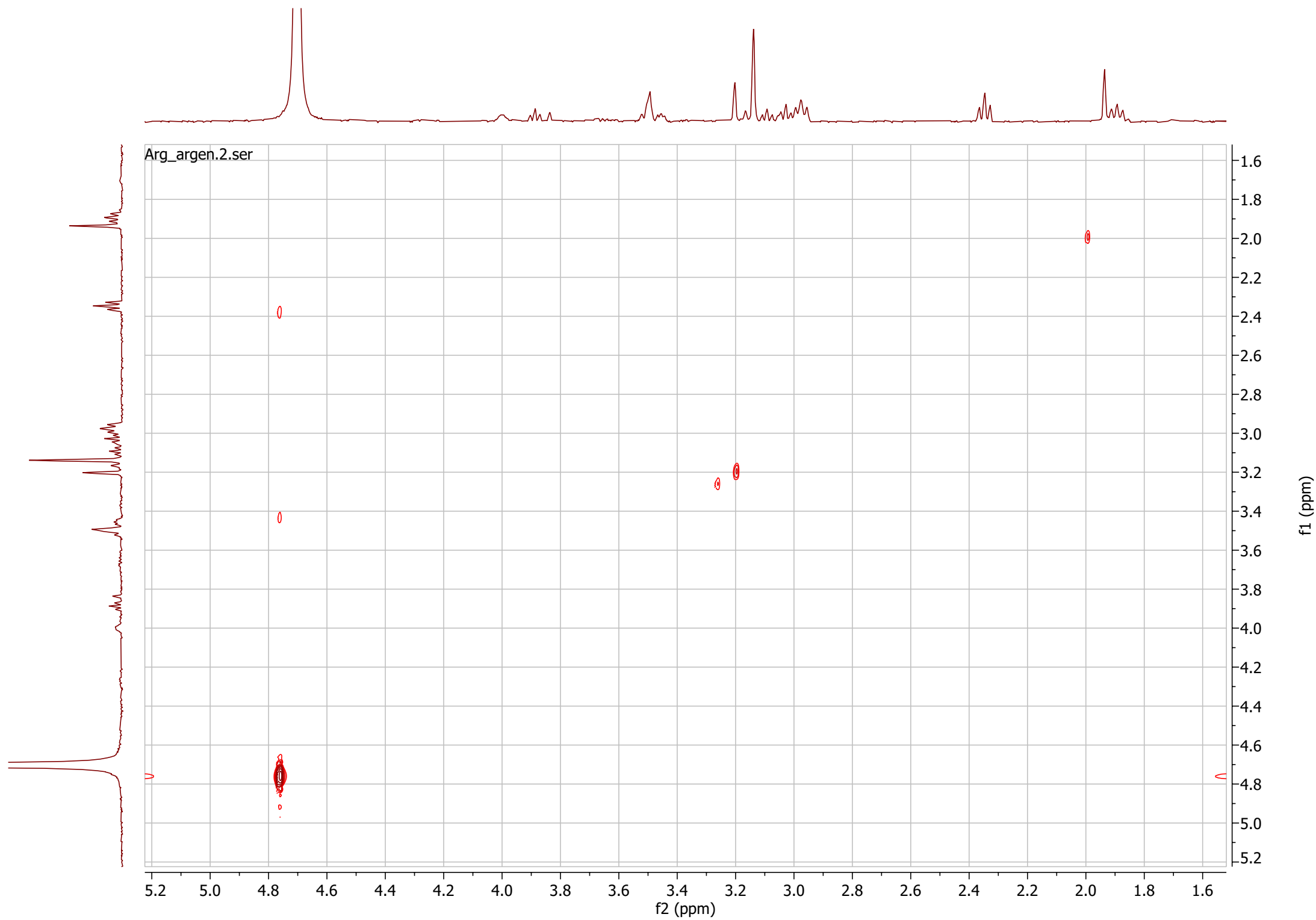

Supplement: Supplementary file 1 [file biomimetics-09-00256-s001.zip › Raw NMR Data/A_argentata/Arg_arg_cosy.pdf]

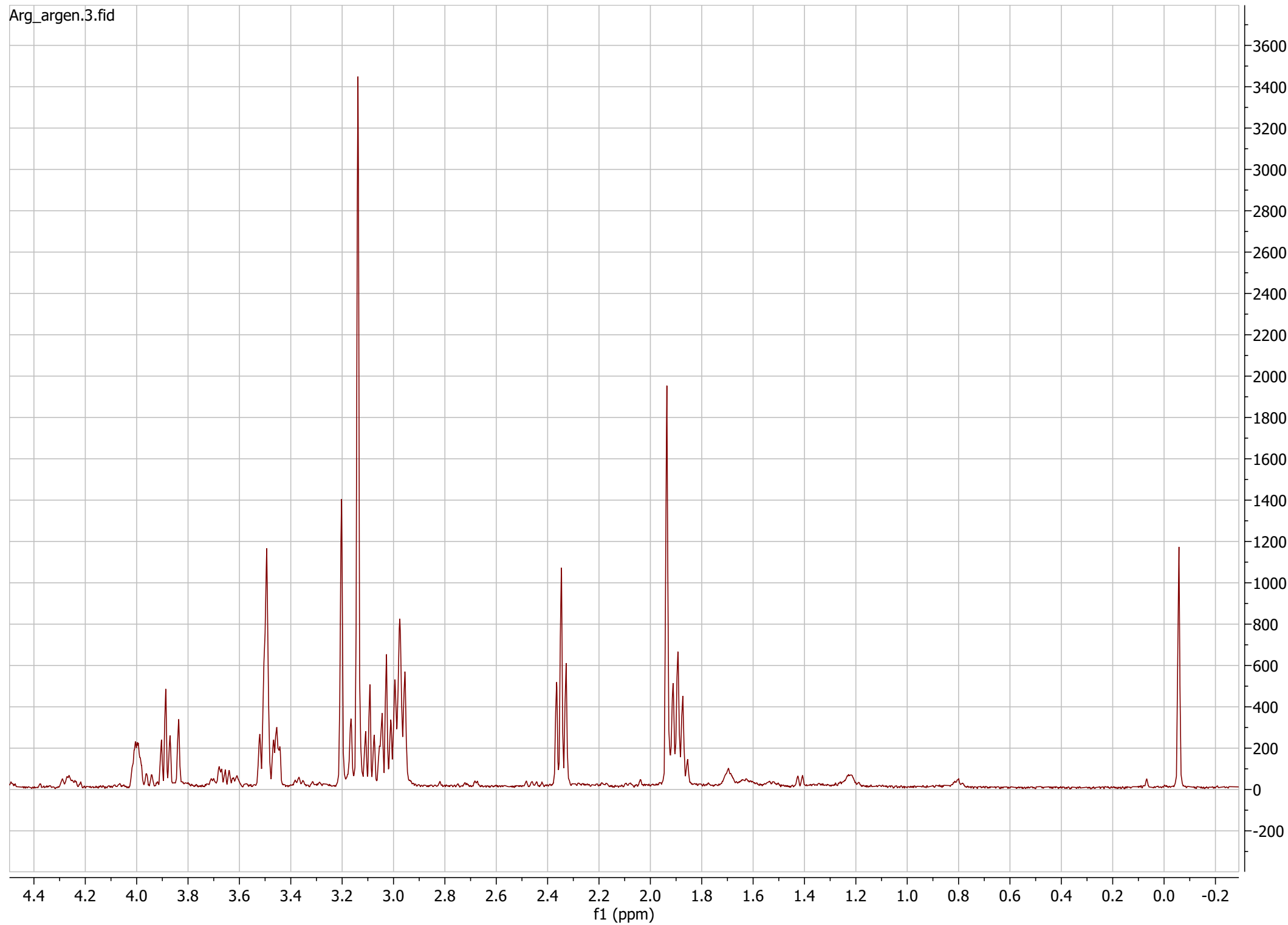

Supplement: Supplementary file 1 [file biomimetics-09-00256-s001.zip › Raw NMR Data/A_argentata/Arg_arg_proton.pdf]

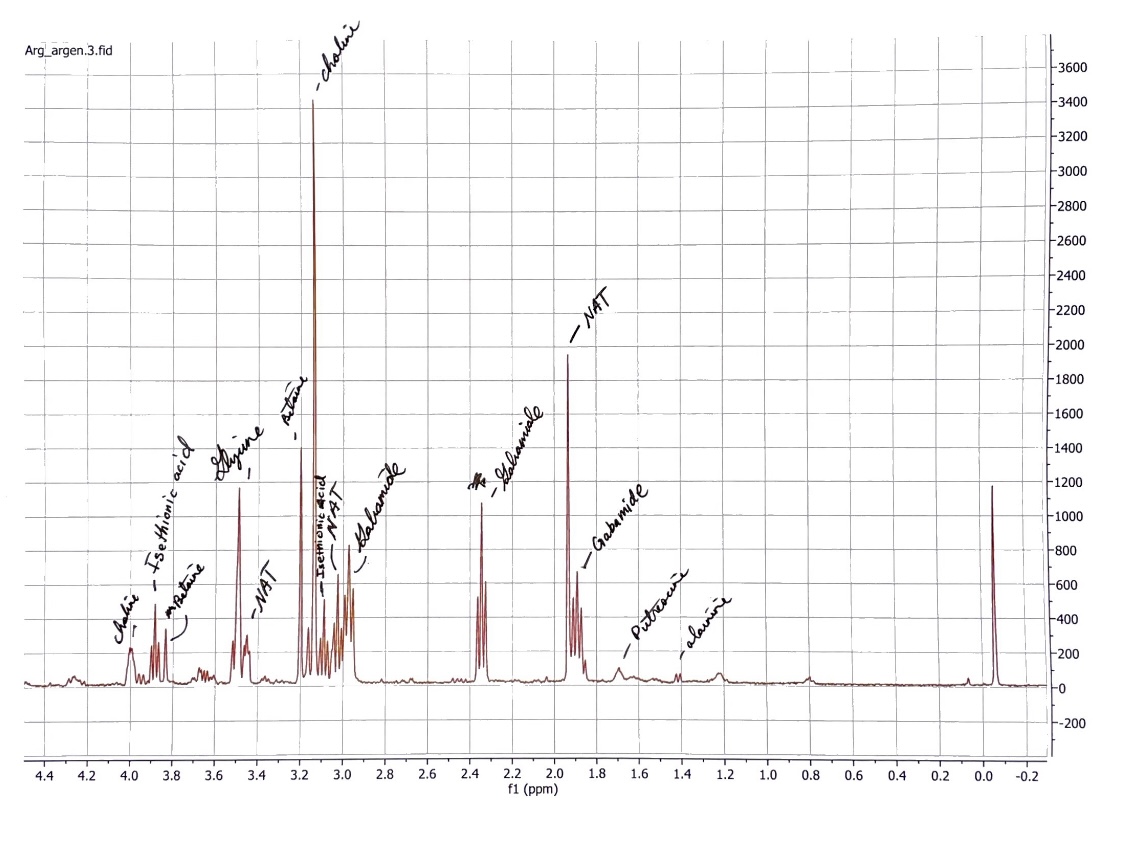

Supplement: Supplementary file 1 [file biomimetics-09-00256-s001.zip › Raw NMR Data/A_argentata/Arg_Argen_MaxAnnotated.jpg]

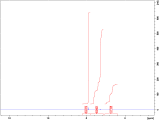

Supplement: Supplementary file 1 [file biomimetics-09-00256-s001.zip › Raw NMR Data/A_trifasicata/1/pdata/1/thumb.png]

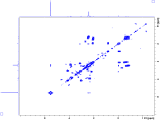

Supplement: Supplementary file 1 [file biomimetics-09-00256-s001.zip › Raw NMR Data/A_trifasicata/2/pdata/1/thumb.png]

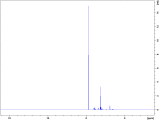

Supplement: Supplementary file 1 [file biomimetics-09-00256-s001.zip › Raw NMR Data/A_trifasicata/3/pdata/1/thumb.png]

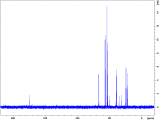

Supplement: Supplementary file 1 [file biomimetics-09-00256-s001.zip › Raw NMR Data/A_trifasicata/4/pdata/1/thumb.png]

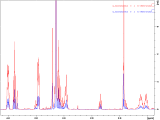

Supplement: Supplementary file 1 [file biomimetics-09-00256-s001.zip › Raw NMR Data/A_trifasicata/5/pdata/1/thumb.png]
