# Supplementary material for: Special Prey, Special Glue: NMR Spectroscopy on Aggregate Glue Components of Moth-Specialist Spiders, Cyrtarachninae
Source: Biomimetics (Basel). 2024 Apr 23;9(5):256. doi: 10.3390/biomimetics9050256 (PMC11117802; doi:10.3390/biomimetics9050256)
Supplement: Supplementary file 1 [file biomimetics-09-00256-s001.zip › Raw NMR Data/L_cornutus/1H NMR - assignments - L_cornutus - Web Glue.pdf]

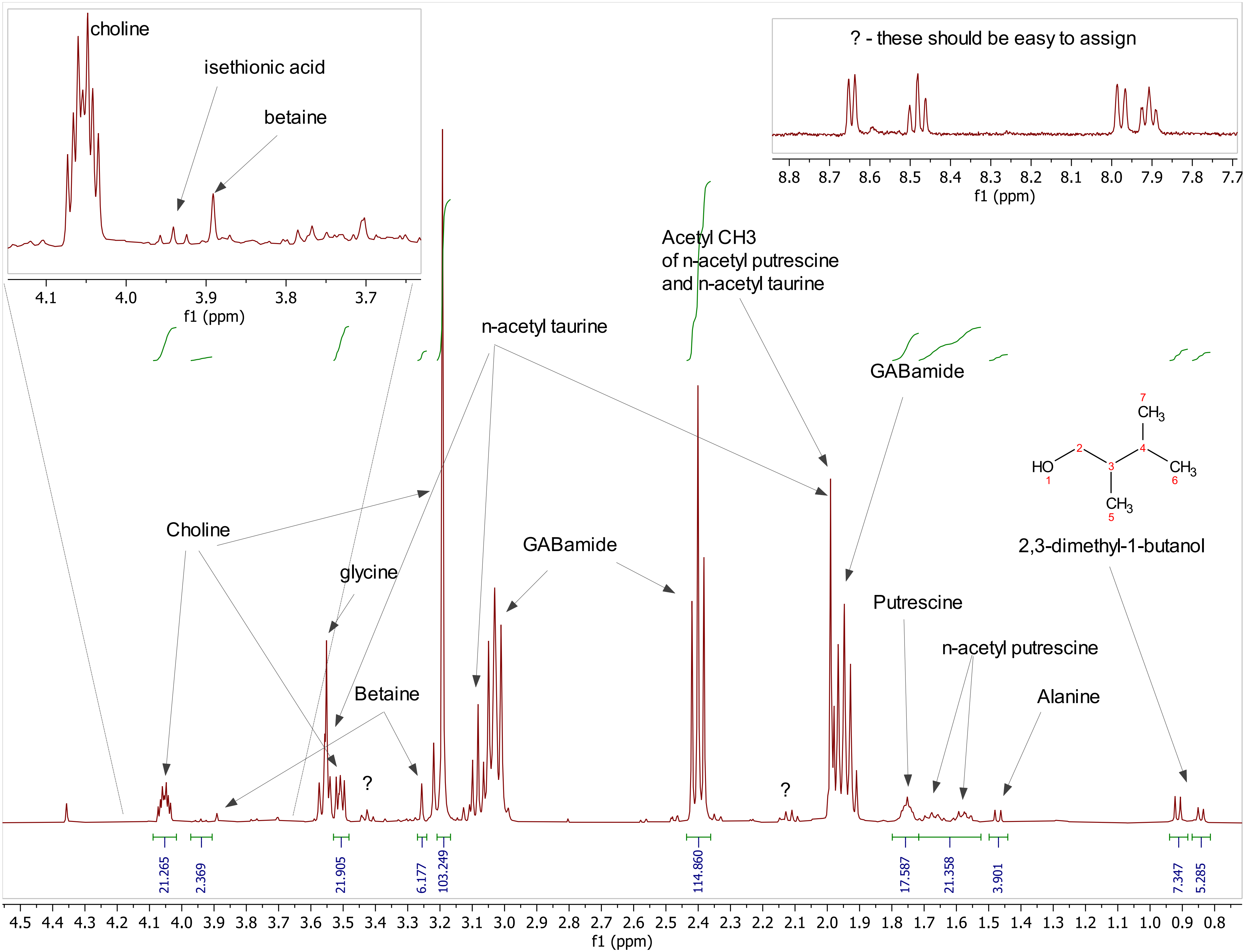

choline
isethionic acid
betaine
4.1 4.0 3.9 3.8 3.7
f1 (ppm)
? - these should be easy to assign
8.8 8.7 8.6 8.5 8.4 8.3 8.2 8.1 8.0 7.9 7.8 7.7
f1 (ppm)
Acetyl CH3
of n-acetyl putrescine
and n-acetyl taurine
n-acetyl taurine
GABamide
Choline
glycine
GABamide
Betaine
?
?
Putrescine
n-acetyl putrescine
Alanine
2,3-dimethyl-1-butanol
HO
CH3
CH3
CH3
1 2 3 4 5 6 7
21.265
2.369
21.905
6.177
103.249
114.860
17.587
21.358
3.901
7.347
5.285
4.5 4.4 4.3 4.2 4.1 4.0 3.9 3.8 3.7 3.6 3.5 3.4 3.3 3.2 3.1 3.0 2.9 2.8 2.7 2.6 2.5 2.4 2.3 2.2 2.1 2.0 1.9 1.8 1.7 1.6 1.5 1.4 1.3 1.2 1.1 1.0 0.9 0.8
f1 (ppm)

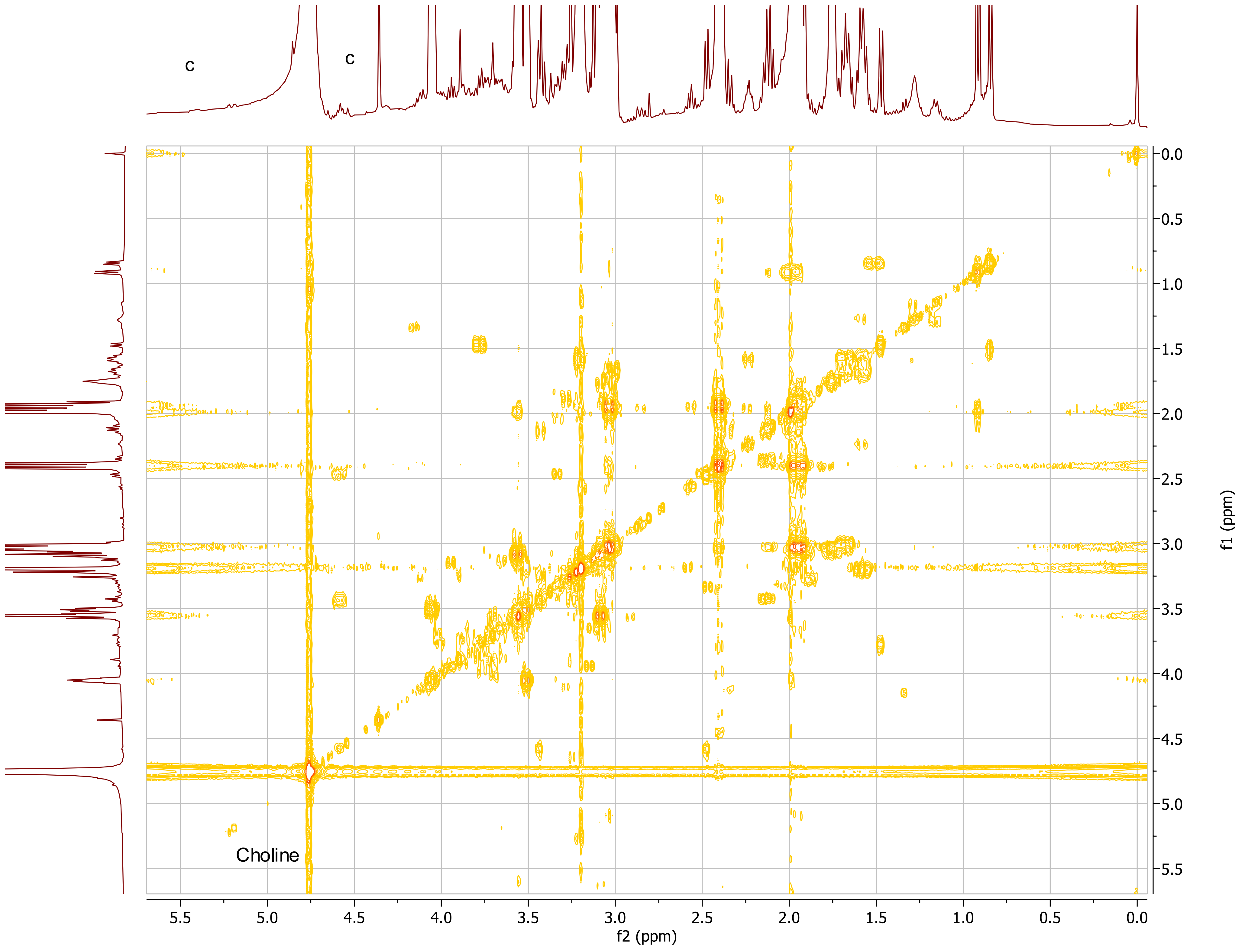
c
c
Choline
f2 (ppm)
f1 (ppm)
5.5
5.0
4.5
4.0
3.5
3.0
2.5
2.0
1.5
1.0
0.5
0.0
