# Supplementary material for: Special Prey, Special Glue: NMR Spectroscopy on Aggregate Glue Components of Moth-Specialist Spiders, Cyrtarachninae
Source: Biomimetics (Basel). 2024 Apr 23;9(5):256. doi: 10.3390/biomimetics9050256 (PMC11117802; doi:10.3390/biomimetics9050256)
Supplement: Supplementary file 1 [file biomimetics-09-00256-s001.zip › Raw NMR Data/L_cornutus/L_cornutus_13C_1.pdf]

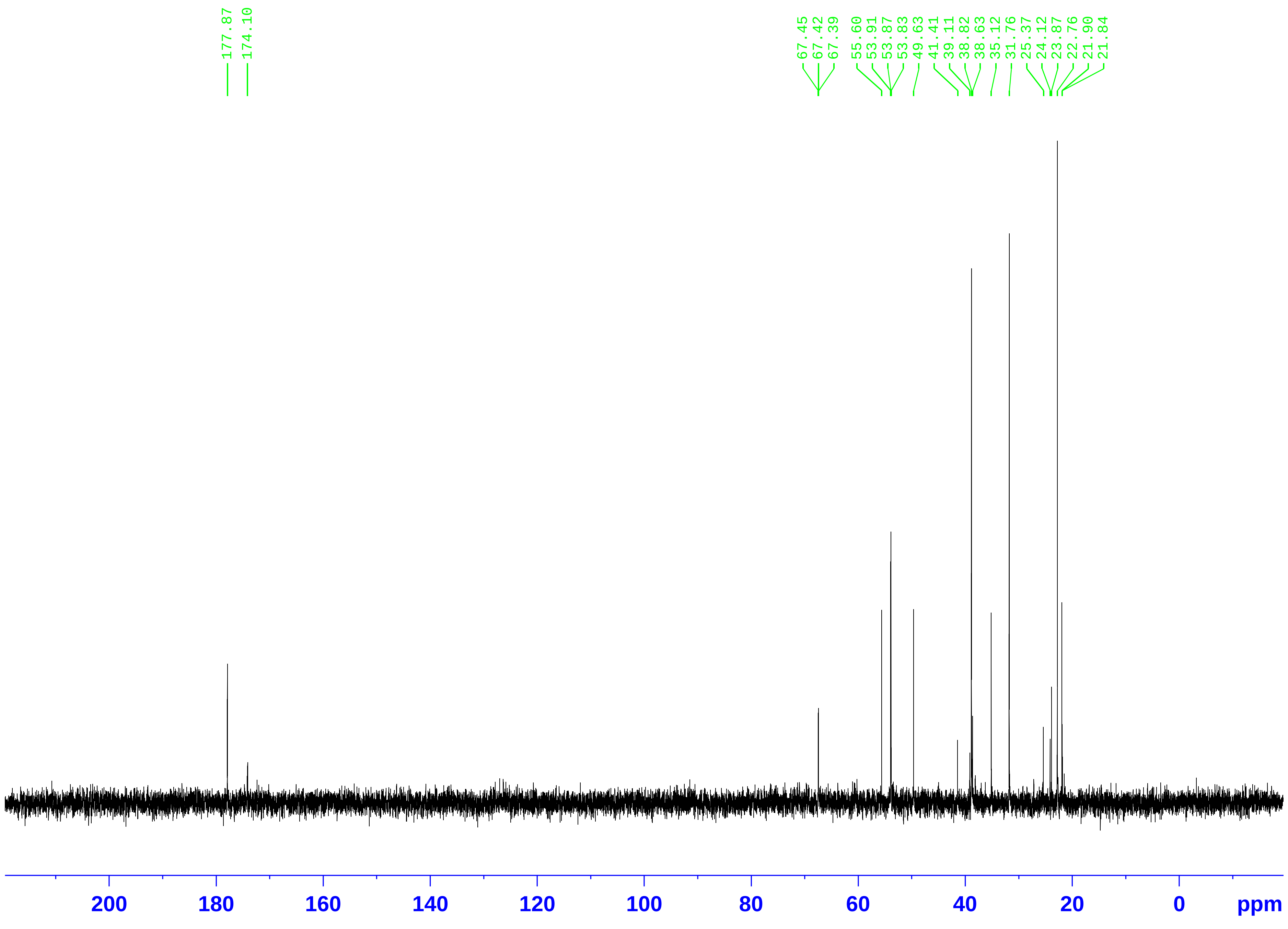
177.87
174.10
67.45
67.42
67.39
55.60
53.91
53.87
53.83
49.63
41.41
39.11
38.82
38.63
35.12
31.76
25.37
24.12
23.87
22.76
21.90
21.84
200
180
160
140
120
100
80
60
40
20
0
ppm
