# Supplementary material for: Special Prey, Special Glue: NMR Spectroscopy on Aggregate Glue Components of Moth-Specialist Spiders, Cyrtarachninae
Source: Biomimetics (Basel). 2024 Apr 23;9(5):256. doi: 10.3390/biomimetics9050256 (PMC11117802; doi:10.3390/biomimetics9050256)
Supplement: Supplementary file 1 [file biomimetics-09-00256-s001.zip › Raw NMR Data/L_cornutus/L_cornutus_cosy2.pdf]

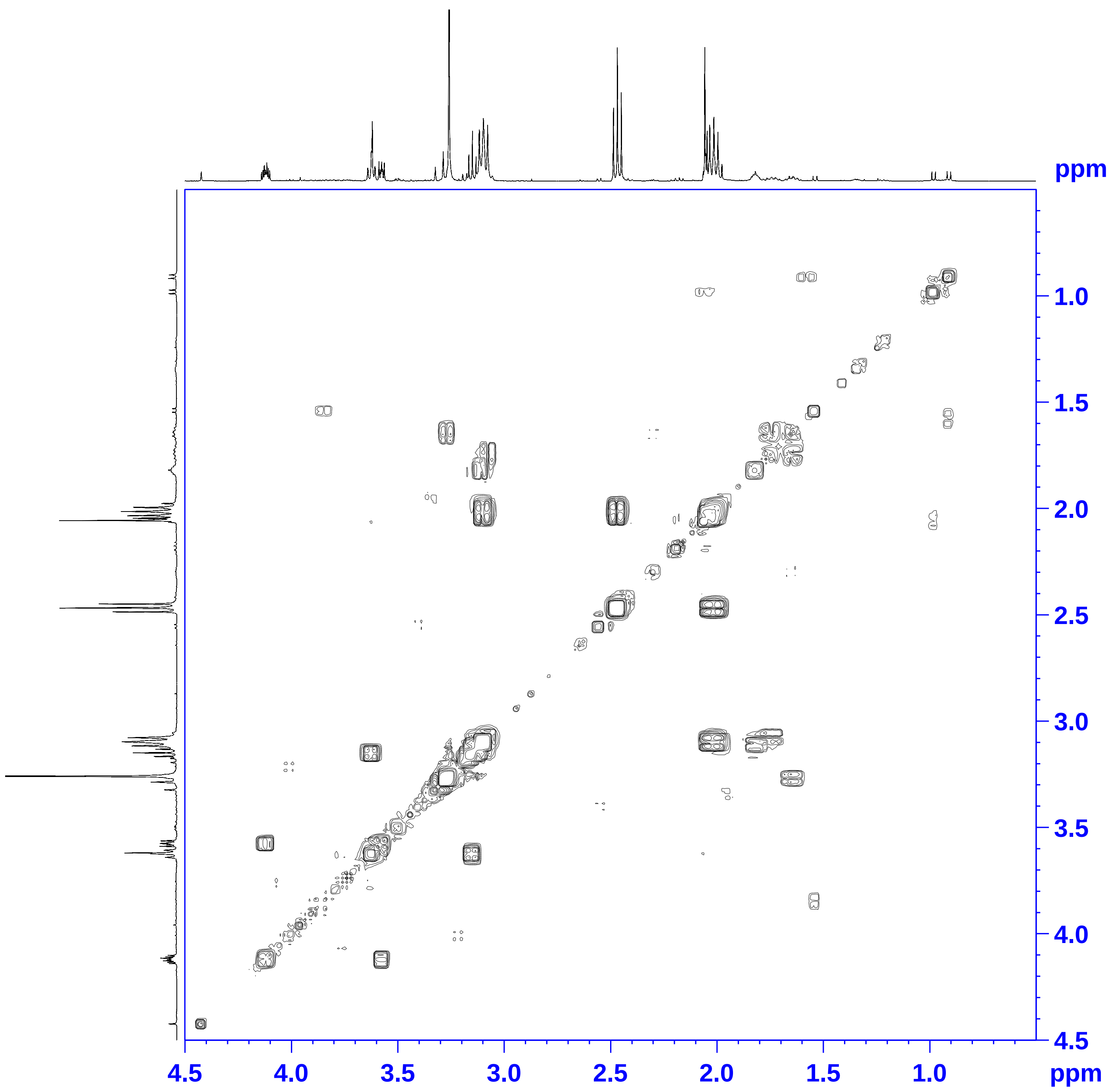
ppm
1.0
1.5
2.0
2.5
3.0
3.5
4.0
4.5
4.5
4.0
3.5
3.0
2.5
2.0
1.5
1.0
ppm
