# Supplementary material for: Special Prey, Special Glue: NMR Spectroscopy on Aggregate Glue Components of Moth-Specialist Spiders, Cyrtarachninae
Source: Biomimetics (Basel). 2024 Apr 23;9(5):256. doi: 10.3390/biomimetics9050256 (PMC11117802; doi:10.3390/biomimetics9050256)
Supplement: Supplementary file 1 [file biomimetics-09-00256-s001.zip › Raw NMR Data/T_calvipes/Tri_c_proton.pdf]

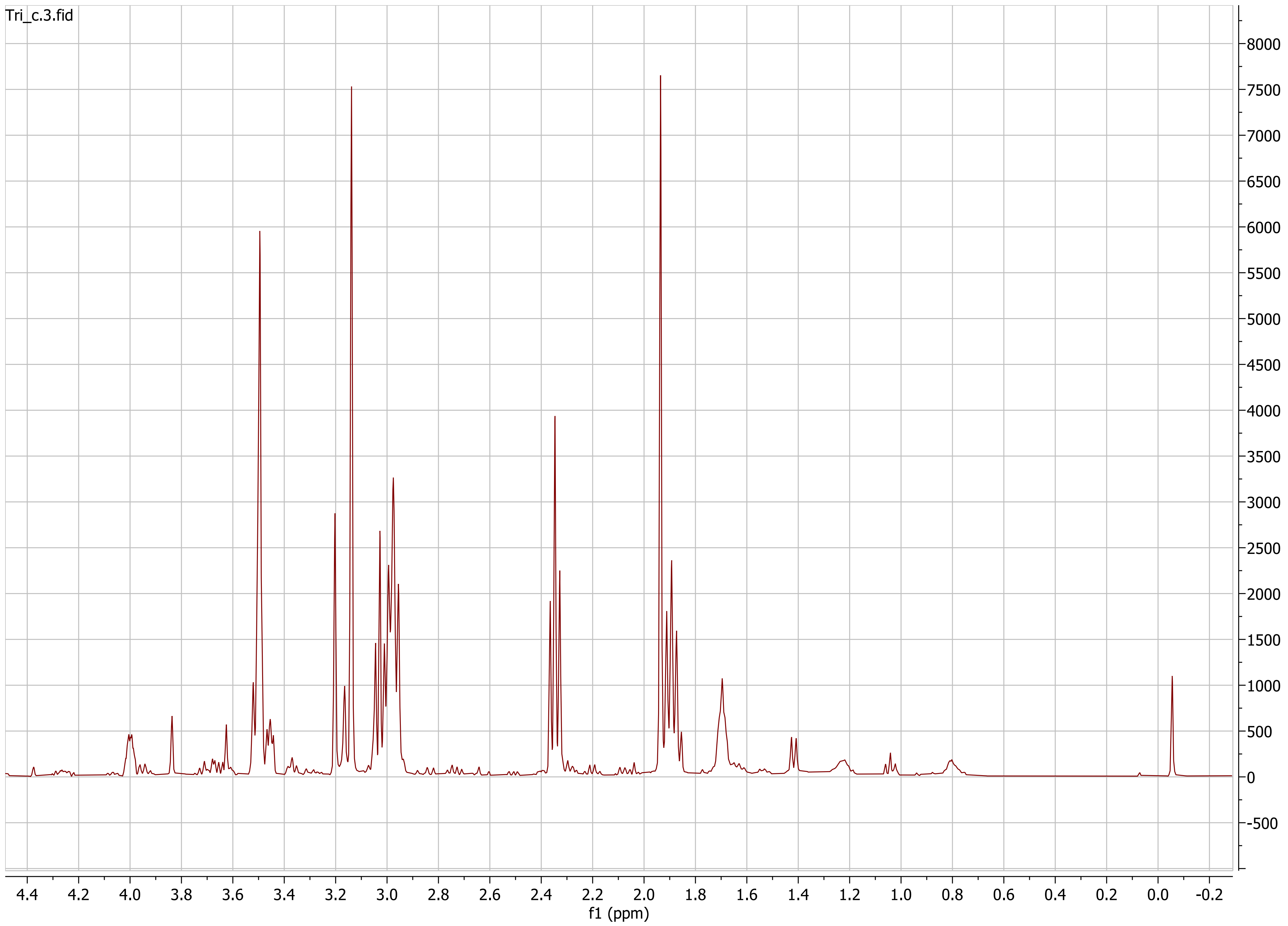

Tri_c.3.fid
8000
7500
7000
6500
6000
5500
5000
4500
4000
3500
3000
2500
2000
1500
1000
500
0
-500
4.4
4.2
4.0
3.8
3.6
3.4
3.2
3.0
2.8
2.6
2.4
2.2
2.0
1.8
1.6
1.4
1.2
1.0
0.8
0.6
0.4
0.2
0.0
-0.2
f1 (ppm)
