# Supplementary material for: Special Prey, Special Glue: NMR Spectroscopy on Aggregate Glue Components of Moth-Specialist Spiders, Cyrtarachninae
Source: Biomimetics (Basel). 2024 Apr 23;9(5):256. doi: 10.3390/biomimetics9050256 (PMC11117802; doi:10.3390/biomimetics9050256)
Supplement: Supplementary file 1 [file biomimetics-09-00256-s001.zip › Supplementary File_S2_NMR Spectra Images.docx.pdf]

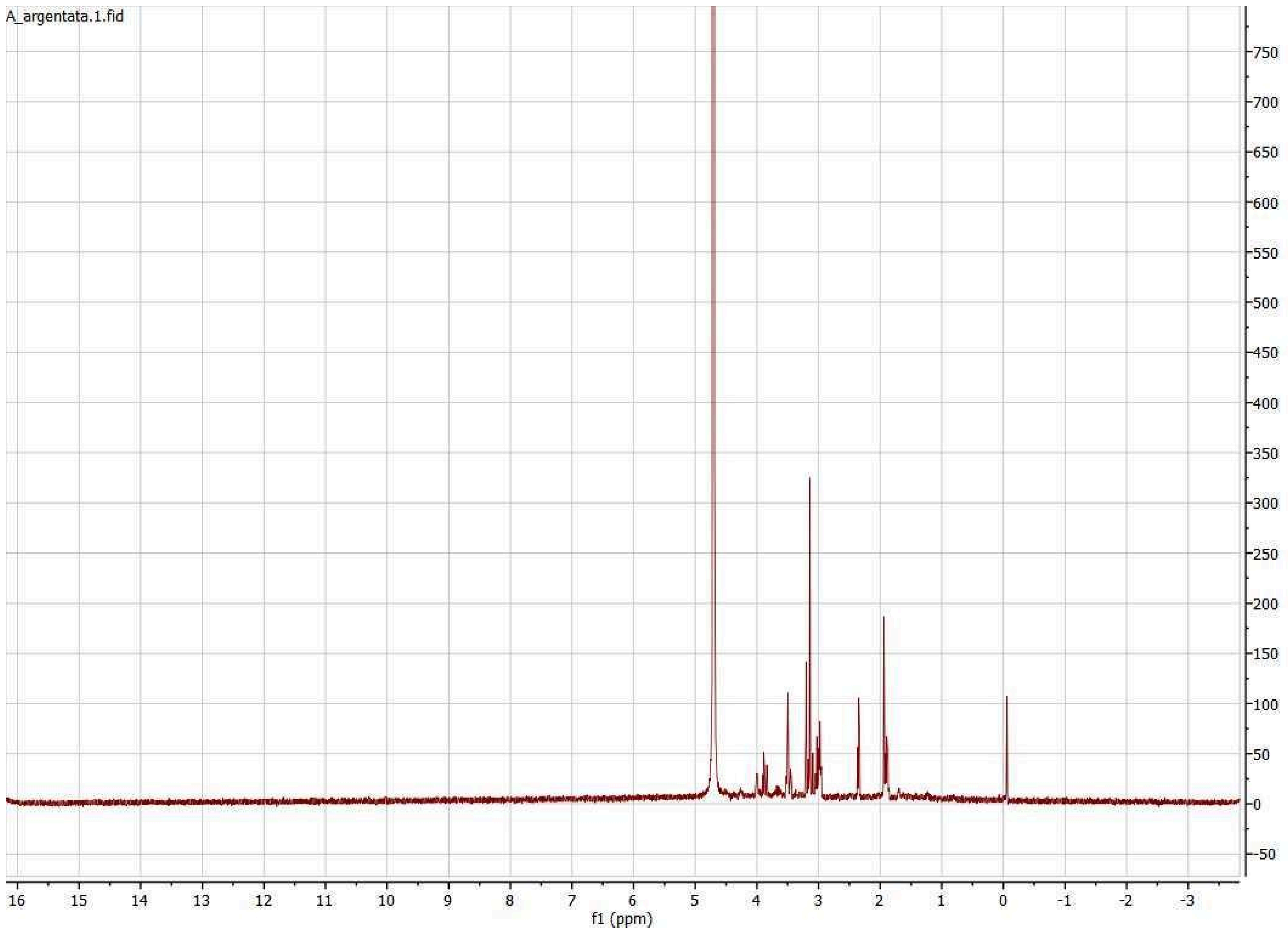

Argiope Argentata **- Survey Spectra**

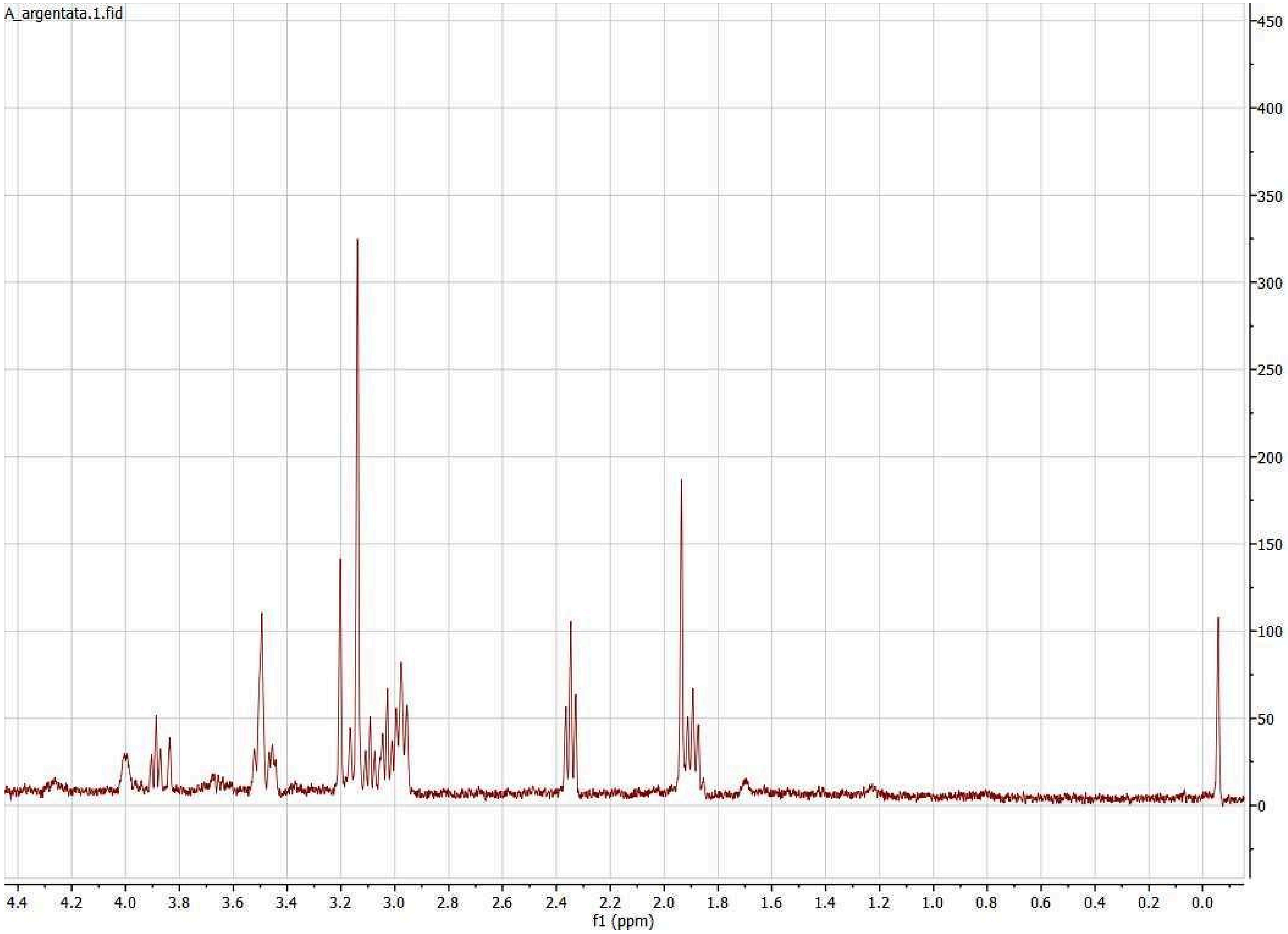

Argiope Argentata **- Extended Spectra**

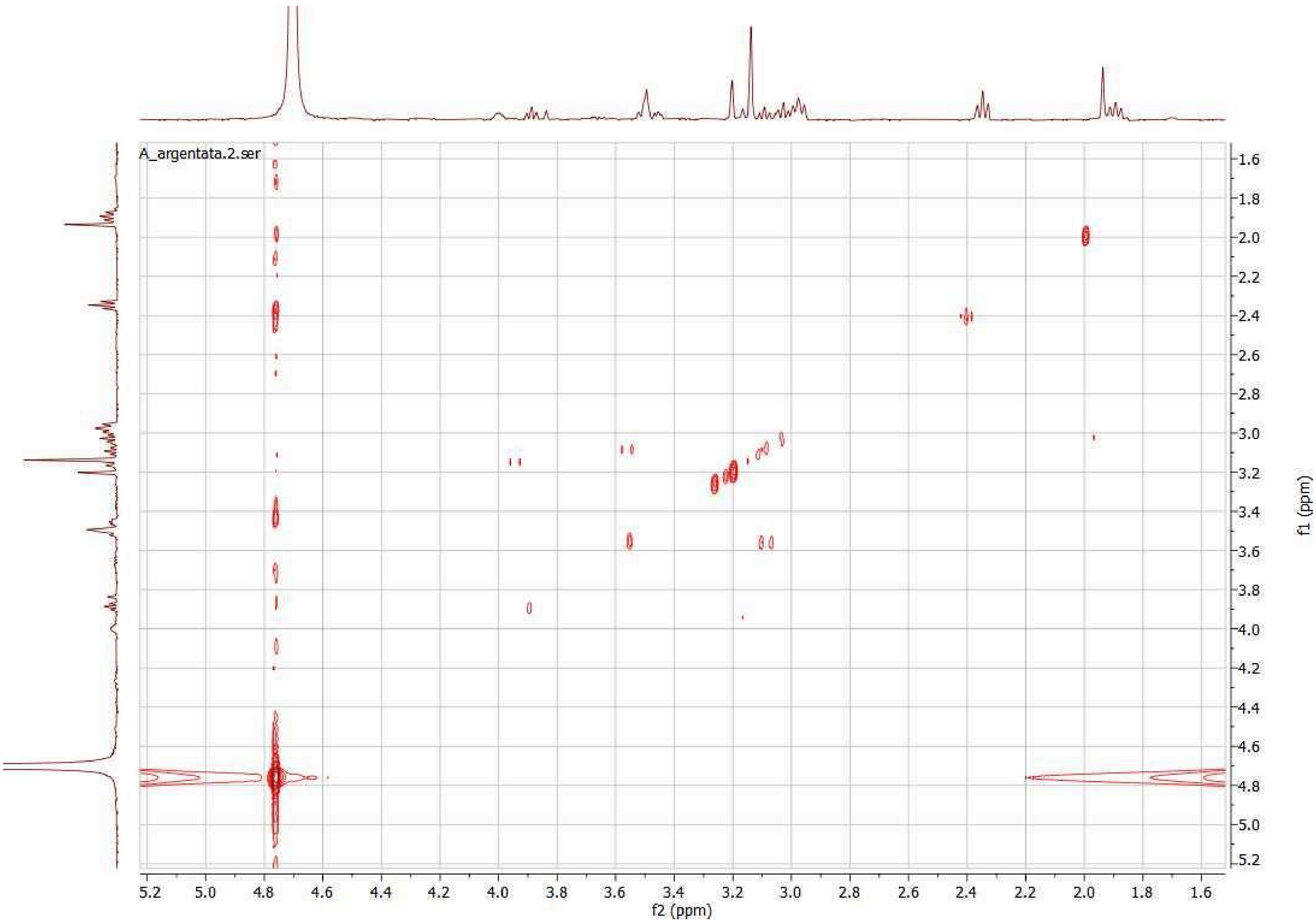

Argiope Argentata **- 2D COSY**

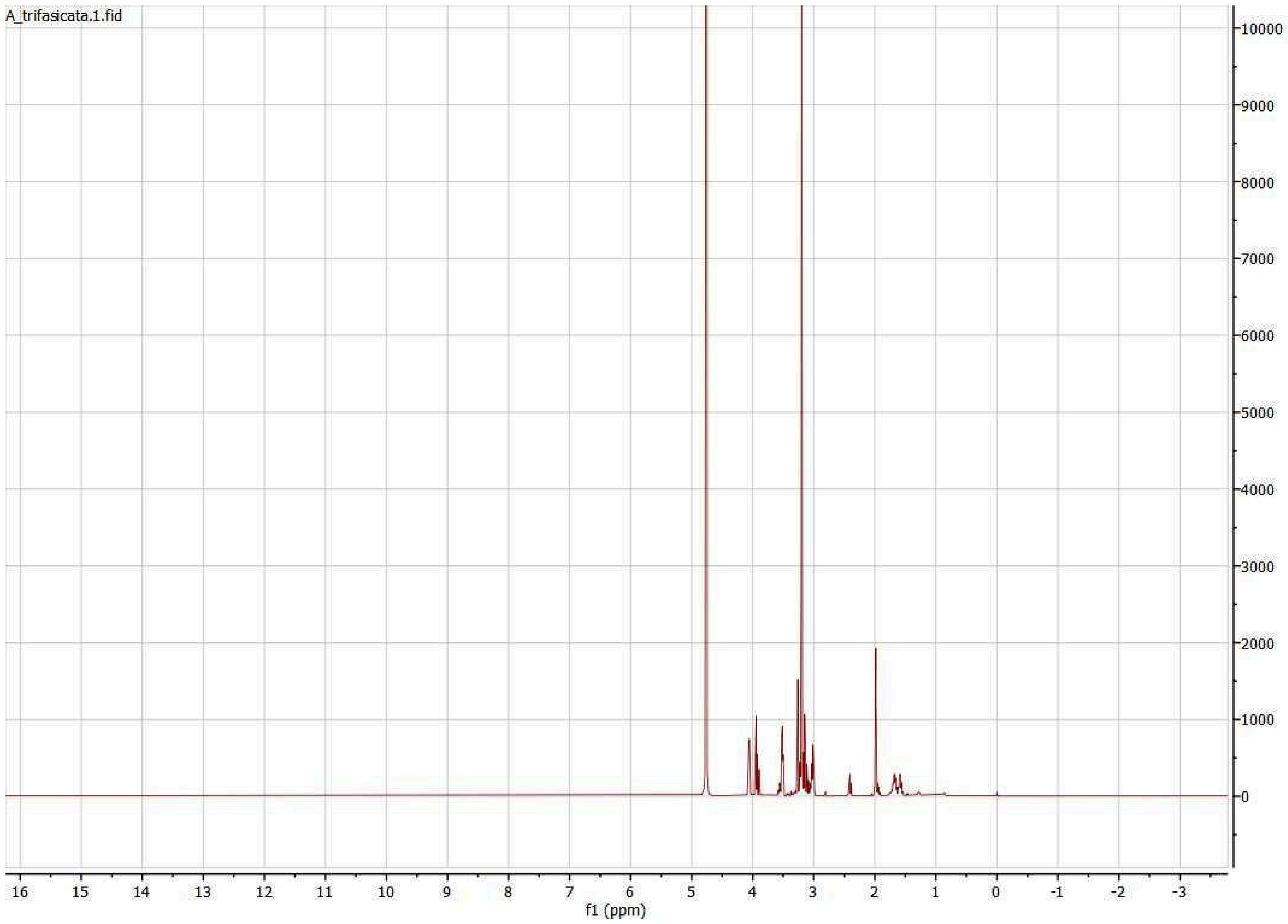
A_trifasicata.1.fid
f1 (ppm)

Argiope trifasciata - Survey Spectra

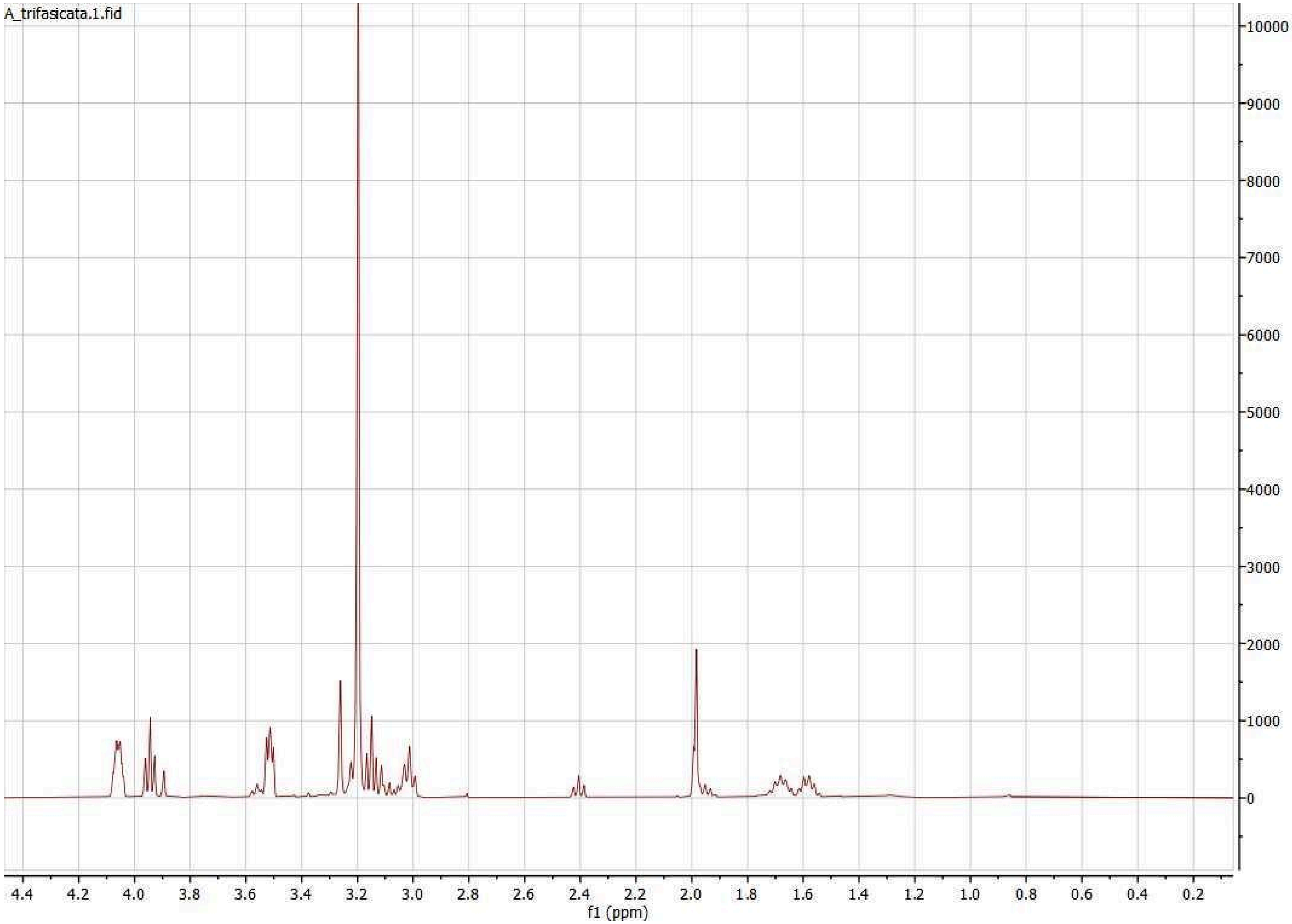

Argiope trifasciata - Extended Spectra

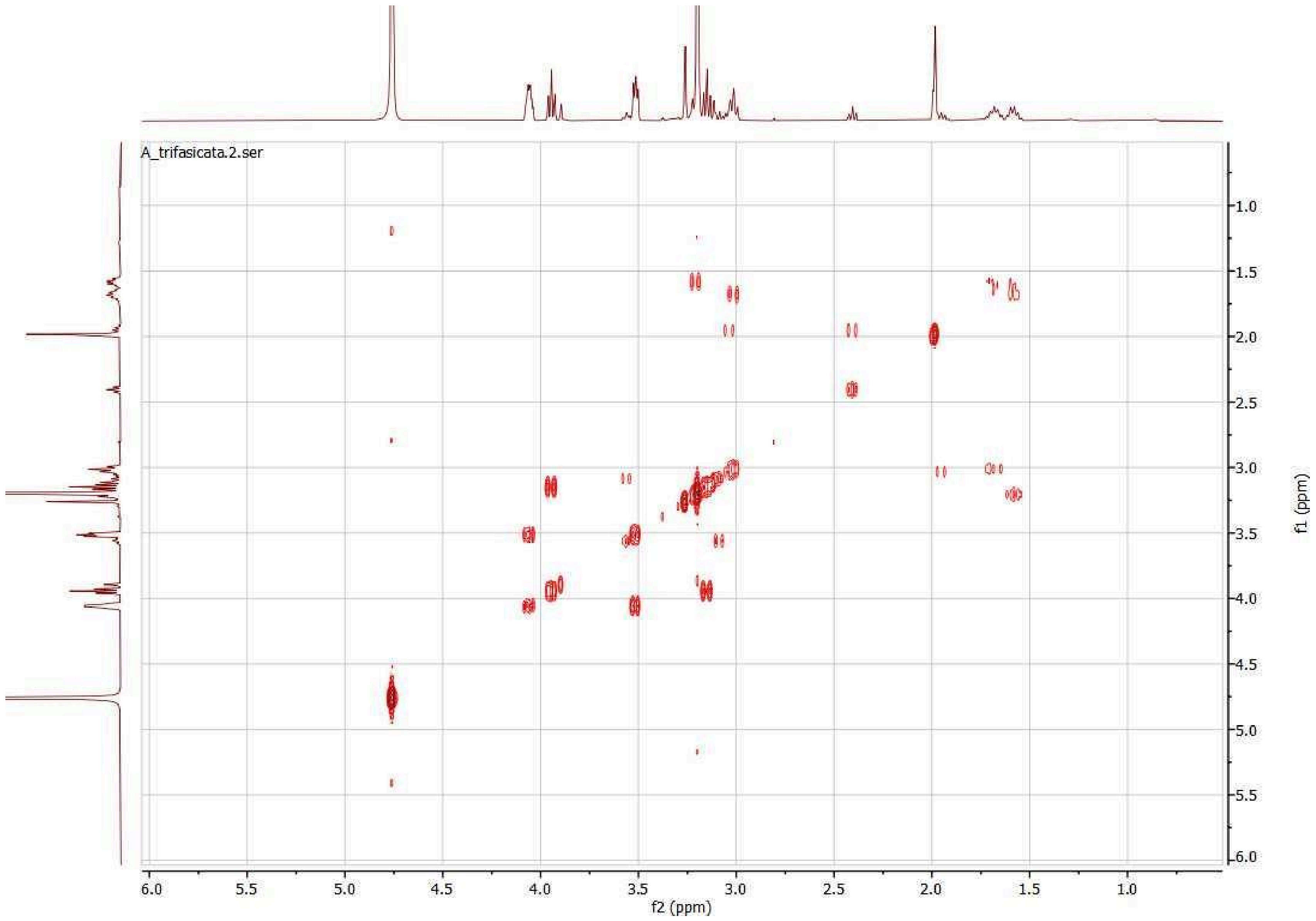
A_trifasicata.2.ser
f2 (ppm)
f1 (ppm)
6.0
5.5
5.0
4.5
4.0
3.5
3.0
2.5
2.0
1.5
1.0

***Argiope trifasciata* - 2D COSY**

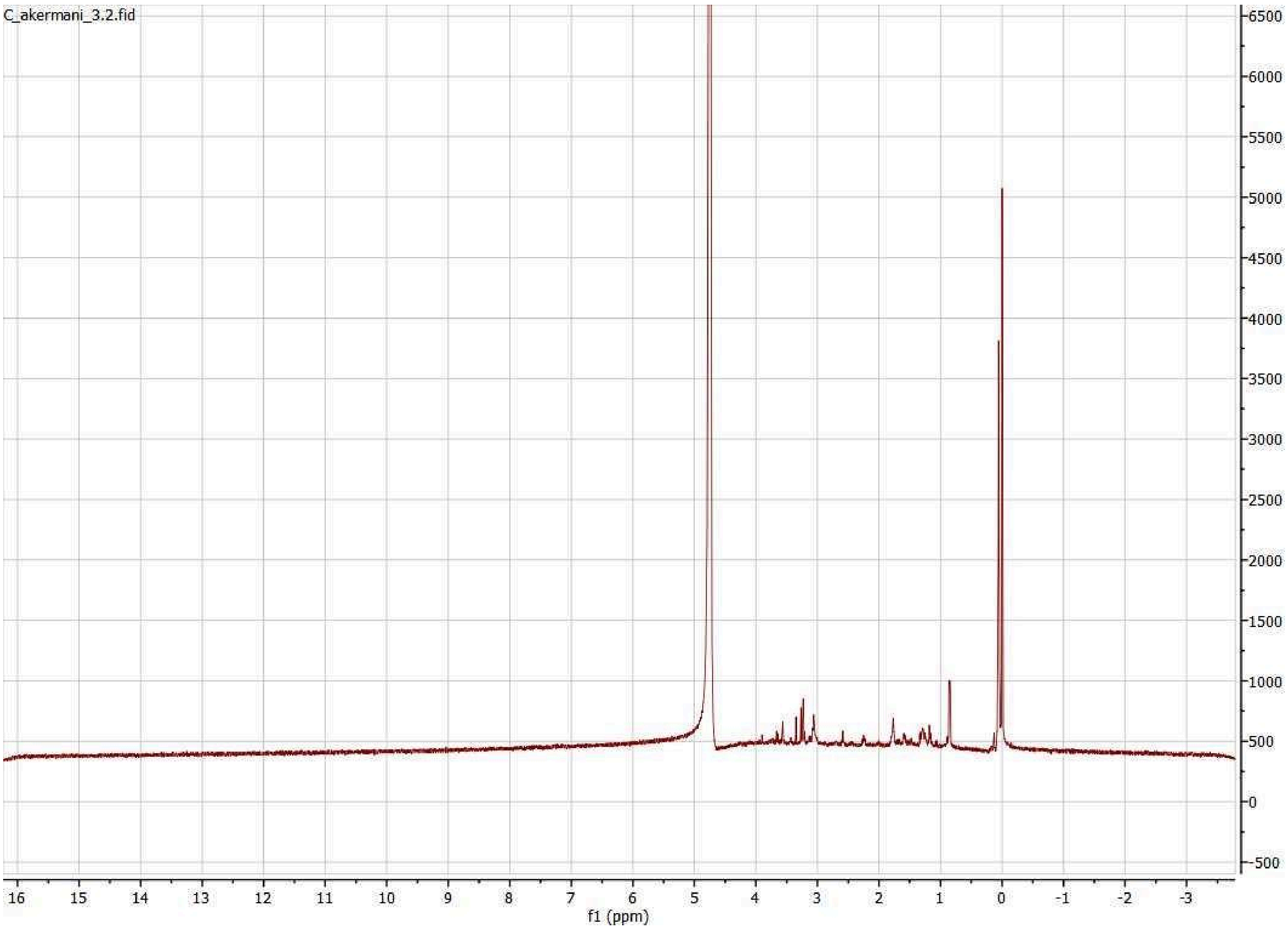

Cladomelea akermani - Survey Spectra

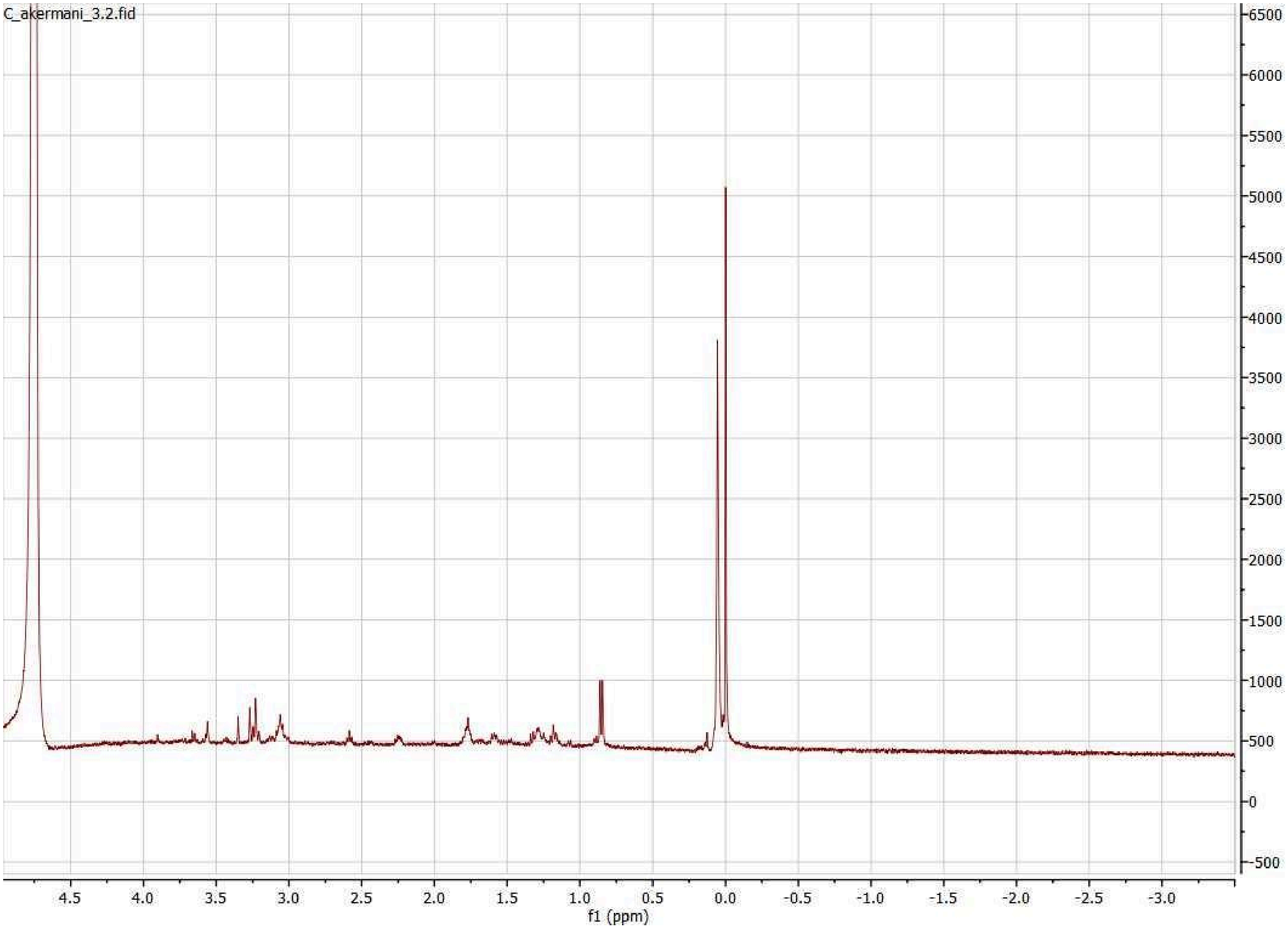

Cladomelea akermani - Extended Spectra

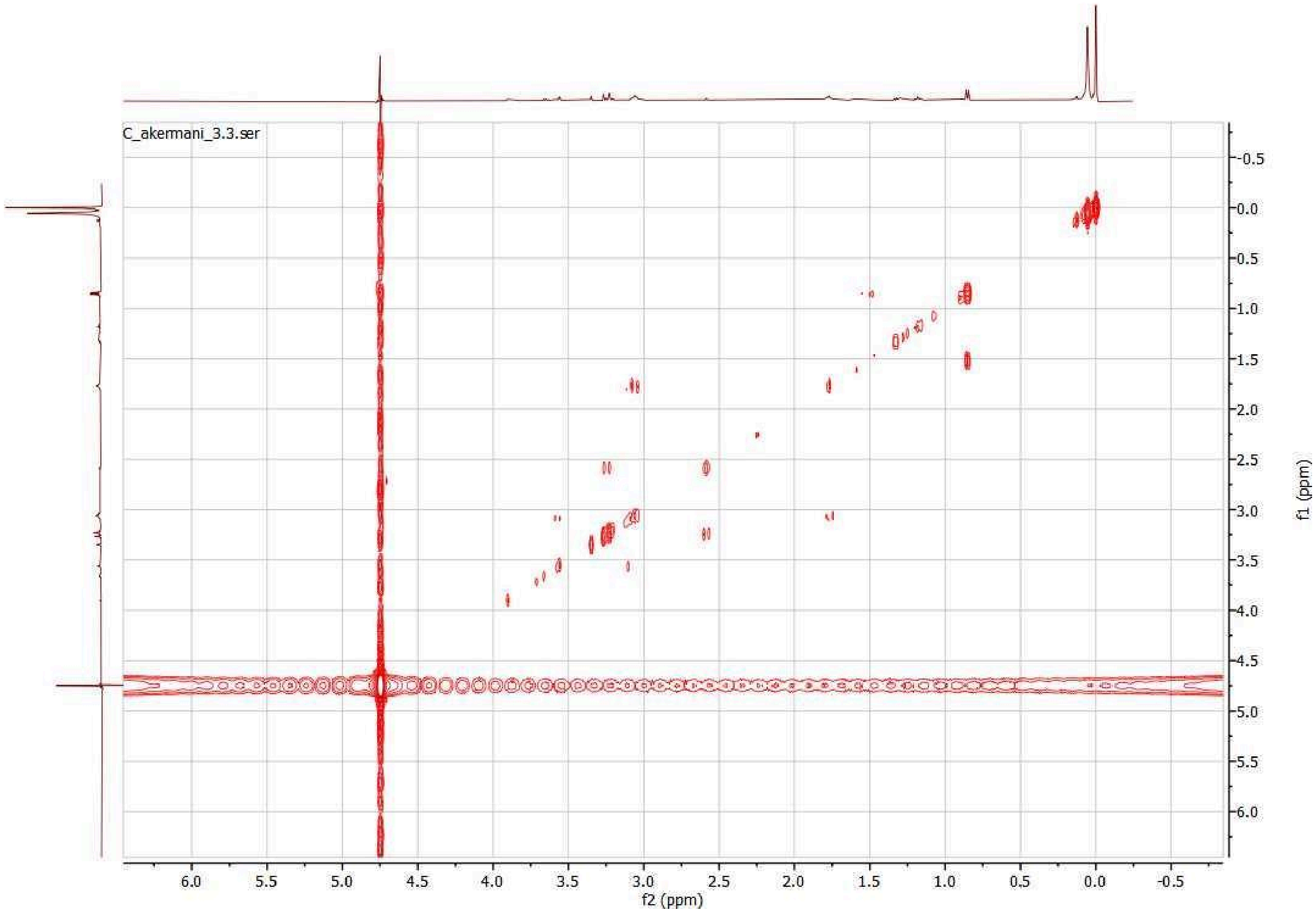

Cladomelea akermani - 2D COSY

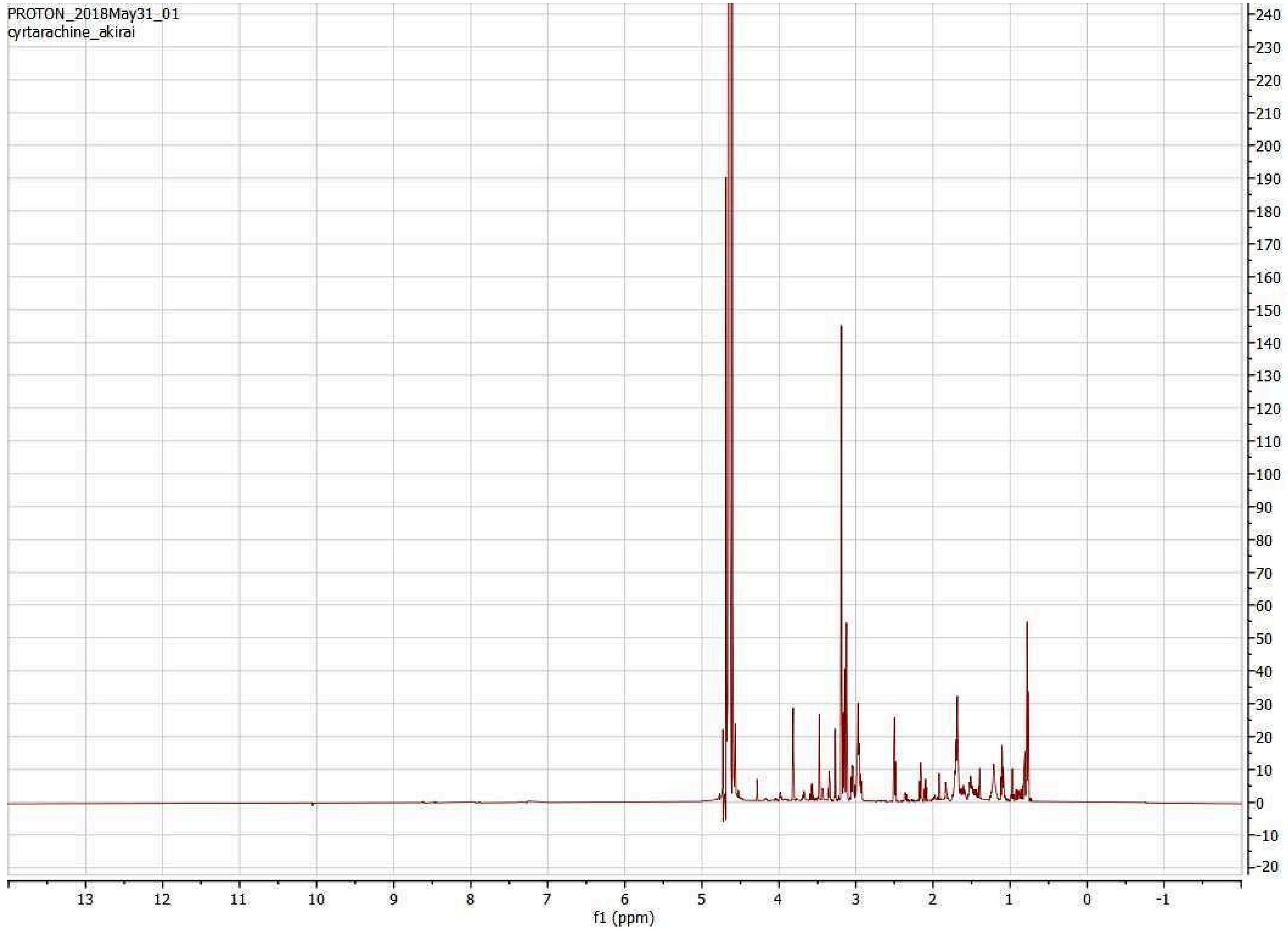

***Cyrtarachne akirai* – Survey Spectra**

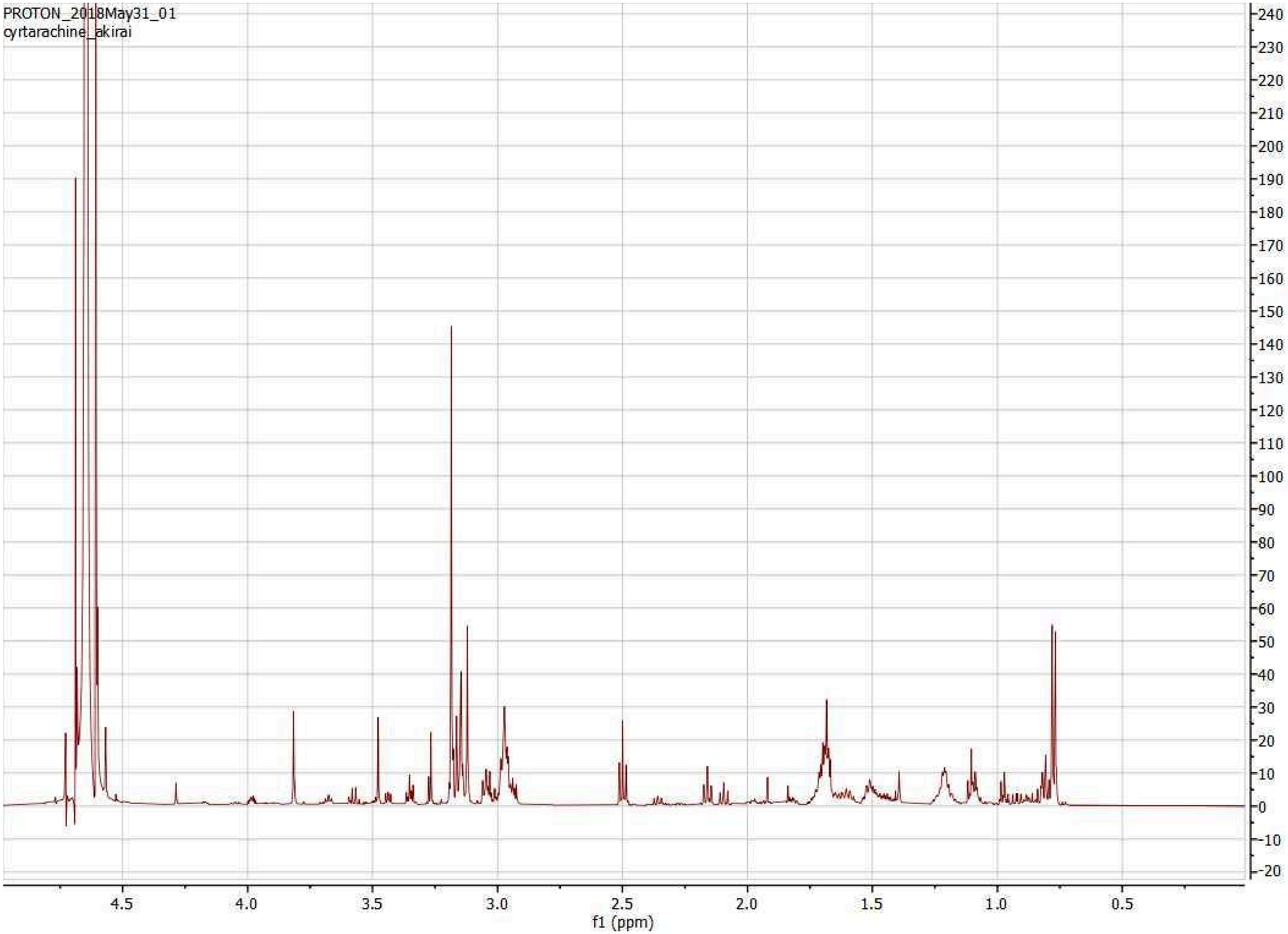

Cyrtarachne akirai – Extended Spectra

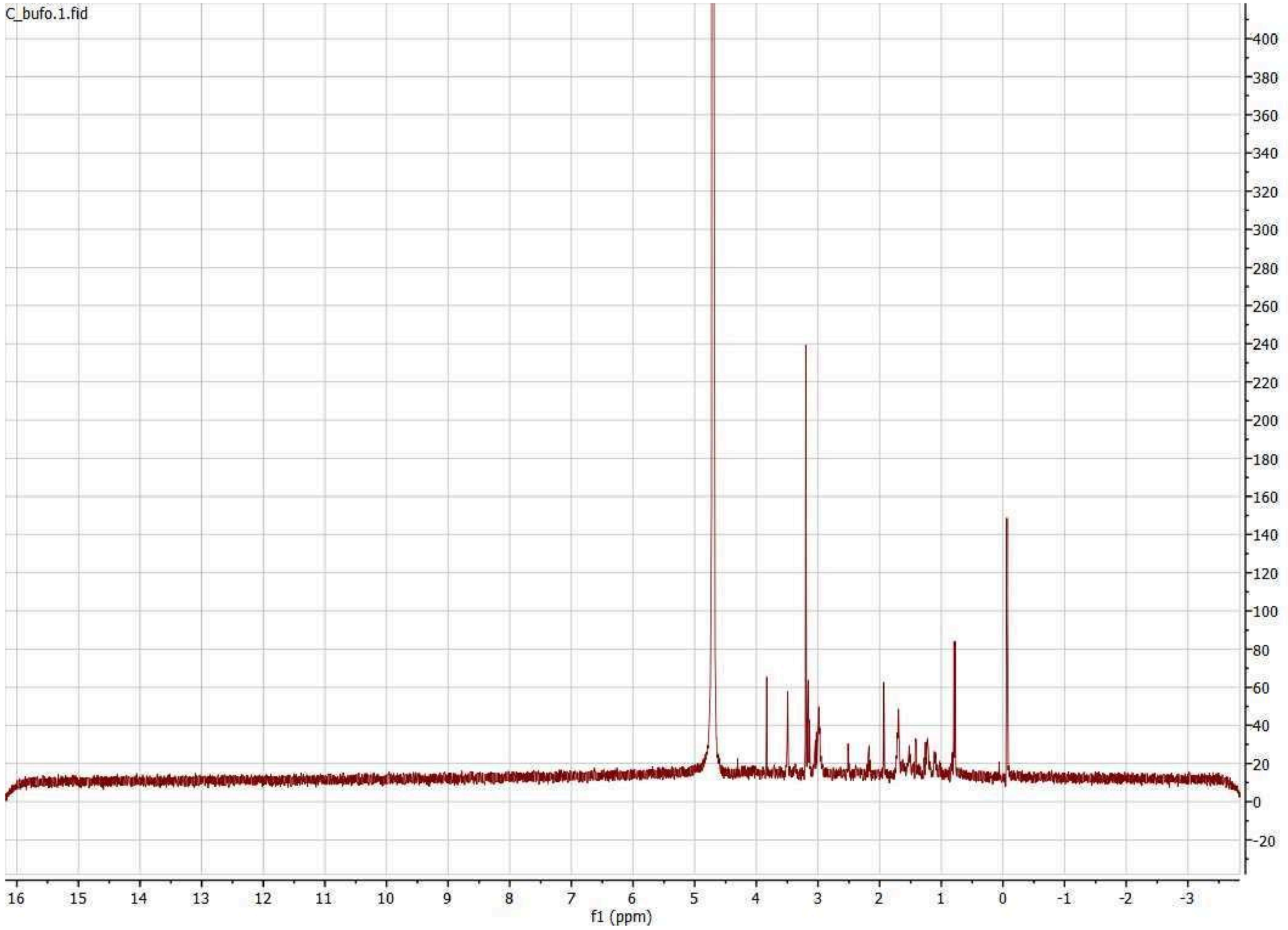

Cyrtarachne bufo – Survey Spectra

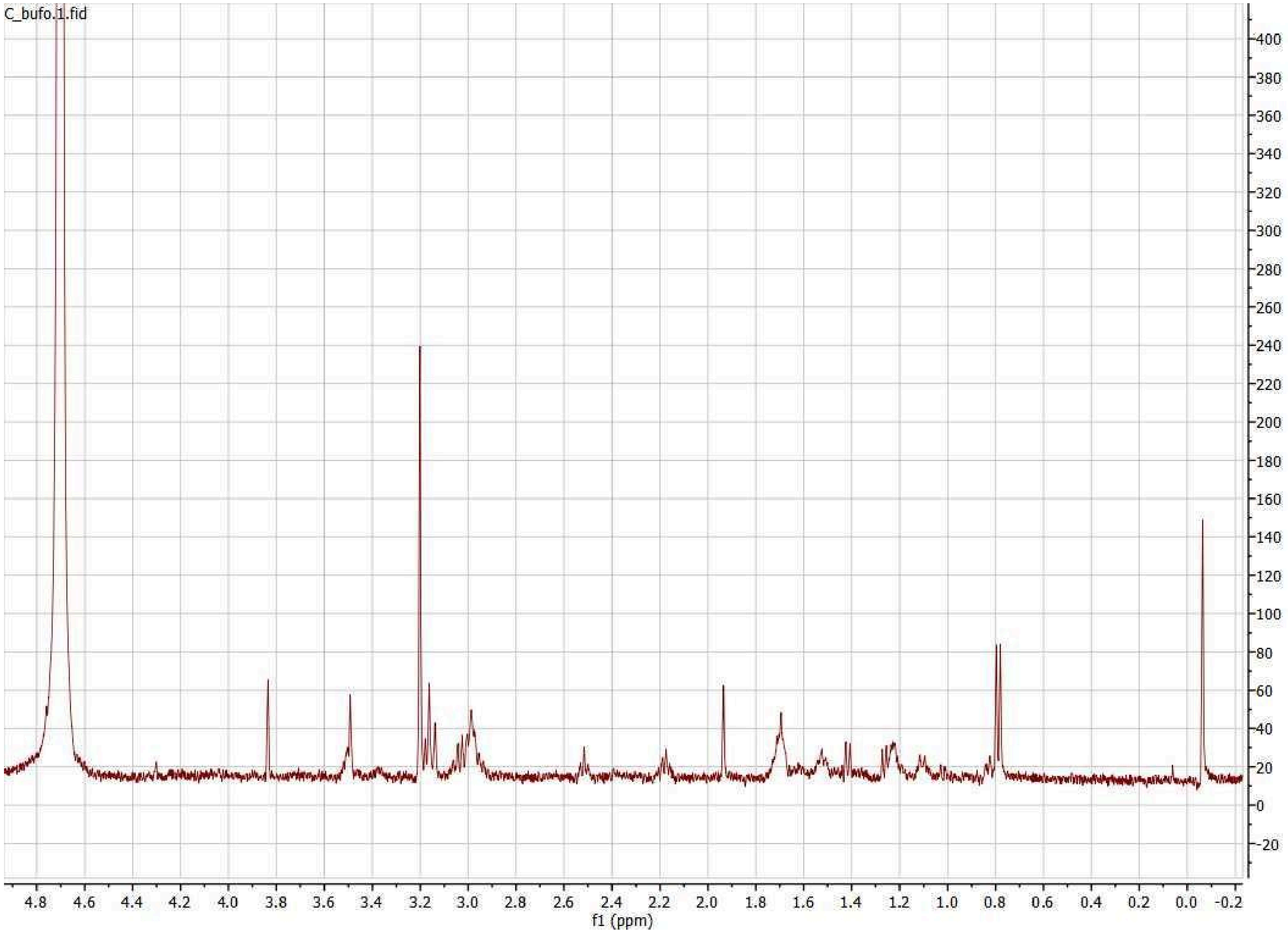

Cyrtarachne bufo – Extended Spectra

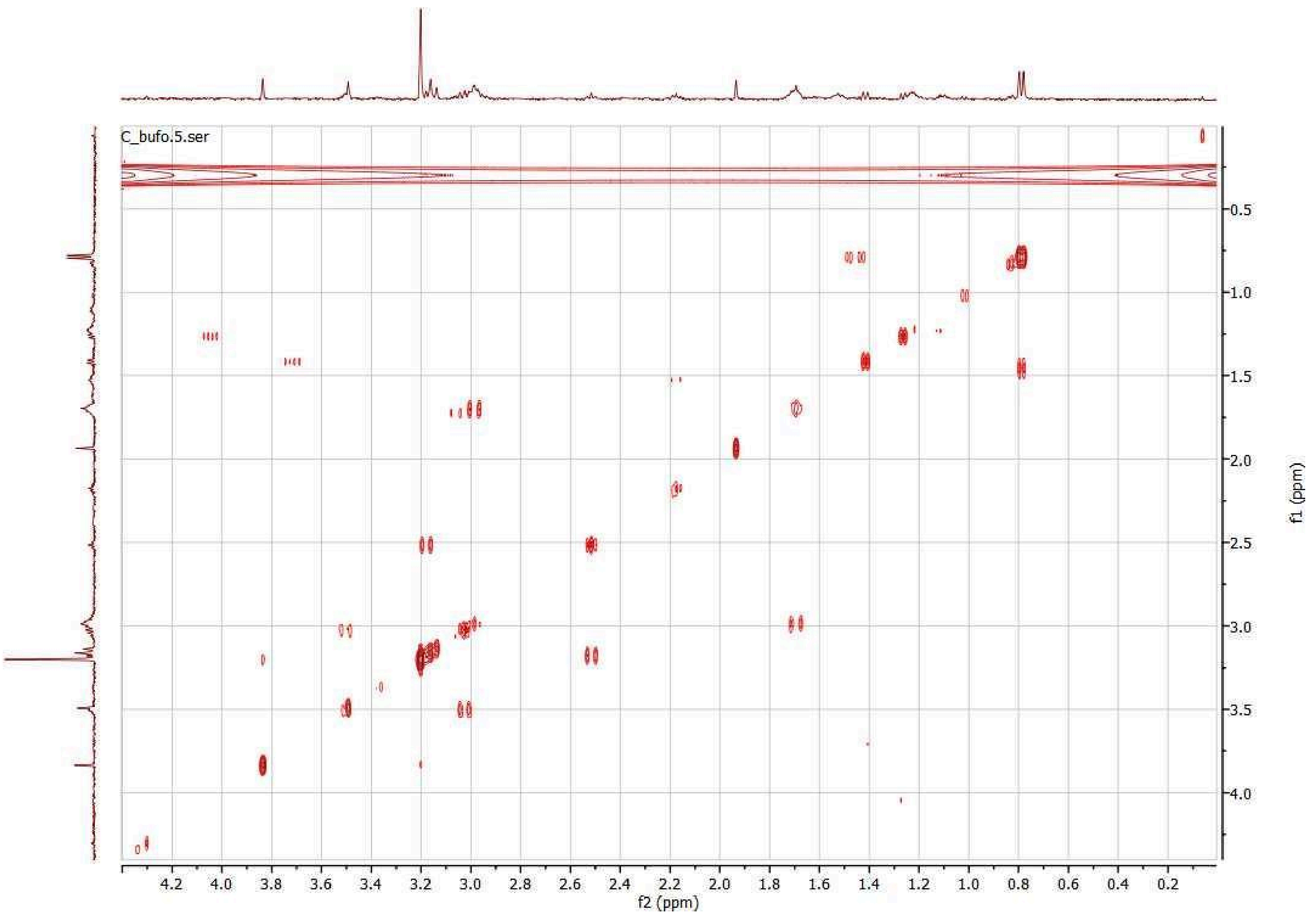

Cyrtarachne bufo – 2D COSY

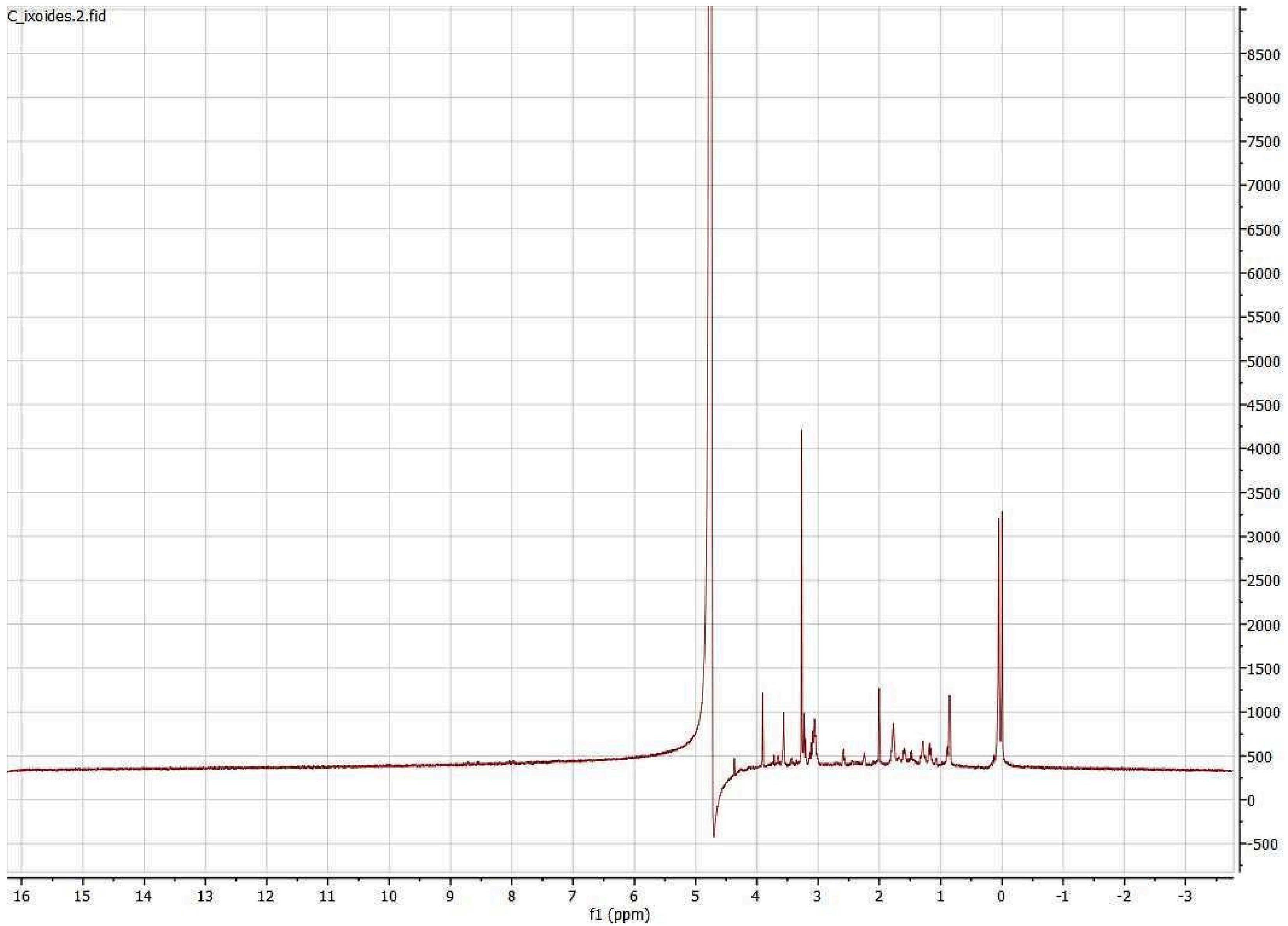
C_ixoides.2.fid
f1 (ppm)
16 15 14 13 12 11 10 9 8 7 6 5 4 3 2 1 0 -1 -2 -3
8500 8000 7500 7000 6500 6000 5500 5000 4500 4000 3500 3000 2500 2000 1500 1000 500 0 -500

Cyrtarachne ixoides – Survey Spectra

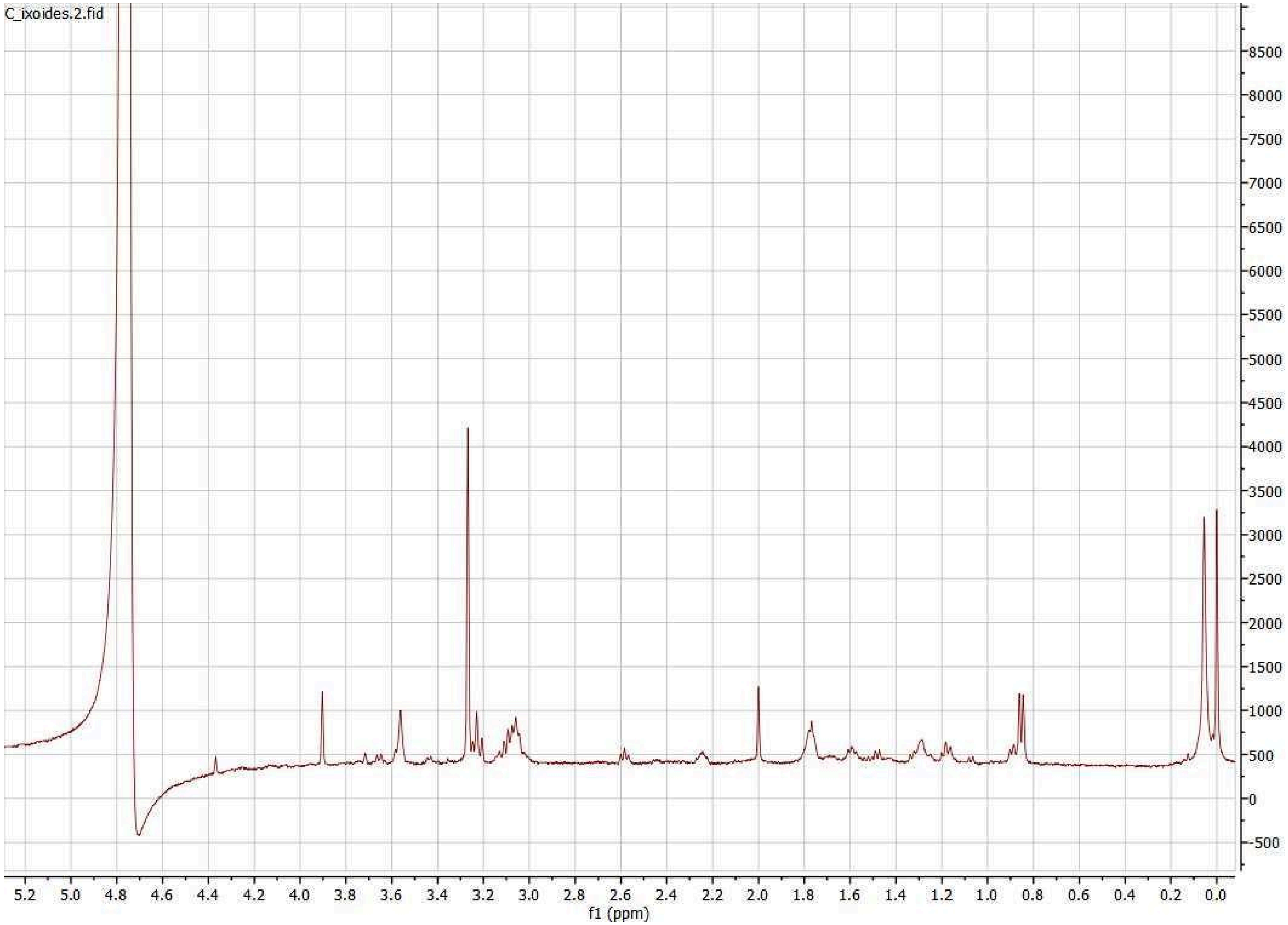

Cyrtarachne ixoides – Extended Spectra

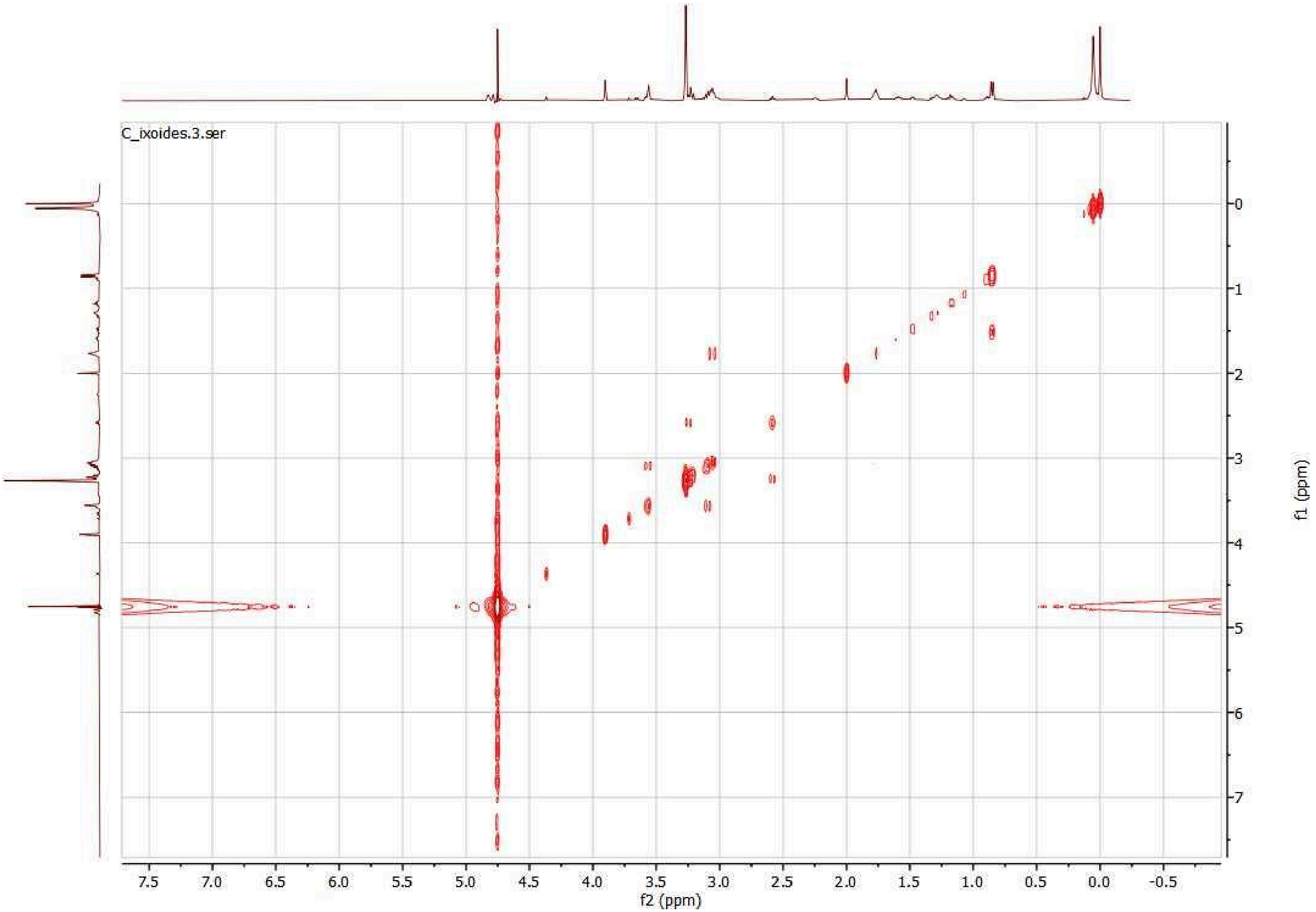

Cyrtarachne ixoides – 2D COSY

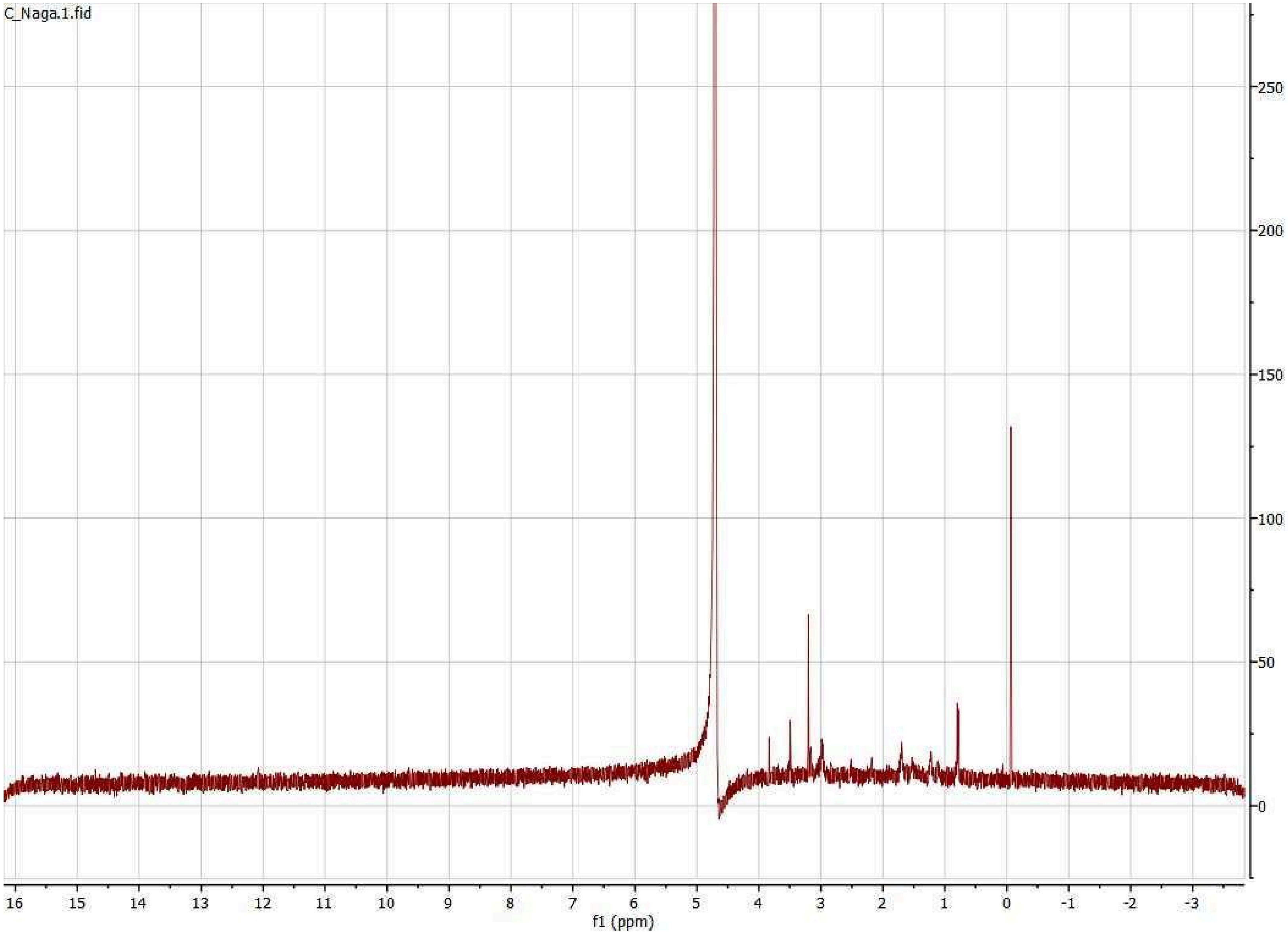

Cyrtarachne nagasakiensis – Survey Spectra

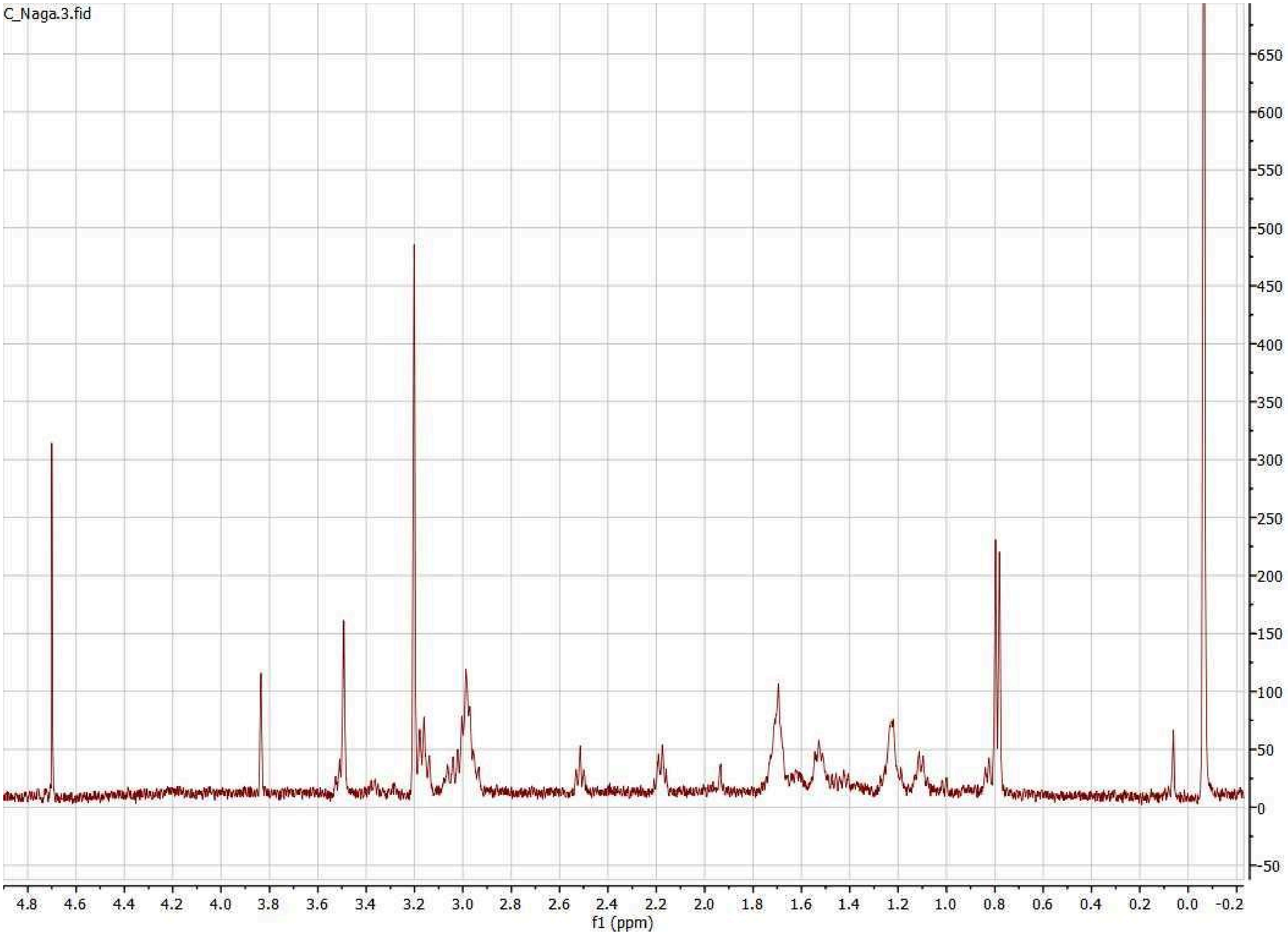

Cyrtarachne nagasakiensis – Extended Spectra

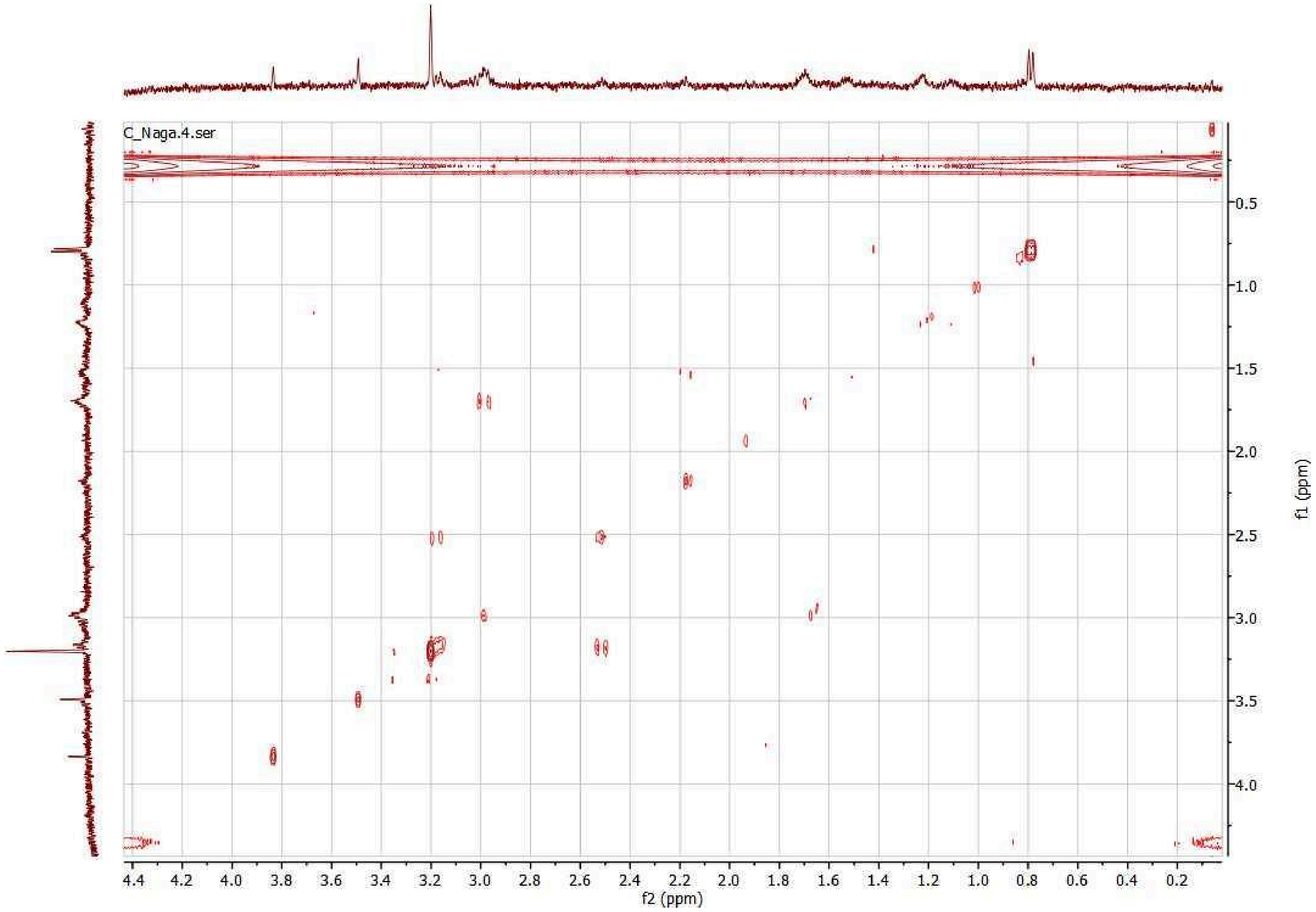

Cyrtarachne nagasakiensis – 2D COSY

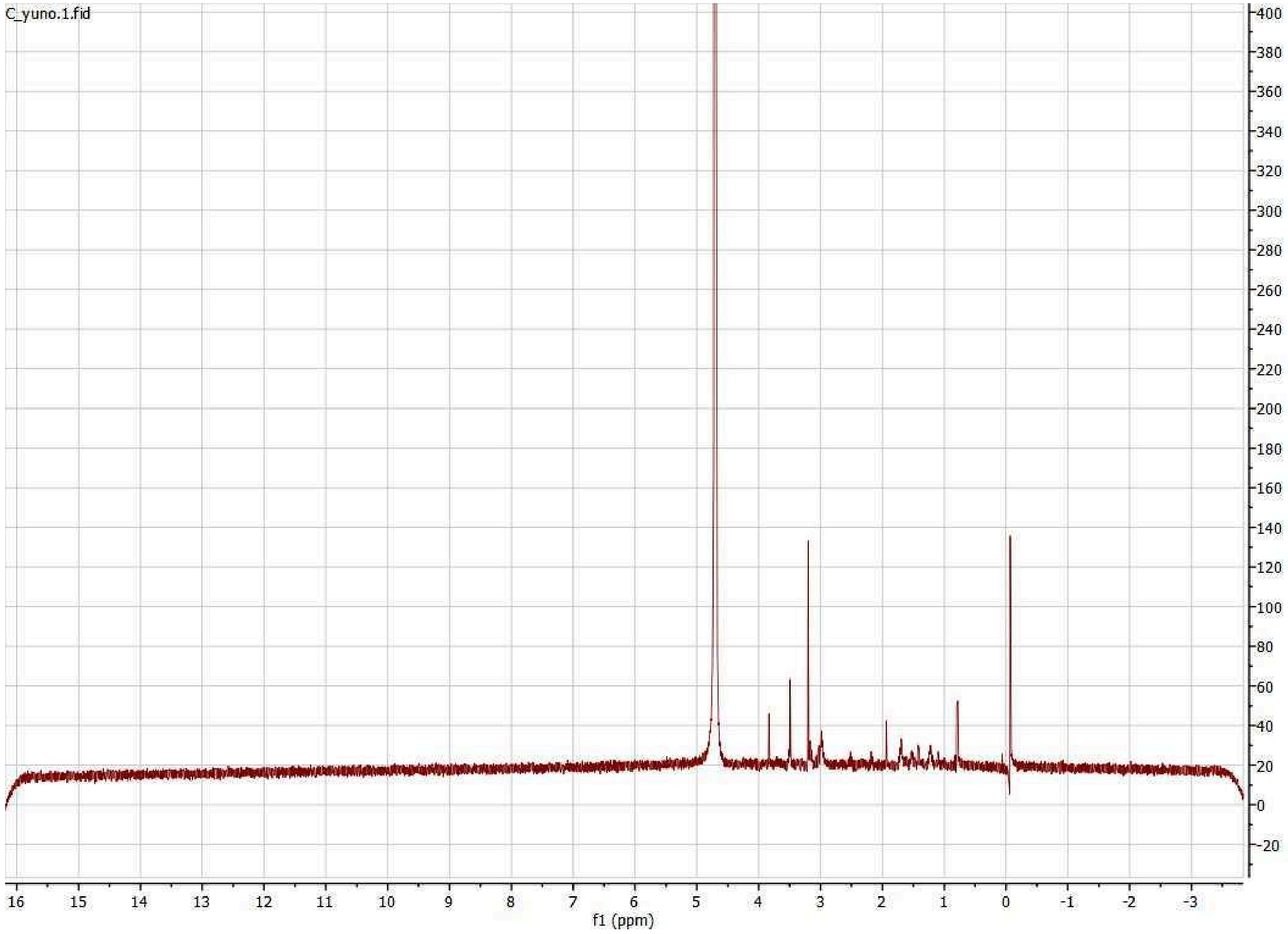

Cyrtarachne yunoharuensis – Survey Spectra

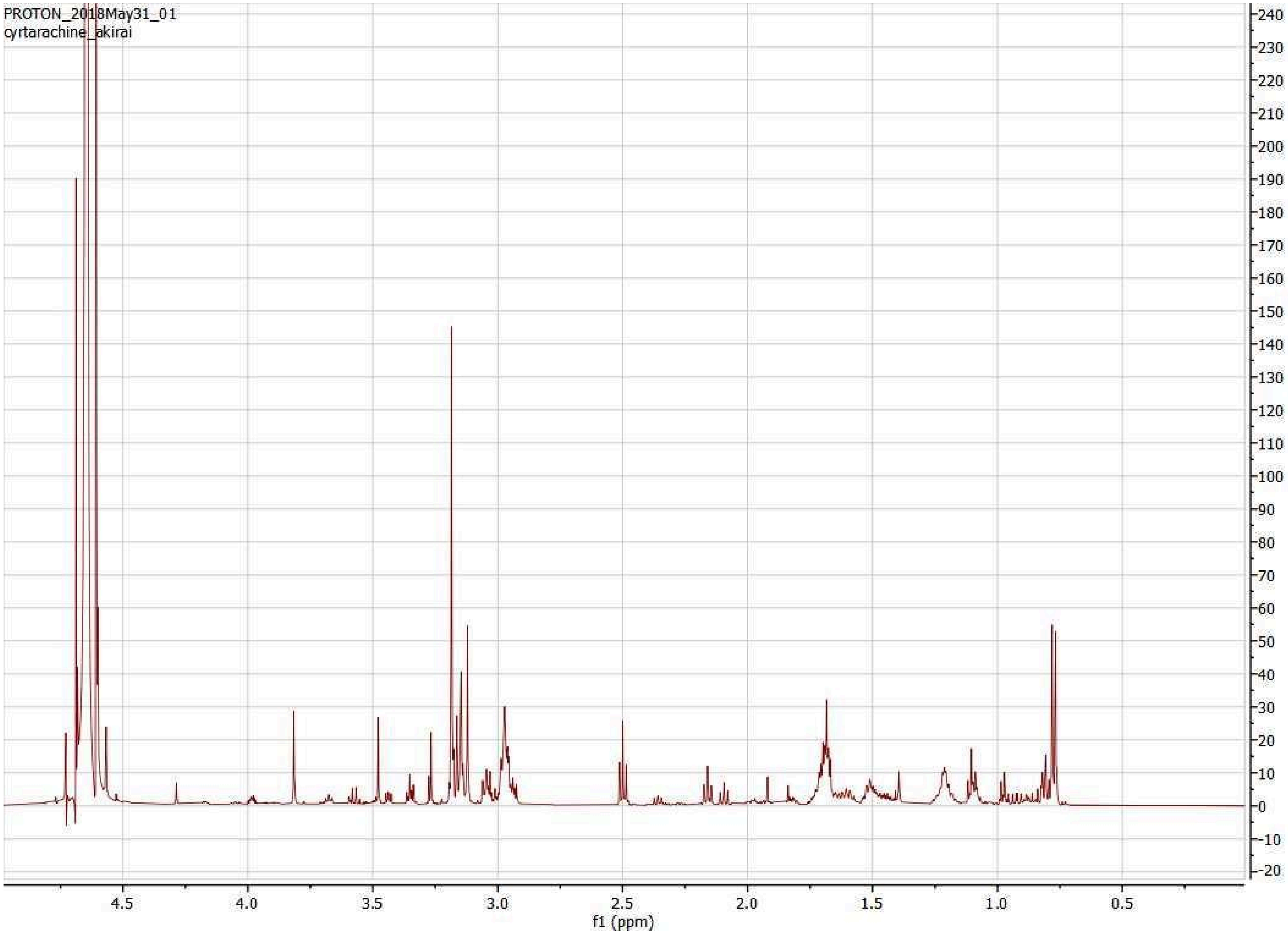

Cyrtarachne yunoharuensis – Extended Spectra

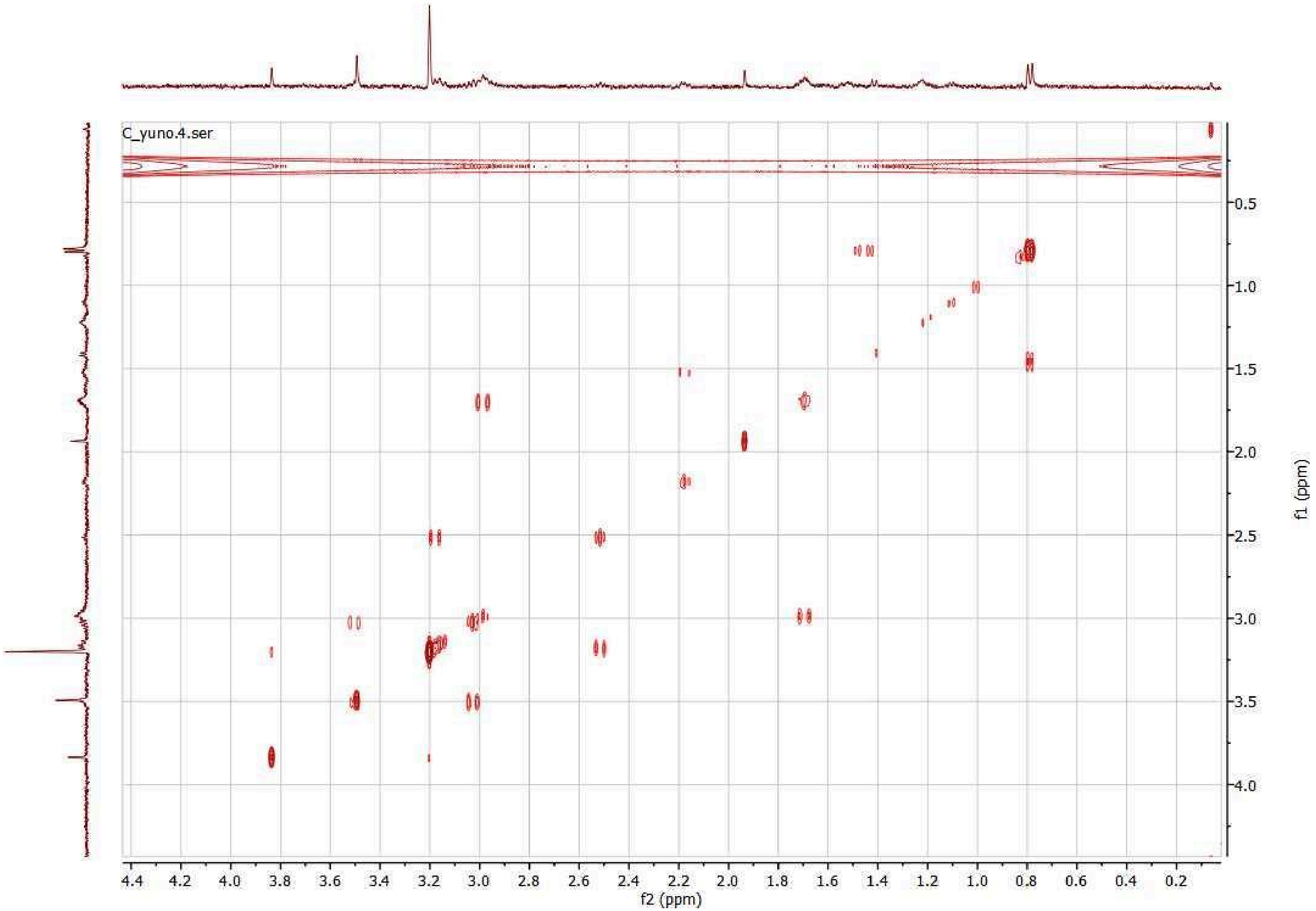

Cyrtarachne yunoharuensis – 2D COSY

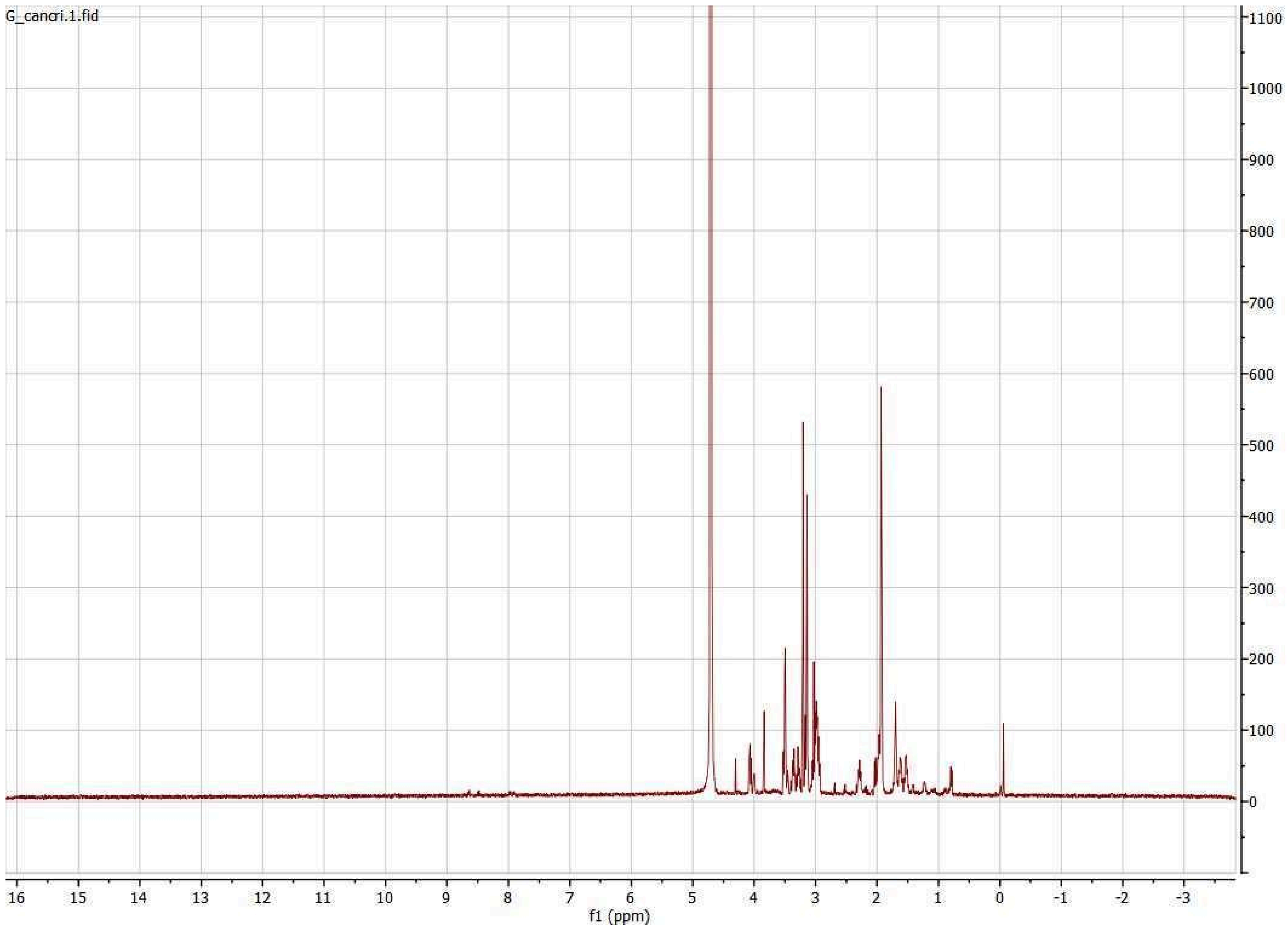
G_cancri.1.fid
f1 (ppm)

Gasteracantha cancriformis- Survey Spectra

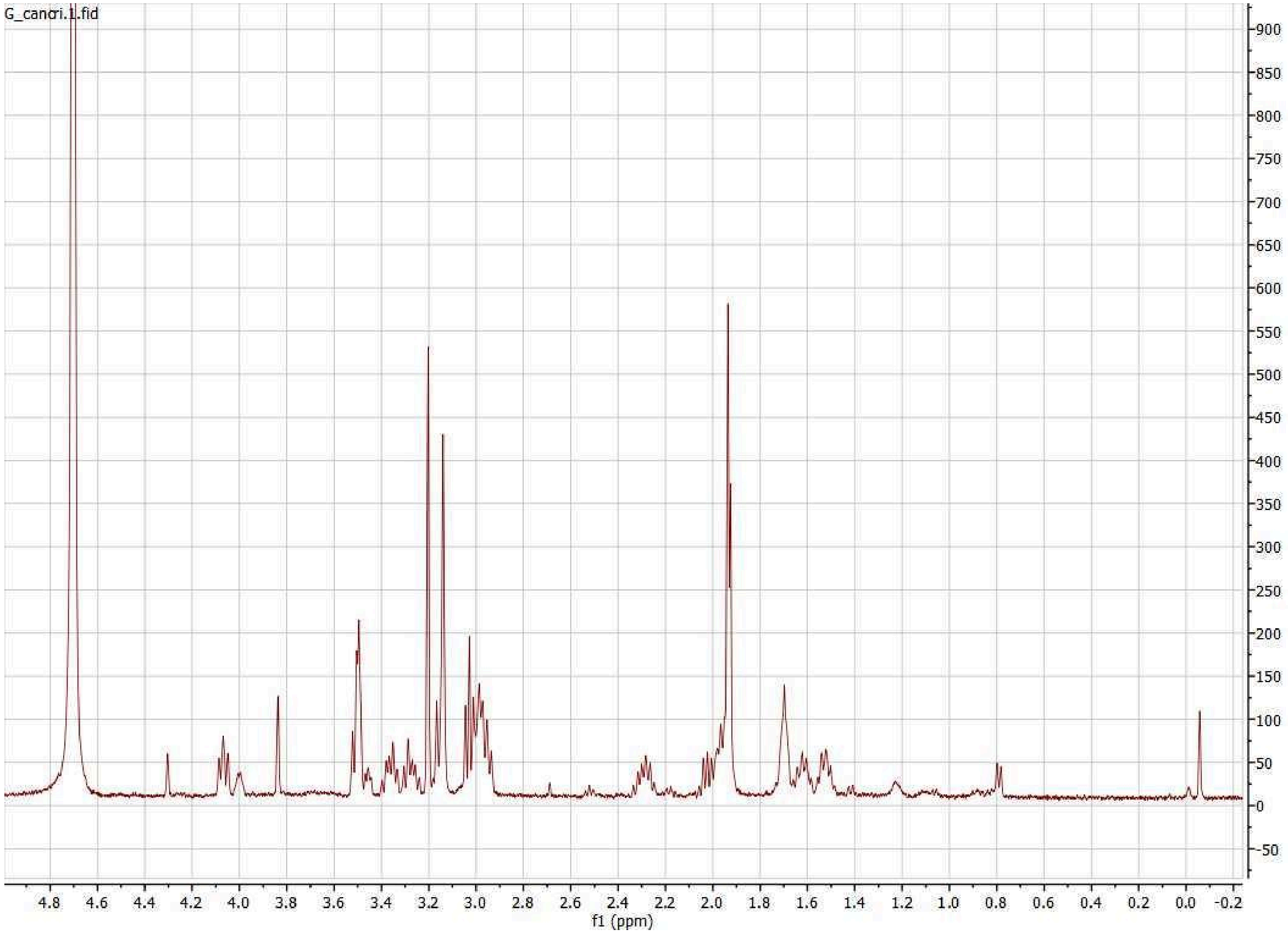

Gasteracantha cancriformis- Extended Spectra

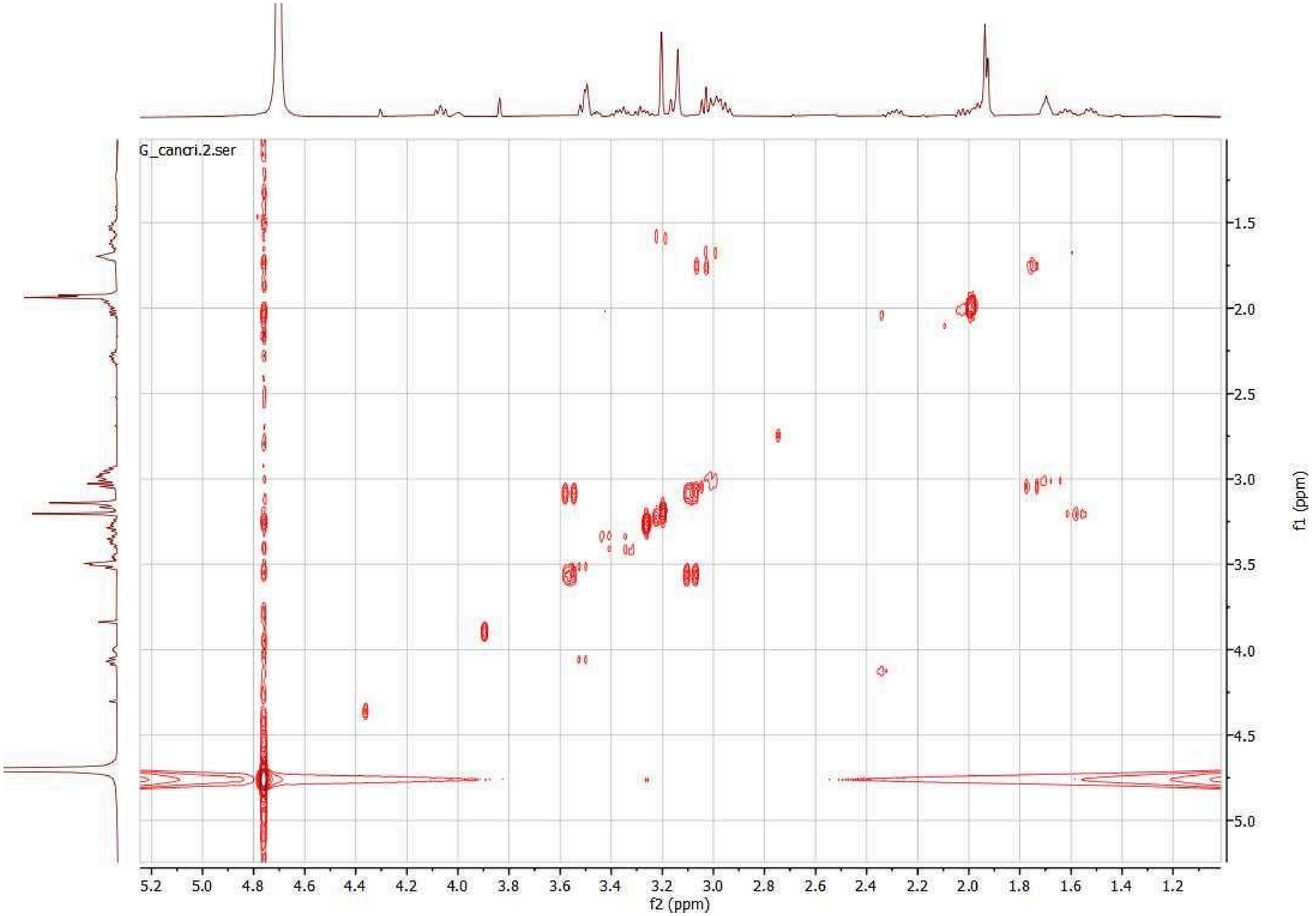

Gasteracantha cancriformis- 2D COSY

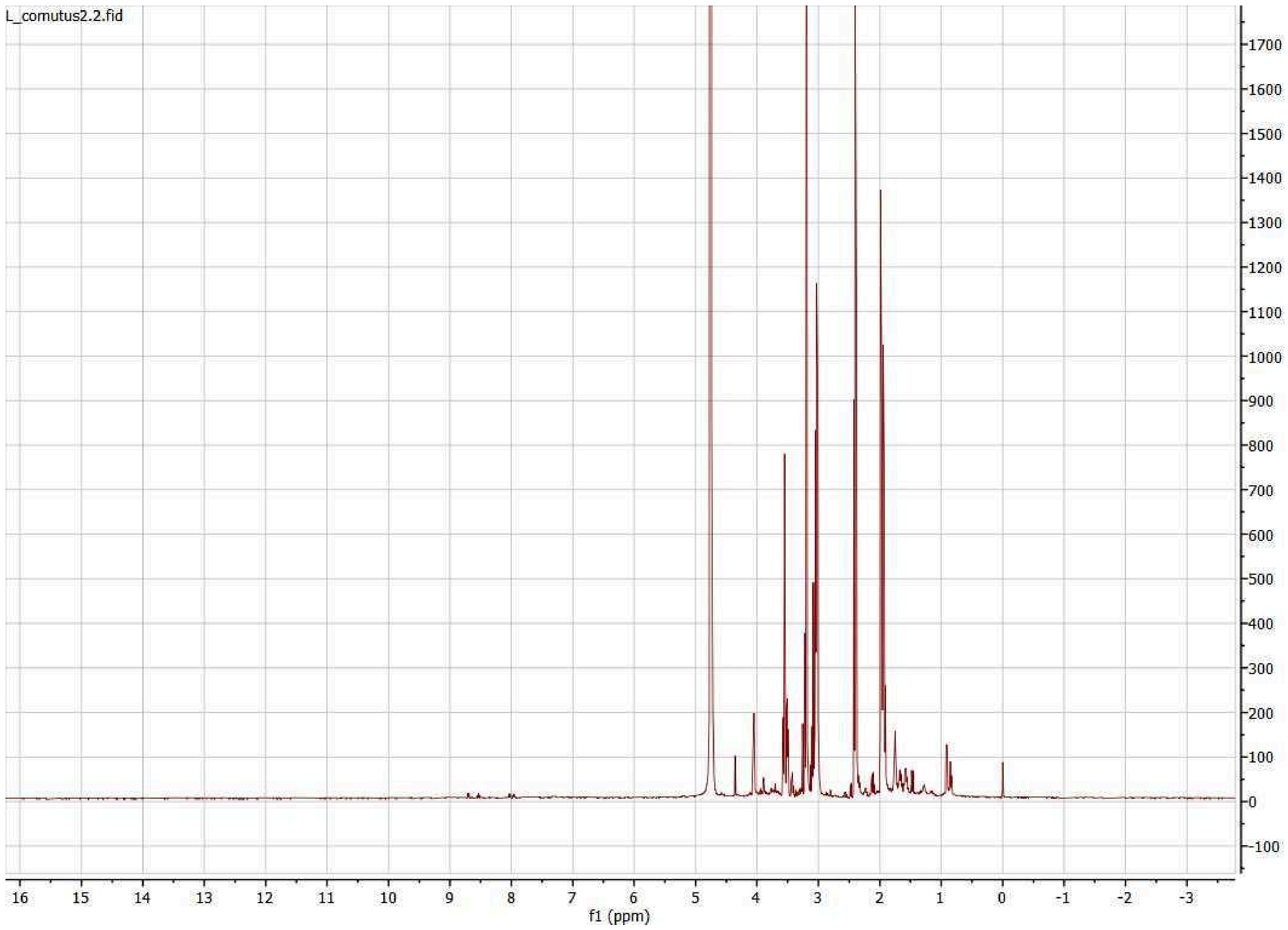

L_cornutus2.2.fid
1700
1600
1500
1400
1300
1200
1100
1000
900
800
700
600
500
400
300
200
100
0
-100
16 15 14 13 12 11 10 9 8 7 6 5 4 3 2 1 0 -1 -2 -3
f1 (ppm)

Larinioides cornutus – Survey Spectra

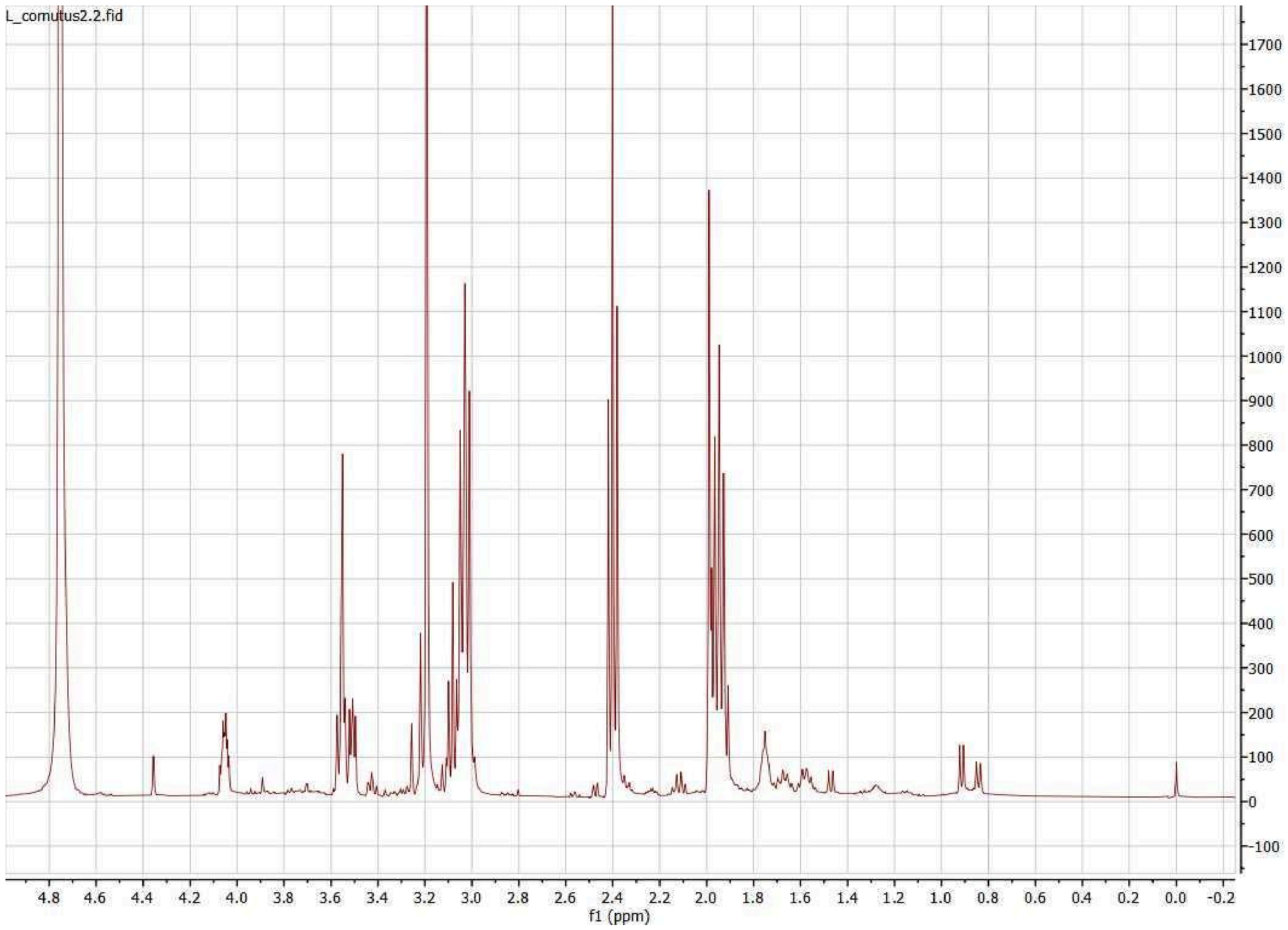
L_cornutus2.2.fid
f1 (ppm)

Larinioides cornutus – Extended Spectra

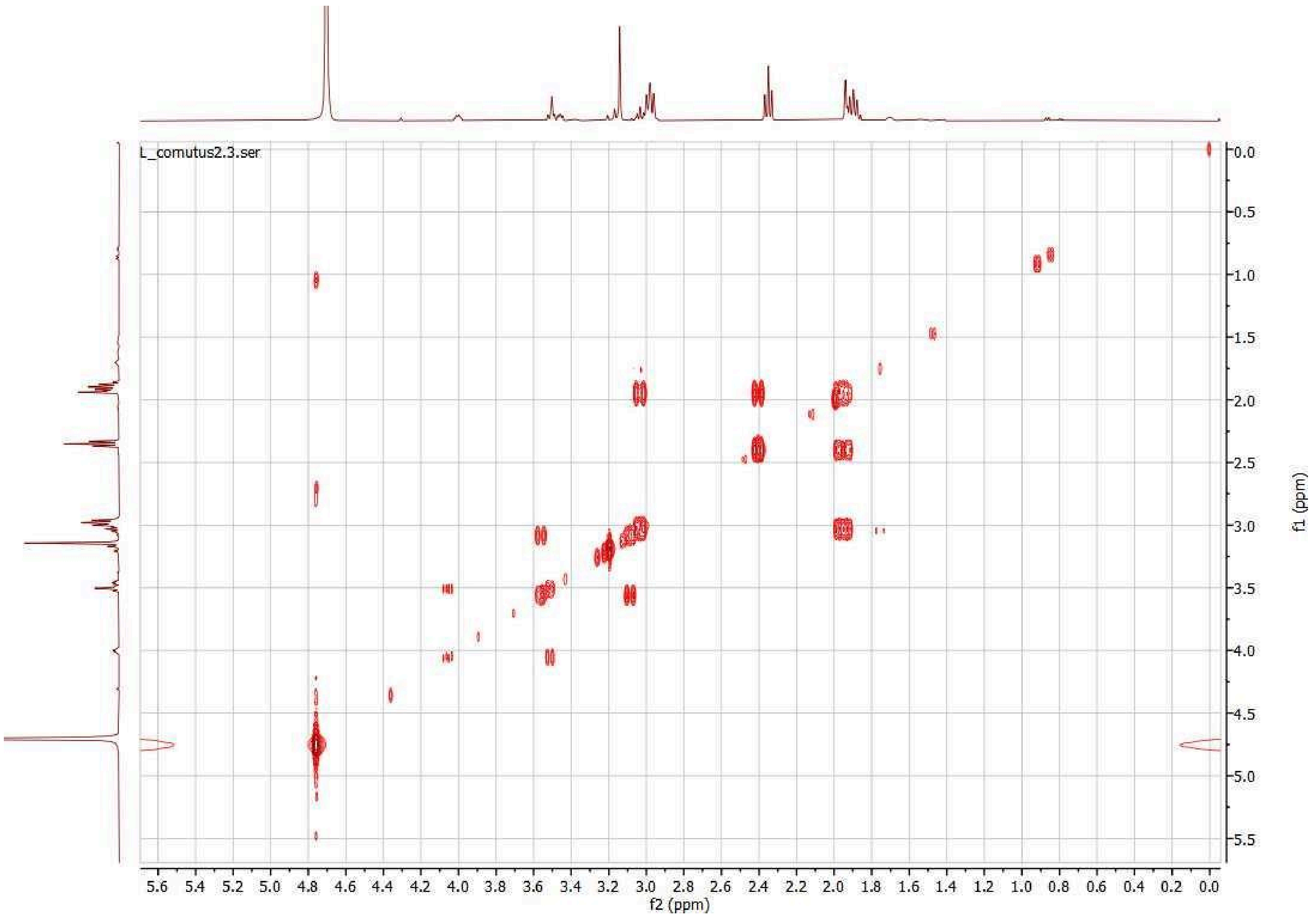

***Larinioides cornutus* – 2D COSY**

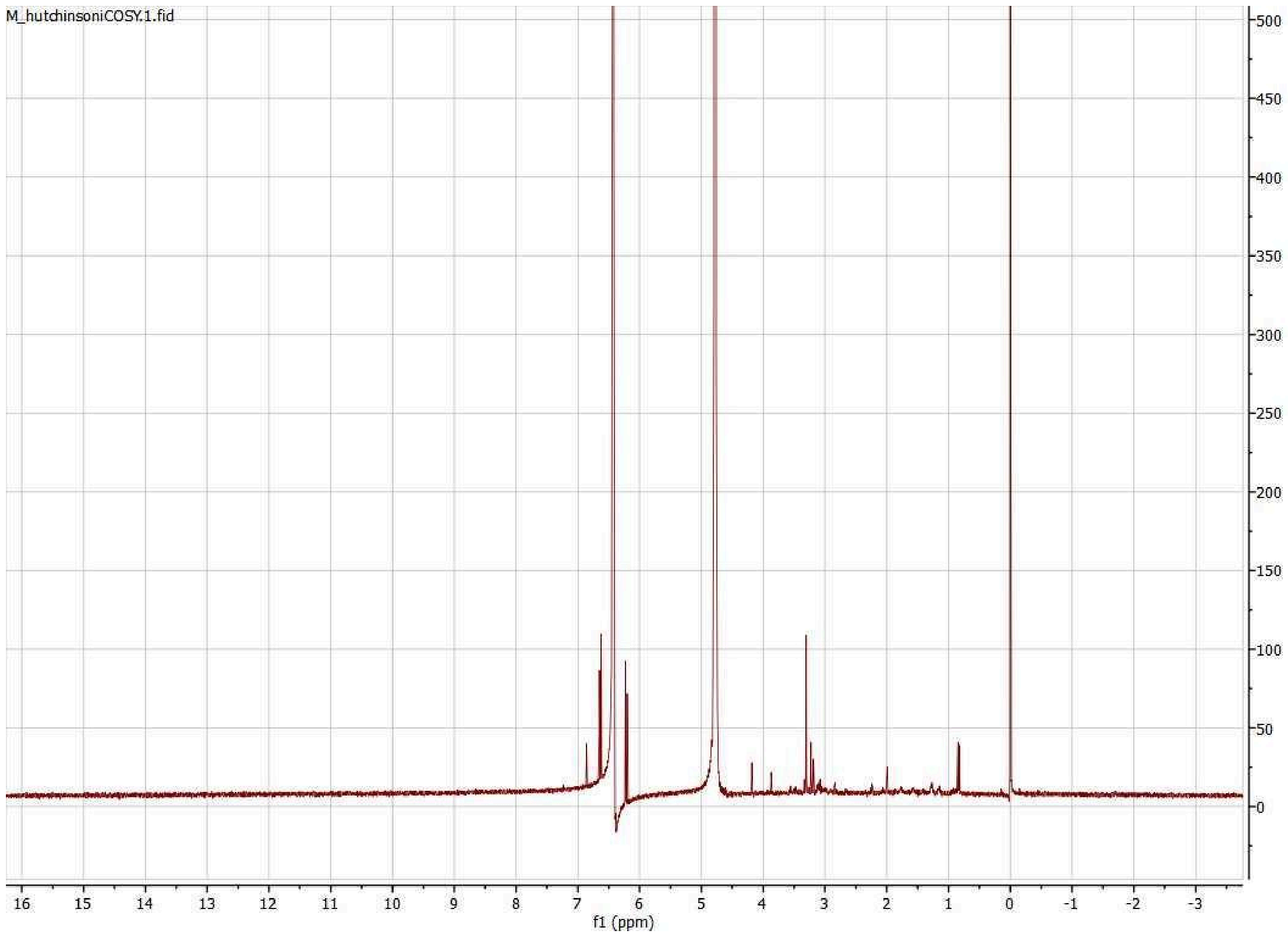

Mastophora hutchinsoni **– Survey Spectra (contains added maleic acid ~ 6.5 ppm)**

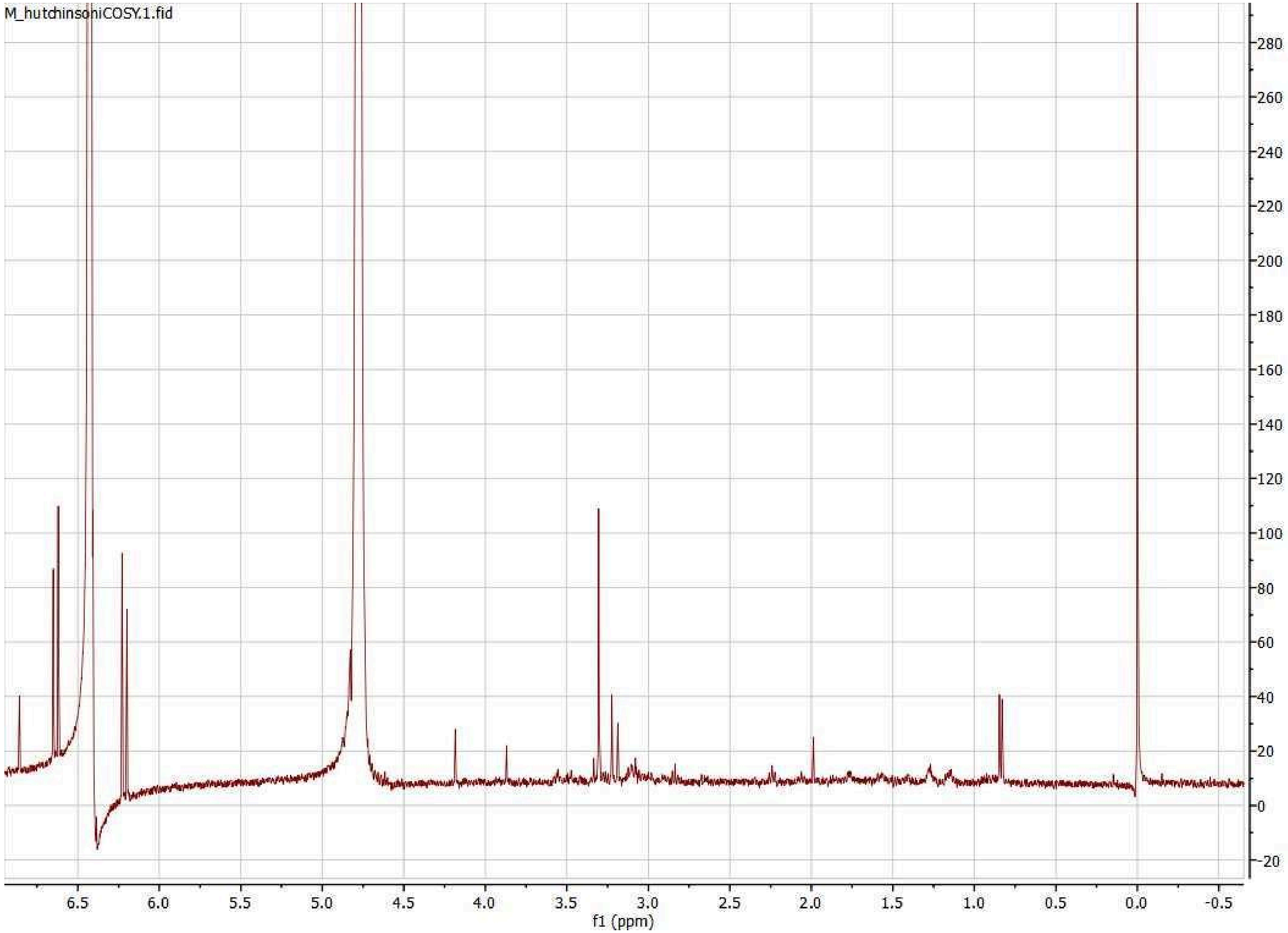

***Mastophora hutchinsoni* – Extended Spectra (contains added maleic acid ~ 6.5 ppm)**

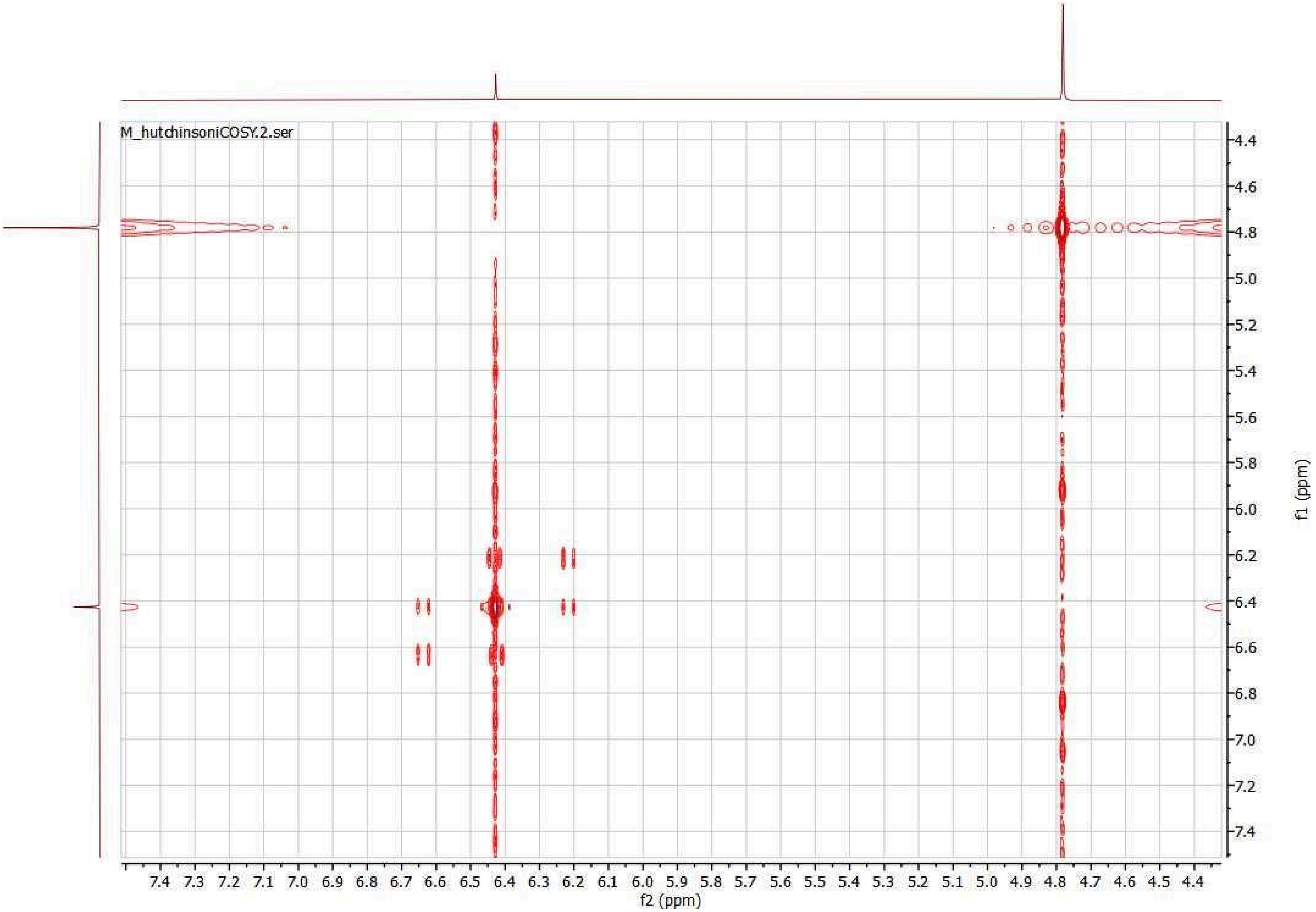

***Mastophora hutchinsoni* – 2D COSY**

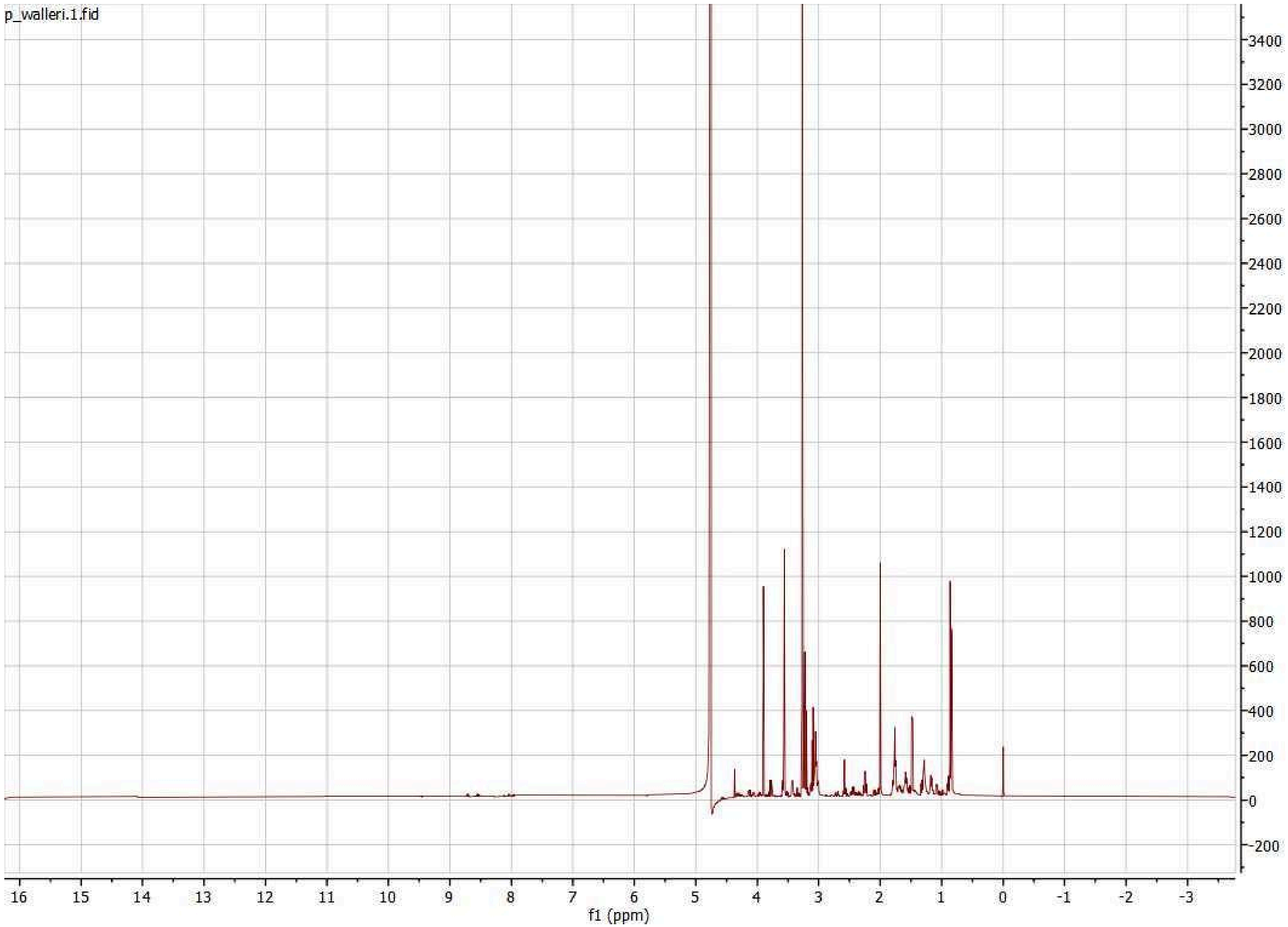

***Paraplectana walleri* – Survey Spectra**

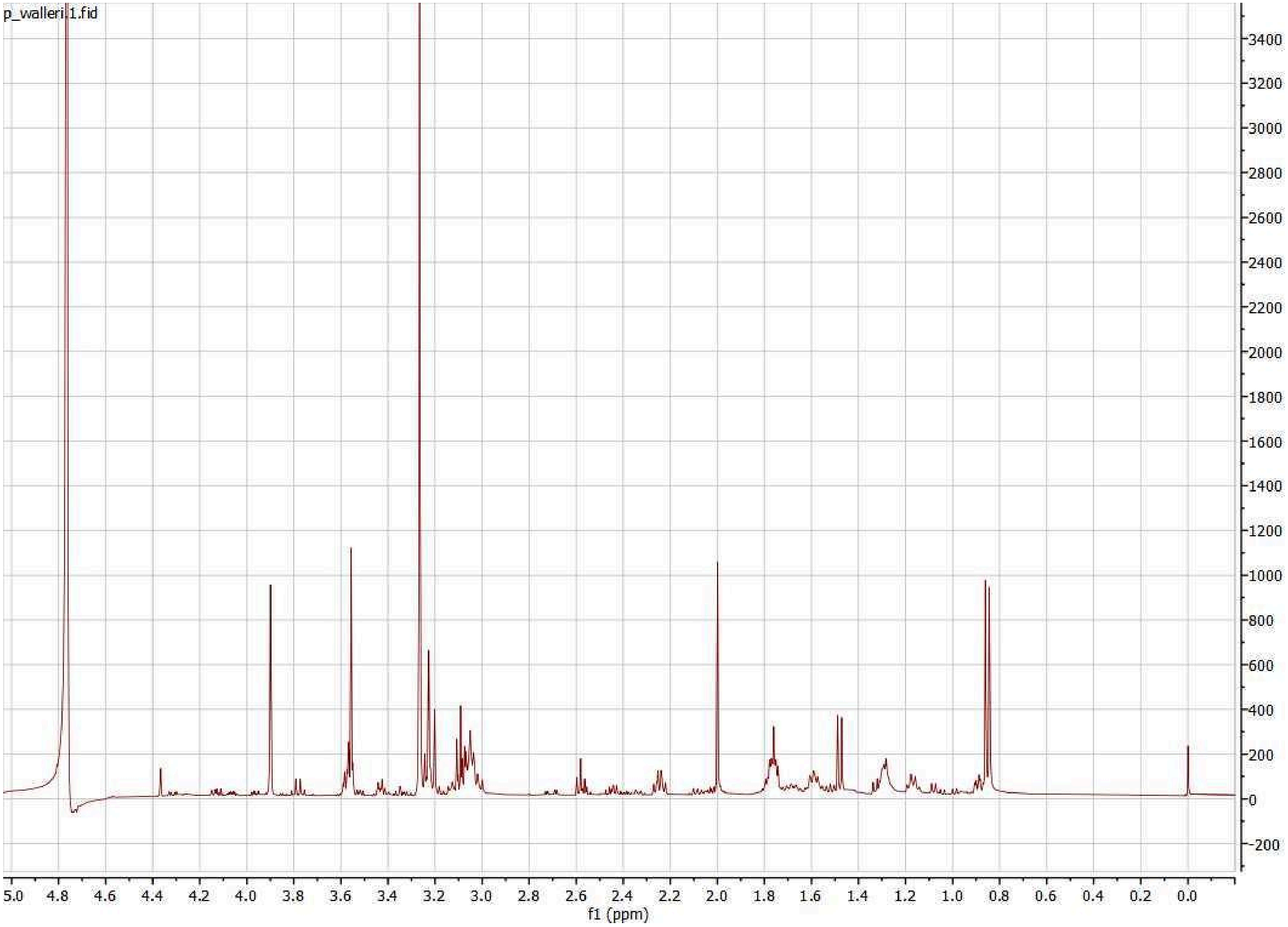

Paraplectana walleri **– Extended Spectra**

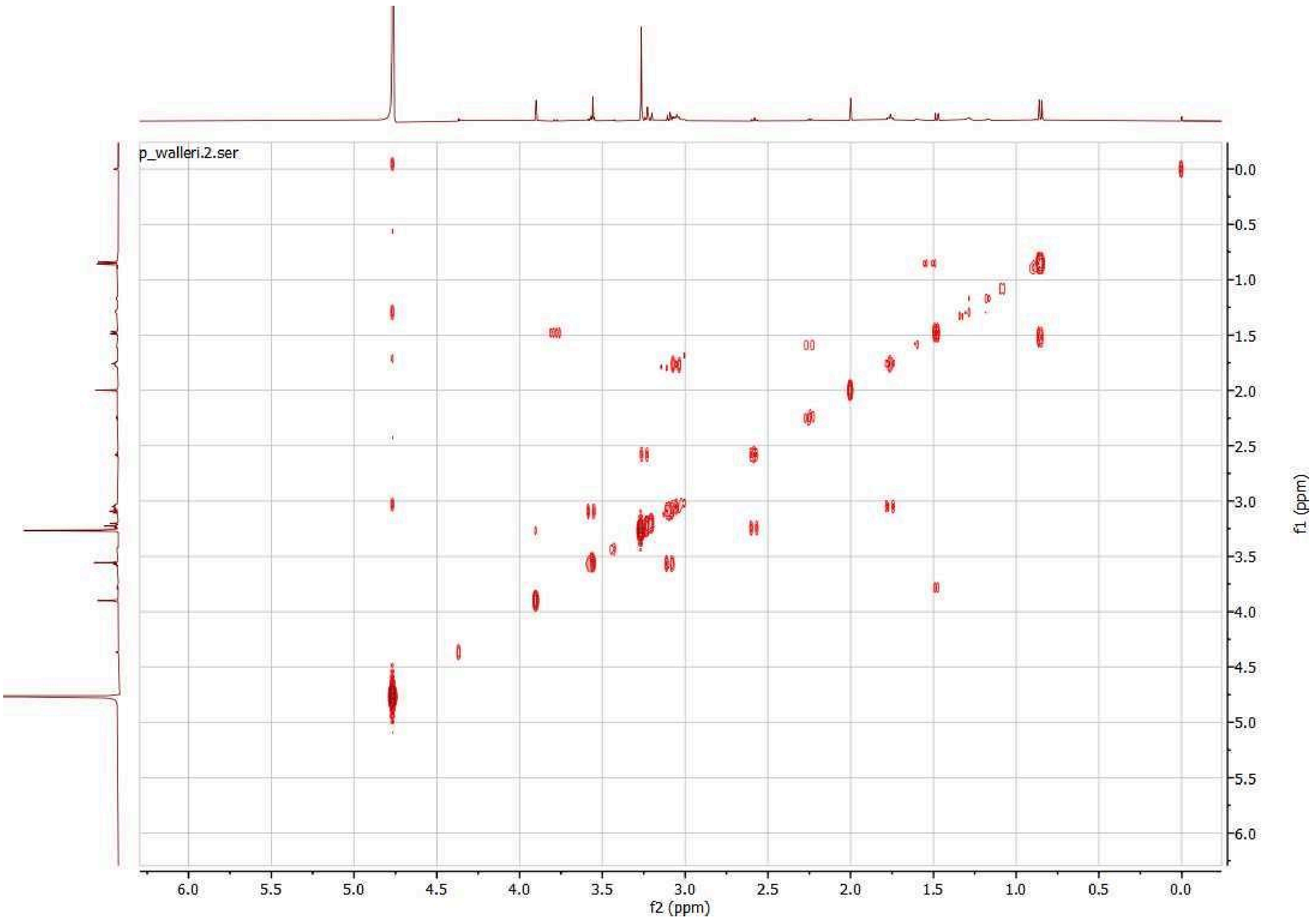
p_walleri.2.ser
f2 (ppm)
f1 (ppm)
6.0
5.5
5.0
4.5
4.0
3.5
3.0
2.5
2.0
1.5
1.0
0.5
0.0

Paraplectana walleri – 2D COSY

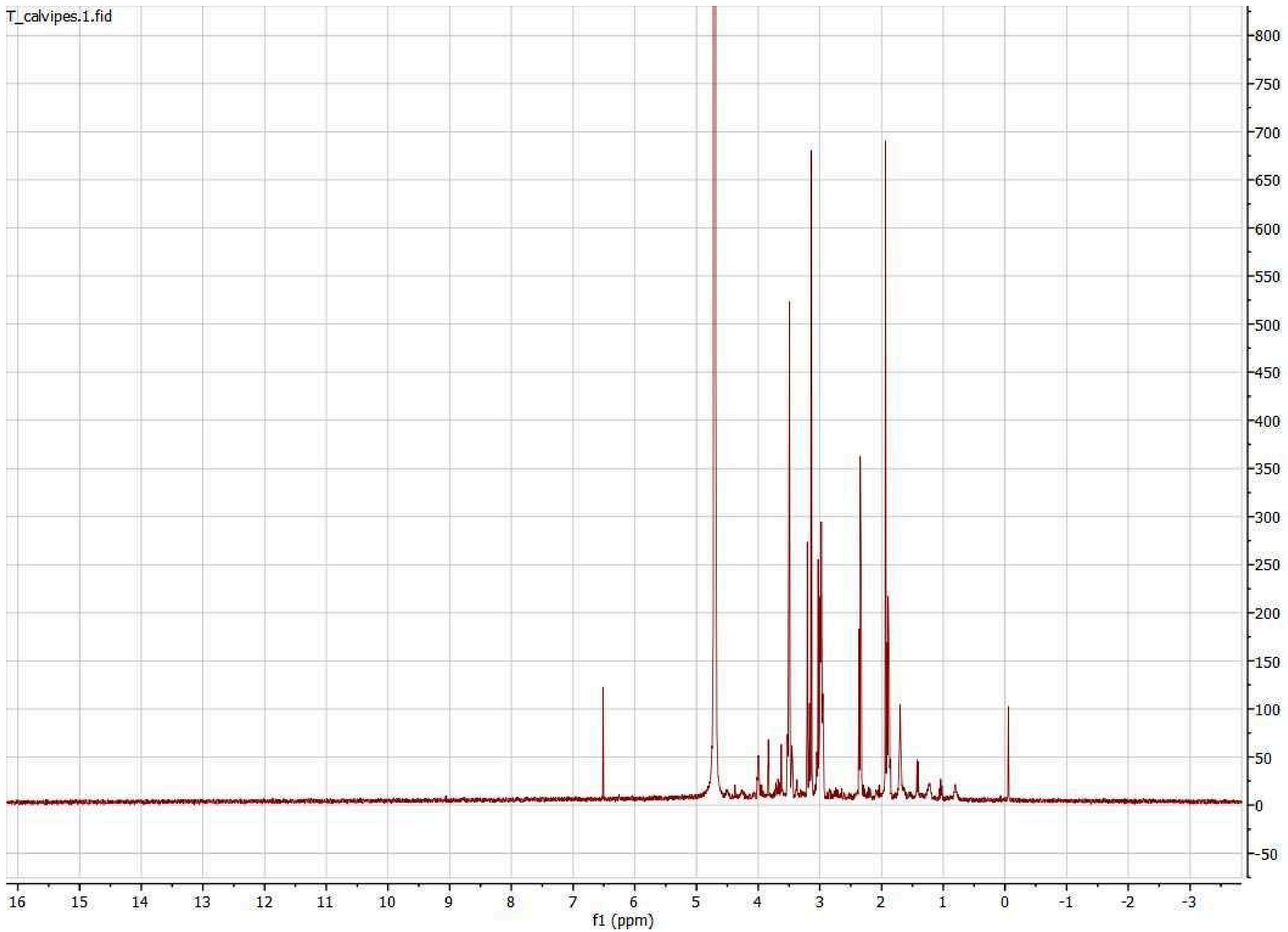
T_calvipes.1.fid
f1 (ppm)

Trichonephila clavipes – Survey Spectra

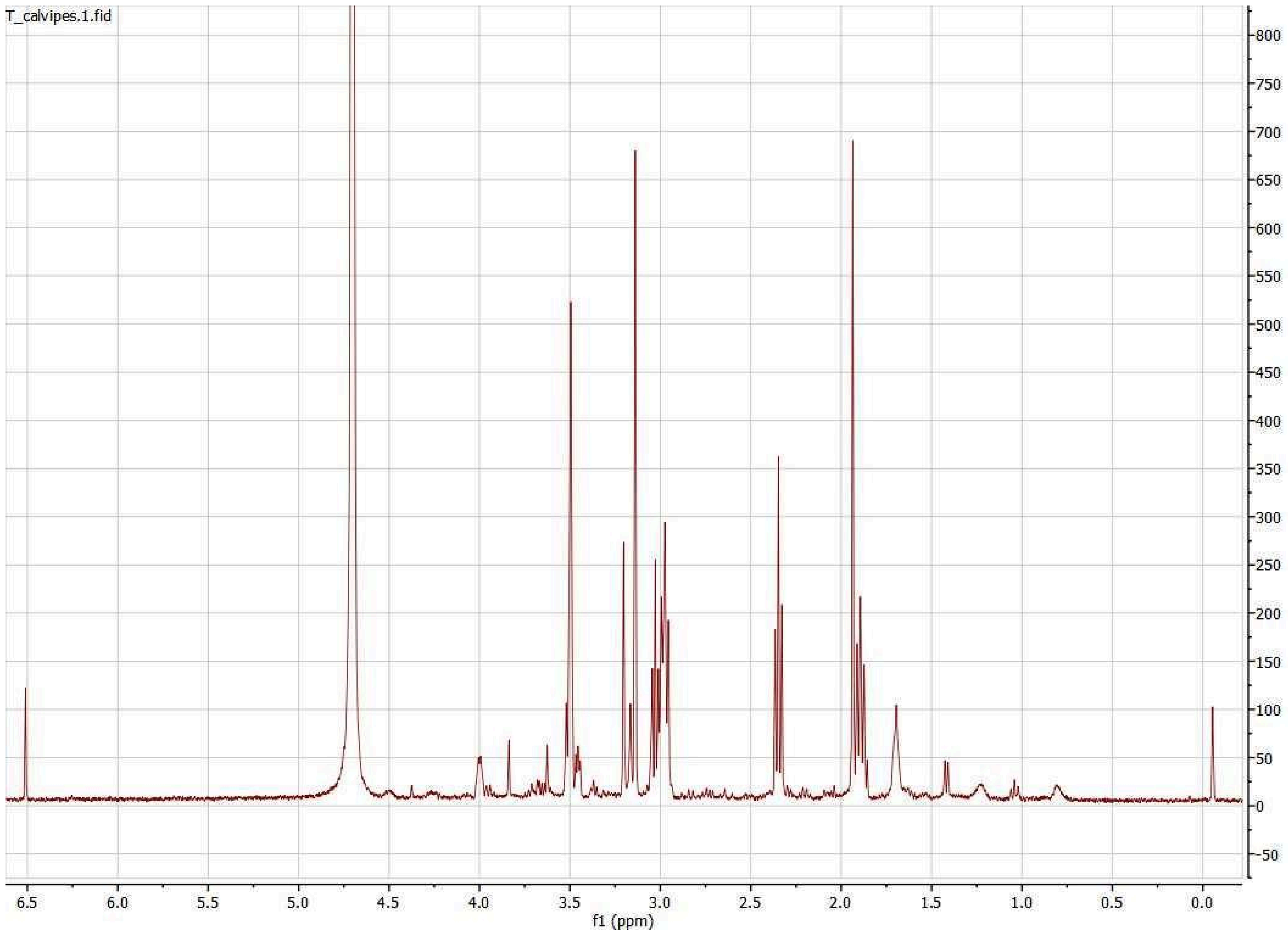
T_calvipes.1.fid
f1 (ppm)

Trichonephila clavipes – Extended Spectra

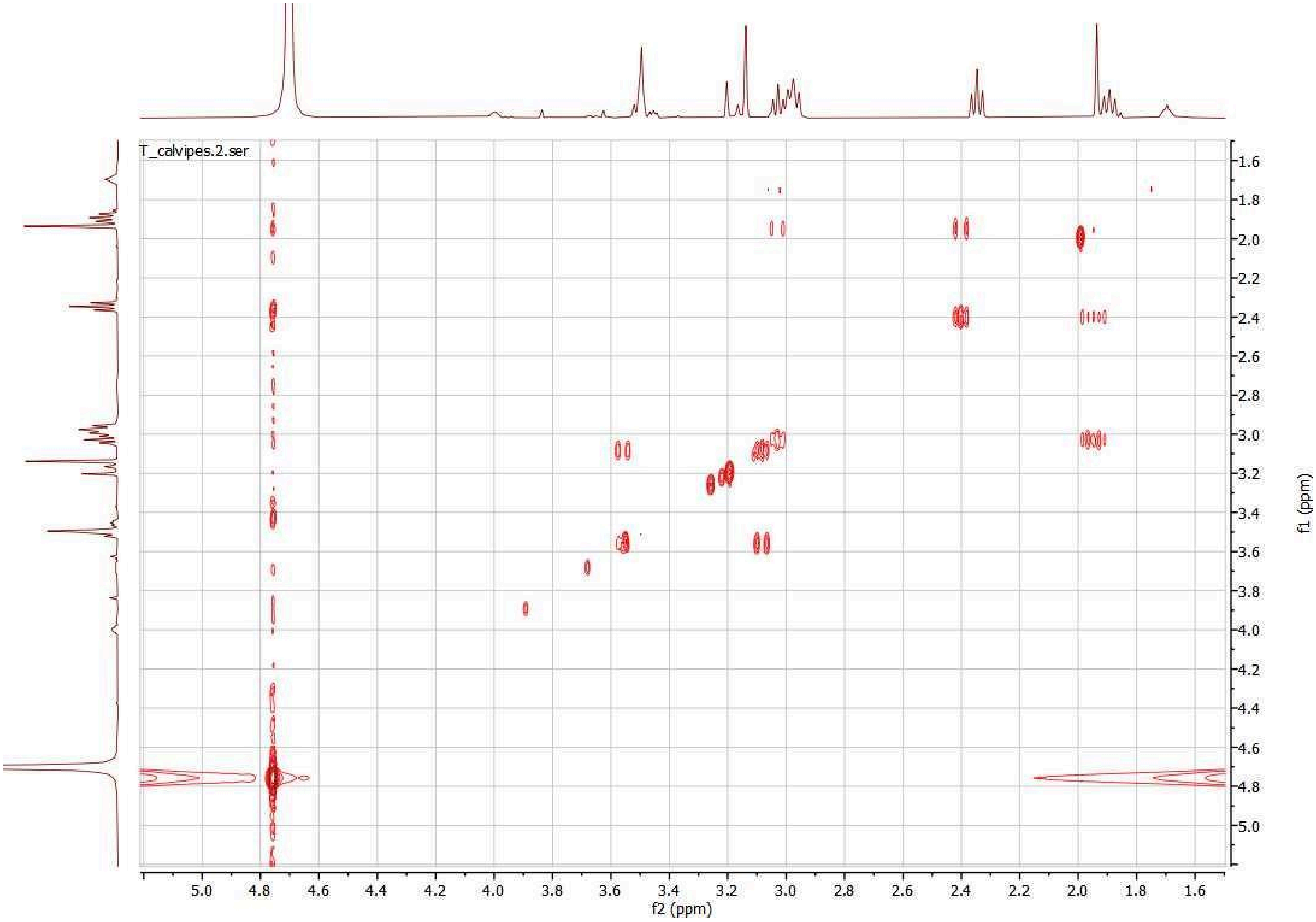

Trichonephila clavipes – 2D COSY
